# Multiplexed CRISPR-based microfluidic platform for clinical testing of respiratory viruses and identification of SARS-CoV-2 variants

**DOI:** 10.1038/s41591-022-01734-1

**Published:** 2022-02-07

**Authors:** Nicole L. Welch, Meilin Zhu, Catherine Hua, Juliane Weller, Marzieh Ezzaty Mirhashemi, Tien G. Nguyen, Sreekar Mantena, Matthew R. Bauer, Bennett M. Shaw, Cheri M. Ackerman, Sri Gowtham Thakku, Megan W. Tse, Jared Kehe, Marie-Martine Uwera, Jacqueline S. Eversley, Derek A. Bielwaski, Graham McGrath, Joseph Braidt, Jeremy Johnson, Felecia Cerrato, Gage K. Moreno, Lydia A. Krasilnikova, Brittany A. Petros, Gabrielle L. Gionet, Ewa King, Richard C. Huard, Samantha K. Jalbert, Michael L. Cleary, Nicholas A. Fitzgerald, Stacey B. Gabriel, Glen R. Gallagher, Sandra C. Smole, Lawrence C. Madoff, Catherine M. Brown, Matthew W. Keller, Malania M. Wilson, Marie K. Kirby, John R. Barnes, Daniel J. Park, Katherine J. Siddle, Christian T. Happi, Deborah T. Hung, Michael Springer, Bronwyn L. MacInnis, Jacob E. Lemieux, Eric Rosenberg, John A. Branda, Paul C. Blainey, Pardis C. Sabeti, Cameron Myhrvold

**Affiliations:** 1 Broad Institute of MIT and Harvard, Cambridge, MA USA; 2 Harvard Program in Virology, Division of Medical Sciences, Harvard Medical School, Boston, MA USA; 3 Department of Biological Engineering, Massachusetts Institute of Technology, Cambridge, MA USA; 4 Division of Infectious Diseases, Department of Medicine, Massachusetts General Hospital, Boston, MA USA; 5 Wellcome Sanger Institute, Wellcome Genome Campus, Hinxton, UK; 6 Harvard Program in Biological and Biomedical Sciences, Harvard Medical School, Boston, MA USA; 7 Division of Health Sciences and Technology, Harvard Medical School and Massachusetts Institute of Technology, Cambridge, MA USA; 8 Department of Organismic and Evolutionary Biology, Harvard University, Cambridge, MA USA; 9 Harvard/Massachusetts Institute of Technology MD-PhD Program, Harvard Medical School, Boston, MA USA; 10 Department of Systems Biology, Harvard Medical School, Boston, MA USA; 11 State Health Laboratories, Rhode Island Department of Health, Providence, RI USA; 12 Massachusetts Department of Public Health, Boston, MA USA; 13 Influenza Division, National Center for Immunization and Respiratory Diseases, Centers for Disease Control and Prevention, Atlanta, GA USA; 14 African Centre of Excellence for Genomics of Infectious Diseases, Redeemer’s University, Ede, Nigeria; 15 Department of Biological Sciences, College of Natural Sciences, Redeemer’s University, Ede, Nigeria; 16 Molecular Biology Department and Center for Computational and Integrative Biology, Massachusetts General Hospital, Boston, MA USA; 17 Department of Immunology and Infectious Disease, Harvard T.H. Chan School of Public Health, Harvard University, Boston, MA USA; 18 Department of Pathology, Massachusetts General Hospital and Harvard Medical School, Boston, MA USA; 19 Koch Institute for Integrative Cancer Research at Massachusetts Institute of Technology, Cambridge, MA USA; 20 Howard Hughes Medical Institute, Chevy Chase, MD USA; 21 Department of Molecular Biology, Princeton University, Princeton, NJ USA

**Keywords:** Biotechnology, Infectious-disease diagnostics

## Abstract

The coronavirus disease 2019 (COVID-19) pandemic has demonstrated a clear need for high-throughput, multiplexed and sensitive assays for detecting severe acute respiratory syndrome coronavirus 2 (SARS-CoV-2) and other respiratory viruses and their emerging variants. Here, we present a cost-effective virus and variant detection platform, called microfluidic Combinatorial Arrayed Reactions for Multiplexed Evaluation of Nucleic acids (mCARMEN), which combines CRISPR-based diagnostics and microfluidics with a streamlined workflow for clinical use. We developed the mCARMEN respiratory virus panel to test for up to 21 viruses, including SARS-CoV-2, other coronaviruses and both influenza strains, and demonstrated its diagnostic-grade performance on 525 patient specimens in an academic setting and 166 specimens in a clinical setting. We further developed an mCARMEN panel to enable the identification of 6 SARS-CoV-2 variant lineages, including Delta and Omicron, and evaluated it on 2,088 patient specimens with near-perfect concordance to sequencing-based variant classification. Lastly, we implemented a combined Cas13 and Cas12 approach that enables quantitative measurement of SARS-CoV-2 and influenza A viral copies in samples. The mCARMEN platform enables high-throughput surveillance of multiple viruses and variants simultaneously, enabling rapid detection of SARS-CoV-2 variants.

## Abstract (web summary)

A microfluidic CRISPR-based platform enables multiplexed and high-throughput identification of a wide range of respiratory viruses, including the ability to identify specific SARS-CoV-2 variants such as Delta and Omicron.

## Main

COVID-19 has exposed critical gaps in our global infectious disease diagnostic and surveillance capacity^1^. The pandemic rapidly necessitated high-throughput diagnostics to test large populations^2^, yet early diagnostic efforts met technical challenges that cost the United States precious time in its early response^3^. Other challenges developed as the pandemic progressed that point toward an additional need for highly multiplexed surveillance technologies. These challenges include the cocirculating human respiratory viruses that cause symptoms similar to COVID-19 (refs. ^4,5^) and emerging SARS-CoV-2 variants of concern (VOCs) with mutations that impact viral fitness and clinical disease prognosis^6,7^.

An ideal diagnostic method would have surveillance capabilities to process hundreds of patient samples simultaneously, detect multiple viruses, differentiate between viral variants and quantify viral load^8,9^; yet no such test currently exists. As it stands, there is a trade-off between clinically approved high-throughput diagnostics and multiplexed methods in the number of patient samples and/or pathogens tested simultaneously^10–12^. For example, quantitative PCR with reverse transcription (RT–qPCR) is high-throughput by testing at least 88 samples but for 1–3 analytes at a time; multiplexed techniques such as Cepheid Xpert Xpress can detect 4 respiratory viruses in up to 16 samples per run and BioFire can detect 22 respiratory pathogens in 1 sample simultaneously^13^. Only a few clinical diagnostic methods comprehensively detect SARS-CoV-2 variant mutations^14–16^, which is why this has largely been achieved through next-generation sequencing (NGS)^17,18^, though it is time-consuming, expensive and requires bioinformatic expertise to interpret^6,19–22^.

CRISPR-based diagnostics offer an alternative approach to detecting multiple viruses and variants^23–25^. The CRISPR effector proteins Cas12 (refs. ^26,27^) or Cas13 (refs. ^28,29^) activate upon CRISPR RNA (crRNA) target binding, which unleashes their collateral cleavage activity on a fluorescent reporter for detection of viral nucleic acids^30–34^. The crRNA target binding events are highly specific and altered by the presence of sequence variation. Maximally active crRNA design has been accelerated by machine learning and other computational methods^35^. Nonetheless, most CRISPR diagnostics detect one to three targets per sample^30,36–39^, which is not sufficient for differential diagnosis via comprehensive microbe or variant identification.

To scale up the capabilities of CRISPR-based diagnostics, we developed CARMEN^40^, which parallelizes nucleic acid detection. The first generation of CARMEN, referred to in this article as CARMEN v.1, could detect 169 human-associated viruses in 8 samples simultaneously. In CARMEN v.1, samples and Cas13–crRNA complexes are separately confined for barcoding and emulsification before pairwise droplet combination for detection by fluorescence microscopy. This allows each sample to be tested against every crRNA. CARMEN v.1 is a powerful proof of concept for multiplexed CRISPR-based detection but it is difficult to use in a clinical setting given its use of custom-made imaging chips and readout hardware, manually intensive 8–10 h workflow and low-throughput sample evaluation.

To fulfill the public health need for a clinically relevant surveillance technology that detects multiple viruses and variants quickly, we developed mCARMEN. mCARMEN builds on CARMEN v.1 and uses commercially available Fluidigm microfluidics and instrumentation. To our knowledge, mCARMEN is the only diagnostic method that combines surveillance capabilities into a single technology platform with the ability to test hundreds of samples in a day for multiple respiratory viruses and variants, while also being able to quantify viral genomic copies.

## Results

### CARMEN implementation on Fluidigm for respiratory virus detection

**Fig. 1:**
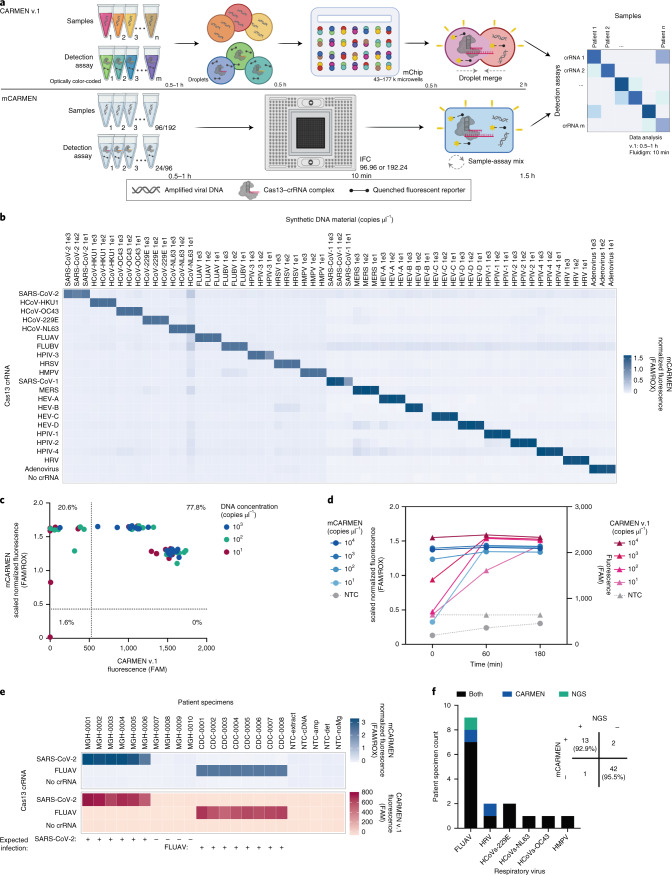
Implementation of CARMEN using a microfluidic system improves sensitivity and speed. **a**, Schematic of CARMEN v.1 (top) and mCARMEN (bottom) workflows. **b**, Heatmap showing mCARMEN fluorescent data across 21 human respiratory viruses (synthetic DNA fragments and corresponding viral Cas13 crRNAs) at 1 h post-reaction initiation, which were serially diluted from 10^3^ to 10^1^ copies μl^−1^ and amplified using 2 separate primer pools. All samples were background-subtracted from the no target control (NTC)-noMg negative control. **c**, Concordance between CARMEN v.1 and mCARMEN from **b**. Blue: targets at 10^3^ copies μl^−1^; green: targets at 10^2^ copies μl^−1^; red: targets at 10^1^ copies μl^−1^. **d**, Fluorescence kinetics of amplified SARS-CoV-2 DNA gene fragments from 10^4^–10^1^ copies μl^−1^ at 0, 1 and 3 h post-reaction initiation. Blue: mCARMEN; red: CARMEN v.1. **e**, A 21-human respiratory virus panel was tested on clinical specimens from 6 SARS-CoV-2-positive, 4 SARS-CoV-2-negative nasopharyngeal (NP) swabs and 8 FLUAV-positive specimens, collected before December 2019, and 5 NTCs. The heatmap shows fluorescent signals from SARS-CoV-2 crRNA, FLUAV crRNA and no crRNA control. Blue: mCARMEN at 1 h post-reaction initiation; red: CARMEN v.1 at 3 h post-reaction initiation. **f**, Concordance of mCARMEN and NGS on 58 suspected respiratory virus-infected patient specimens collected before December 2019 shown as a bar graph; overall concordance is shown as a confusion matrix. Black: detected by both mCARMEN and NGS; blue: detected by mCARMEN only; green: detected by NGS only. mCARMEN values are shown as the normalized fluorescence signal (FAM/ROX) (FAM signal divided by the signal for the passive reference dye, ROX, 1 h). CARMEN v.1 values are shown as the raw fluorescence signal (FAM). NTC-extract: no target control taken through extraction, cDNA synthesis, amplification, and detection; NTC-cDNA: no target control taken through cDNA synthesis, amplification and detection; NTC-amp: no target control taken through amplification and detection; NTC-det: no target control taken through detection; NTC-noMg: no target control expected to have no fluorescent signal due to lack on Mg2^+^ needed to activate Cas13.

**Extended Data Fig. 1:**
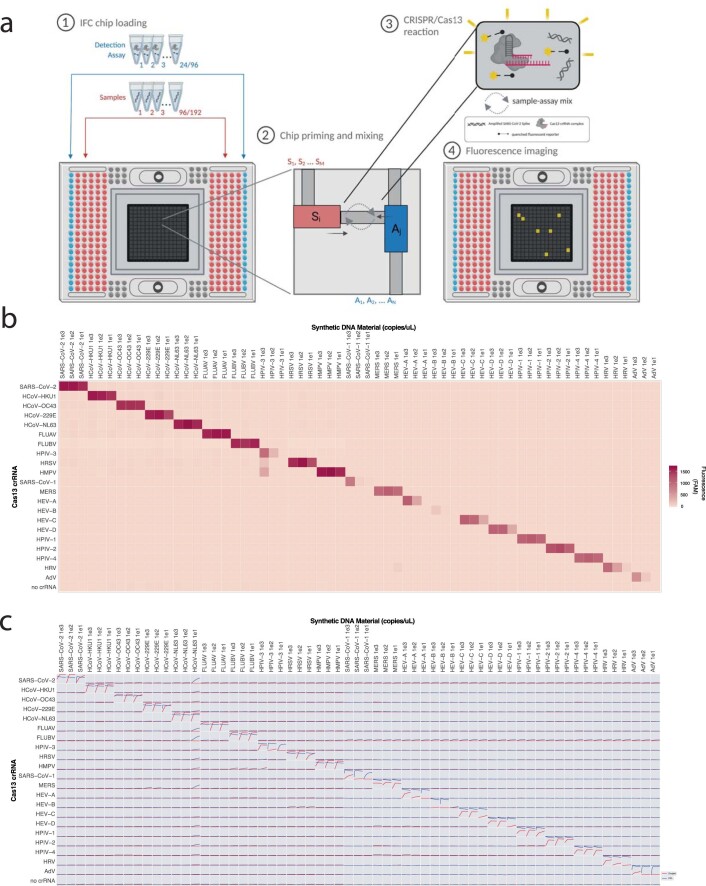
Detection of 21 respiratory viruses by mCARMEN and CARMEN v.1. **a**, Detailed schematic of loading and running 192.24 IFC in mCARMEN workflow. (Step 1) Up to 196 pre-amplified samples are added in the red microwells of the Fluidigm 192.24 Dynamic Array IFC. Up to 24 detection assay reactions containing Cas13-crRNA complexes are added on the sides of the IFC shown in blue. Control line, actuation, and pressure fluids are added according to manufacturer’s guidelines. (Step 2) The IFC is loaded in a controller where the load and mix protocol is selected. The controller automatically moves nanoliter volumes of reagent, sample, and assay mixes through individual chambers on the IFC until reaching a predetermined chamber in the chip. In the last minute of the protocol, the individual channels open in the chip to allow sample and detection assay mixing to occur. (Step 3) Sequence specific Cas13-crRNA complexes recognize and bind to RNA which unleashes the collateral cleavage activity of Cas13. Cas13 cleaves the quenched fluorescent reporter in solution. (Step 4) The IFC is loaded into the Fluidigm Biomark HD after the controller protocol finishes. The Biomark, incubating at 37 °C, takes images in the ROX, FAM, and HEX channels every 5 mins for up to 3 h post-reaction initiation to monitor fluorescence from the detection reactions. **b**, Synthetic DNA fragments from each of the 21 human respiratory viruses were serially diluted from 10^3^-10^1^ copies µl^−1^ and added to Q5 amplification master mix containing primer pool 1 or 2. After amplification, separate sample and detection assay reactions were prepared for fluorescence-based readout using CARMEN v.1. CARMEN v.1 values are shown as raw fluorescence (red), FAM signal at 3 h post-reaction initiation. All samples were background subtracted from NTC-det negative control. **c**, Kinetic curves of mCARMEN (blue) from Fig. 1b and CARMEN v.1 (red) from **b** at 0, 60, and 180 mins post-reaction initiation.

CARMEN v.1 (ref. ^40^) is limited by custom instrumentation requirements and labor-intensive protocols, which is why we sought to develop a scalable technology that could be broadly implemented. mCARMEN meets these requirements and eliminates the color-coding and dropletization needs of CARMEN v.1 by using commercially available integrated fluidic circuits (IFCs) on the Fluidigm Biomark for <US$13 per sample (Fluidigm) (Fig. 1a and Supplementary Tables 1 and 2). By leveraging Fluidigm microfluidics, we overcame the need for a custom microscope and chips as well as data analysis expertise, which were required for CARMEN v.1. The Fluidigm IFCs use a specific number of assay combinations: 192 samples by 24 detection assays or 96 samples by 96 detection assays, which are all spatially separated (Supplementary Table 3). After manual IFC loading, the Fluidigm controller moves the samples and detection assays through individual channels on the IFC until they reach the chip reaction chamber, where they are thoroughly mixed. We measured fluorescence on the Fluidigm Biomark with our custom automated protocols that take images of the IFC chip every 5 min for 1–3 h at 37 °C (Extended Data Fig. 1a).

In our first implementation of the mCARMEN platform, we designed a panel to detect 21 clinically relevant human respiratory viruses (Supplementary Table 4). This included all viruses covered by BioFire RP2.1-SARS-CoV-2, four other human-associated coronaviruses and both influenza strains- as well as a few additional illness-inducing viruses^41^. To generate maximally active virus-specific crRNAs and PCR primers to detect the 21 viruses, we applied the assay design method ADAPT (Activity-informed Design with All-inclusive Patrolling of Targets; described in the Methods)^35^. We were able to encompass the full genomic diversity of these viral families by including multiple primers, if needed.

We compared the performance of mCARMEN to CARMEN v.1 for detecting synthetic DNA fragments recapitulating the 21 viral targets and found that mCARMEN had the same (13 viruses) or better (8 viruses) analytical sensitivity compared with CARMEN v.1 (Fig. 1b,c and Extended Data Fig. 1b). Both mCARMEN and CARMEN v.1 had 100% analytical specificity but mCARMEN was 100% sensitive to 10^2^ copies μl^−1^ and 98.4% sensitive to 10^1^ copies μl^−1^ while CARMEN v.1 was only 86% and 77.8% sensitive, respectively. Moreover, the mCARMEN reaction rate was accelerated compared with CARMEN v.1, resulting in faster initial detection and signal saturation of targets (Fig. 1d and Extended Data Fig. 1c). This is likely due to the higher temperature at reaction initiation for mCARMEN (37 °C) than for CARMEN v.1 (25 °C) and the extensive sample detection assay mixing that occurs in the mCARMEN IFC, rather than merged droplets mixing by diffusion in CARMEN v.1.

We then benchmarked the performance of both CARMEN diagnostics against RT–qPCR (CDC 2019-nCoV Kit) and/or unbiased metagenomic NGS on patient specimens. We obtained a set of 6 SARS-CoV-2-positive, 4 SARS-CoV-2-negative and 8 influenza A virus (FLUAV)-positive patient specimens for initial testing. mCARMEN and CARMEN v.1 had 100% concordance with RT–qPCR, NGS and each other (Fig. 1e). We also compared performance using 2 different fluorescent reporters, RNase Alert (Integrated DNA Technologies) and a custom 6-Uracil-FAM (polyU) reporter^31^. We found enhanced sensitivity when using a polyU fluorescent reporter due to the preference of LwaCas13 to cleave at uracils^28,29^ (Supplementary Fig. 1a,b).

Aside from SARS-CoV-2 and influenza viruses, the remaining 19 viruses detectable by mCARMEN lacked a recognized criterion standard clinical diagnostic. Thus, we compared mCARMEN to unbiased metagenomic NGS results for the characterization of 58 prepandemic unknown samples collected from patients with a presumed upper respiratory infection (Fig. 1f, Supplementary Table 5 and Supplementary Fig. 1c,d). Both mCARMEN and NGS detected the same respiratory viruses in 13 specimens (7 FLUAV, 2 HCoV-229E, 1 HCoV-NL63, 1 HCoV-OC43, 1 human metapneumovirus (HMPV) and 1 human rhinovirus (HRV)), neither detected respiratory viruses in 42 specimens and they had differing results for 3 specimens, with 93% overall concordance based on an average of approximately 3 million reads per specimen. Nine of the 13 specimens positive by both methods assembled complete genomes while the remaining 4 assembled partial or no genomes but had >10 reads (2 FLUAV, 1 HMPV, 1 HRV). mCARMEN missed 1 virus-positive specimen detected by NGS, a partial FLUAV genome. We found no sequencing reads spanning the mCARMEN amplicon, suggesting that degradation was responsible for the result. mCARMEN detected virus in 2 specimens (1 FLUAV, 1 HRV) where NGS did not detect any viral reads. While we cannot rule out false positive results, metagenomic sequencing has been shown to have poor sensitivity for low viral copy samples^5,19,42^.

### Streamlining mCARMEN for future clinical use

**Fig. 2:**
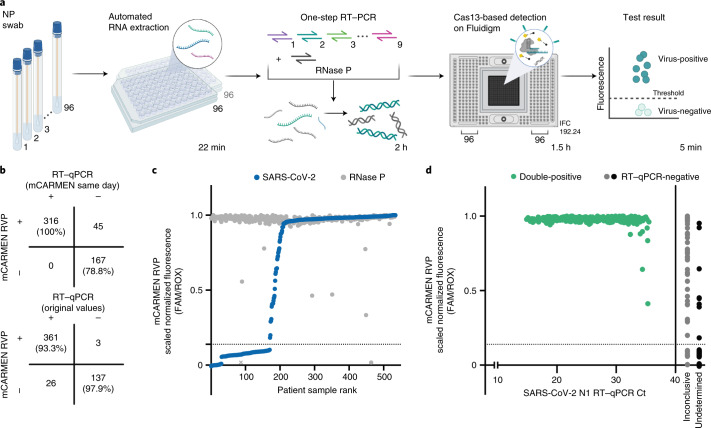
Evaluation of an automated and condensed mCARMEN workflow. **a**, Schematic of the streamlined mCARMEN workflow for testing of 188 patient specimens using a panel of 9 human respiratory viruses, RVP (SARS-CoV-2, HCoV-HKU1, HCoV-OC43, HCoV-NL63, FLUAV, FLUBV, HPIV-3, HRSV, HMPV) and a human internal control (RNase P). **b**, Concordance of RVP and RT–qPCR results. Top, RT–qPCR results were obtained from concurrent testing with mCARMEN. Bottom, RT–qPCR results were obtained from the original testing. **c**, Scaled normalized fluorescence at 1 h post-reaction initiation for 525 NP swabs ranked by increasing SARS-CoV-2 signal (blue); the respective RNase P signal (gray) is also shown. Normalized fluorescence signal (FAM/ROX) scaled from 0 to 1. The NTC-noMg signal was set as 0 and the maximum normalized fluorescence value at 1 h was set as 1. Dashed horizontal line: threshold for RVP positivity, calculated by multiplying the NTC-extract fluorescence value by 1.8; NTC-extract: no template control taken through the entire workflow. Gray X represents a failed sample excluded from concordance calculations and other analyses. **d**, Scatter plot of the scaled normalized fluorescence values from **b** compared to viral Ct values obtained from concurrent testing with the CDC 2019-nCoV Kit. Green: positive SARS-CoV-2 signal detected by both RVP and RT–qPCR; gray: inconclusive RT–qPCR result indicating that one or two of the three technical replicates were undetermined; black: undetermined RT–qPCR result indicating that all three technical replicates were negative for SARS-CoV-2. Dashed horizontal lines: threshold for RVP positivity. Solid vertical line: Ct value of 40 (CDC positivity cutoff).

**Extended Data Fig. 2:**
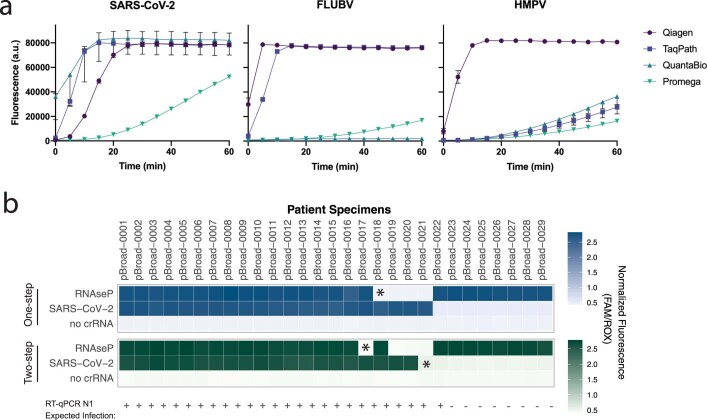
Single-step amplification troubleshooting of synthetic material and patient specimens. **a**, Kinetic curves of the fluorescence for SARS-CoV-2, FLUBV, and HMPV comparing four different single-step amplification kits Qiagen One-Step RT-PCR, TaqPath, QuantaBio, and Promega. Error bars represent the 95% confidence intervals around the median fluorescence values (n = 3). **b**, Single-step amplification by Qiagen One-Step RT-PCR kit (top, blue) compared to two-step amplification by SSIV then Q5 (bottom, green) on 21 SARS-CoV-2 positive and 8 SARS-CoV-2 negative patient specimens. Prior RT-qPCR results shown as expected infection positive (+) or negative (-). All specimens were background subtracted using NTC-amp as a negative control. Asterisk (*) represent differences between one-step and two-step amplification results.

With a drive toward clinical applications, we aimed to optimize the mCARMEN workflow. To do so, we decreased the manual labor and processing time from >8 h to <5 h by implementing automated RNA extraction, using a single-step RNA-to-DNA amplification with 1 primer pool and reducing the duration of detection readout (Fig. 2a and Extended Data Fig. 2a). We then preliminarily evaluated the optimized workflow on 21 SARS-CoV-2-positive and 8 SARS-CoV-2-negative patient specimens and found greater sensitivity over the original two-step amplification method (Extended Data Fig. 2b).

For an end-to-end mCARMEN workflow, we developed software to be used alongside clinical testing to provide patient diagnoses (Supplementary Fig. 2). The software uses the final image at 1 h post-reaction initiation as input, then automatically validates the controls to make 1 of 3 calls—‘detected’, ‘not detected’ or ‘invalid’—for each combination of sample and crRNA.

**Extended Data Fig. 3:**
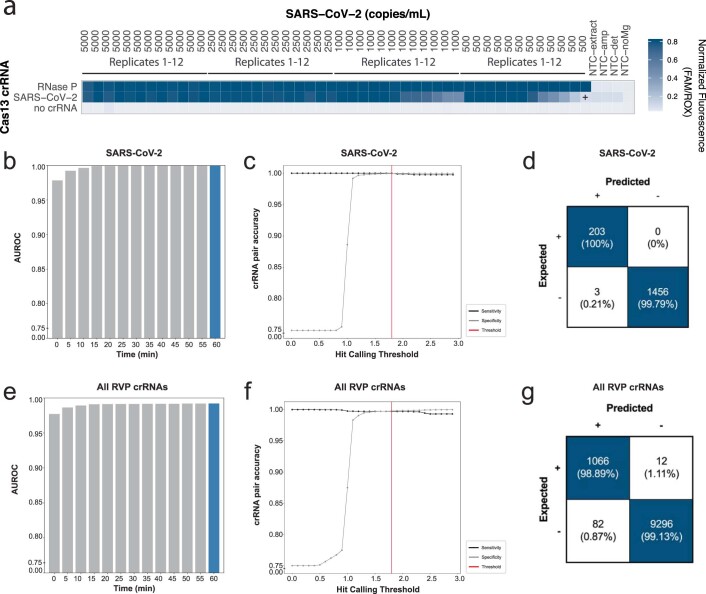
crRNA performance during mCARMEN RVP pre-clinical LOD evaluation. **a**, Fluorescence for 12 replicate testing of SARS-CoV-2 viral seed stock at 5,000, 2,500, 1,000, and 500 copies µl^−1^ spiked into negative patient specimens. All samples were background subtracted using NTC-amp as a negative control. Plus sign (+) represents negative sample calls by RVP results. **b** and **e**, Area under the receiver operating characteristic (AUROC) curve for crRNA detection. Blue bar represents the maximal AUROC time point at 1 hr post-reaction initiation **c** and **f**, Evaluation of sensitivity and specificity of crRNA detection to establish hit calling threshold. Black line: sensitivity/true positive rate; gray line: specificity/true negative rate; red line: hit calling threshold. **d** and **g**, Confusion matrix of expected vs predicted detection. **b-d**, SARS-CoV-2 crRNA detection only. **e-g**, all nine respiratory viruses on RVP.

Lastly, we wanted to condense mCARMEN for focused clinical use and did so by developing a respiratory virus panel (RVP) to detect nine of the most clinically relevant viruses (SARS-CoV-2, HCoV-HKU1, HCoV-OC43, HCoV-NL63, FLUAV, influenza B virus (FLUBV), human parainfluenza virus serotype 3 (HPIV-3), human respiratory syncytial virus (HRSV) and HMPV) and a human internal control, (RNase P). These nine viruses were included in the RVP based on if they heavily circulate in the population and have capacity to cause respiratory virus symptoms, while others were excluded if genomic diversity was difficult to account for concisely, such as HRV^43^. We first conducted range-determining limit of detection (LOD) studies for the nine viruses on the mCARMEN RVP in a research laboratory. The preliminary LOD was within the range of 100–1,000 copies μl^−1^ for SARS-CoV-2, FLUAV, FLUBV, HCoV-HKU1, HCoV-NL63 and HCoV-OC42 and 1,000–20,000 copies μl^−1^ for HPIV-3, HMPV and HRSV (Extended Data Fig. 3a and Supplementary Table 6), with robust performance from the SARS-CoV-2 crRNA and all RVP crRNAs in combination (median areas under the curve (AUCs) of 1 and 0.989, respectively) (Extended Data Fig. 3b–g).

To benchmark mCARMEN RVP performance to comparator assay results, we analyzed 385 SARS-CoV-2-positive and 140 SARS-CoV-2-negative patient specimens and compared these results to both prior and concurrent RT–qPCR evaluation (Fig. 2b). By the time of comparative evaluation, the number of RT–qPCR-positive specimens dropped from 385 to 316, suggesting substantial viral degradation either from extended sample storage or multiple freeze–thaw cycles. Nonetheless, mCARMEN identified all 316 (100% sensitivity) of the concurrent RT–qPCR-positive specimens. We noted that mCARMEN further detected SARS-CoV-2 in 42 specimens that tested positive by prior RT–qPCR but were missed by concurrent RT–qPCR testing, suggesting that mCARMEN is more robust to low viral quantity.

To confirm RT–qPCR sensitivity relative to mCARMEN, we tested the impact of multiple freeze–thaw cycles at several concentrations of SARS-CoV-2 seed stock on assay reproducibility. We found that the freeze–thaw cycles had no impact on mCARMEN sensitivity across all concentrations while RT–qPCR was negatively impacted by freeze–thaw cycles at the lowest concentration, suggesting that the 42 discrepant specimens had low initial viral quantities (Supplementary Fig. 3a–d).

Indeed, if we categorize putative true positives as all specimens that tested positive by prior RT–qPCR as well as present-day RT–qPCR and/or mCARMEN, mCARMEN would have 100% sensitivity compared to 88% for RT–qPCR. mCARMEN also detected SARS-CoV-2 in three specimens that tested negative by both prior and concurrent RT–qPCR (Supplementary Fig. 3e). While we cannot rule out the possibility of false positives, several pieces of evidence suggest they are more likely to be true positives: mCARMEN demonstrated higher sensitivity over concurrent RT–qPCR testing, these specimens were from suspected SARS-CoV-2 cases based on clinical features and mCARMEN did not detect SARS-CoV-2 in any clinical specimens before the pandemic (Fig. 1 and Supplementary Fig. 1).

**Extended Data Fig. 4:**
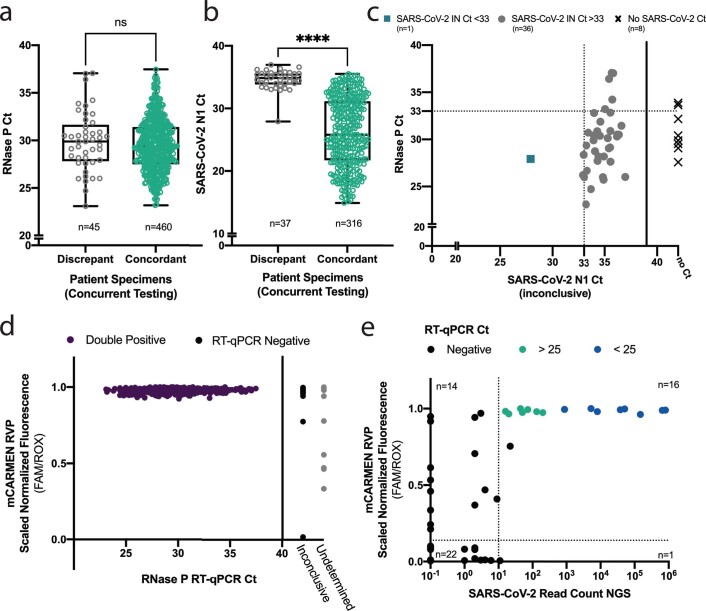
Evaluation of discrepant SARS-CoV-2 clinical specimens tested in an academic setting. **a**, SARS-CoV-2 N1 RT-qPCR inconclusive specimens (n = 45) from Fig. 2b. Average N1 Ct of 1 or 2 replicates compared to average N1 Ct across all concordant specimens. **b**, RNase P RT-qPCR inconclusive specimens from Fig. 2b. compared to RNase P Ct values from all concordant specimens with RNase P Ct <40. **a** and **b**, Box plot with center lines at the medians; box limits indicate the 25^th^ and 75^th^ percentiles; whiskers extend 1.5 times the interquartile range from the 25^th^ to 75^th^ percentiles, all points shown as dots. One-sided t-test was used to measure significance between discrepant and concordant specimens (****, p < 0.0001; ns, not significant). Gray: discrepant specimens; Green: concordant specimens. **c**, Scatter plot of inconclusive specimens from Fig. 2b to compare RNase P Ct to SARS-CoV-2 N1 Ct, if available. Blue: inconclusive N1 Ct <33; Gray: inconclusive N1 Ct >33; X: no N1 Ct. **d**, Scatter plot of RNase P scaled normalized fluorescence from Fig. 1b compared to RNase P Ct values obtained from concurrent RT-qPCR testing of 525 patient specimens. Purple: positive RNase P signal detected by both RVP and RT-qPCR; gray: inconclusive RT-qPCR result indicating one or two of the three technical replicates were undetermined; black: undetermined RT-qPCR result indicating all three technical replicates were negative for RNAse P. **e**, Concordance of RVP scaled normalized fluorescence values and SARS-CoV-2 read counts by unbiased NGS. Dashed horizontal line: threshold for RVP positivity; dashed vertical line: 10 reads mapped to the SARS-CoV-2 genome by NGS, threshold for NGS positivity. Blue: SARS-CoV-2 N1 RT-qPCR Ct < 25; green: Ct > 25; black: negative by RT-qPCR.

We further evaluated the analytical sensitivity of RVP by correlating RVP fluorescence signals to Ct values obtained from concurrent RT–qPCR testing (CDC 2019-nCoV Kit). Of the 316 specimens positive for SARS-CoV-2 by mCARMEN RVP and both RT–qPCR results, 217 had Ct values <30, suggesting moderate-to-high viral genome copies. By RVP, all 217 specimens (100%) reached signal saturation by 1 h post-reaction initiation (Fig. 2c,d and Supplementary Fig. 3f). The remaining 100 specimens had Ct values between 30 and 36 and all but 6 samples (94%) reached signal saturation by 1 h. In total, 98% (311 out of 316) of the specimens reached saturation by 1 h indicating that mCARMEN can rapidly deem viral positivity status for a range of Ct values. Even 17 of the 42 (approximately 40%) RVP-positive specimens, but not concurrently RT–qPCR-positive, reached saturation by 1 h; the slower saturation of the remaining 25 specimens further suggested detection issues caused by low viral genome copy number (Extended Data Fig. 4a–c). We also evaluated RVP fluorescence for detecting an internal control and human housekeeping gene, RNase P. We found that 520 of the 525 (99%) patient specimens reached saturation for RNase P by 1 h (Extended Data Fig. 4d, described in the Methods).

Additionally, we used unbiased metagenomic NGS as a metric to evaluate RVP performance. As controls for NGS, we sequenced a set of true SARS-CoV-2-negative specimens (that is, negative by all three results, RVP and 2× RT–qPCR) (*n* = 16)) and true SARS-CoV-2-positives (*n* = 15) with a range of Ct values (15–34) (Extended Data Fig. 4e and Supplementary Table 5). Fifteen out of the 16 true negatives had no more than 2 reads mapped to the SARS-CoV-2 genome, in line with <10 reads expected for negative specimens, while 1 specimen had 11 reads by NGS (an average of approximately 8.8 million reads per specimen). All true positive specimens had >10 aligned viral reads, ranging from 16 to 802,306 reads, by NGS (100% sensitivity). Only specimens with Ct values <25 (*n* = 8) were able to assemble complete genomes, while specimens with Ct values >25 (*n* = 7) had <200 reads mapped to SARS-CoV-2.

Using NGS, we then evaluated 22 specimens discordant between RVP and RT–qPCR results and 8 specimens for which RVP detected other respiratory viruses. The 22 discordant specimens included 13 positive by RVP and previous testing but concurrently negative by RT–qPCR, 6 positive by previous testing but negative by concurrent RVP and RT–qPCR and 3 positive by RVP but negative by both RT–qPCR results. All but 1 of the 22 (95%) discordant specimens had <10 viral reads by NGS. The single specimen with >10 reads was positive by RVP and prior RT–qPCR but not concurrent testing, yet just 22 reads mapped to SARS-CoV-2. NGS additionally failed to detect other respiratory viruses in the eight RVP-positive specimens. RVP identified 4 SARS-CoV-2 coinfections (2 HCoV-HKU1, 1 HPIV-3 and 1 HRSV) and 4 viruses in SARS-CoV-2-negative specimens (3 FLUAV and 1 HCoV-NL63). Given these specimens also had <10 viral reads aligned by NGS, we can neither validate our results as positive nor rule out the possibility of false negatives by NGS; these samples are likely of low viral quantity suggesting that mCARMEN and RT–qPCR are more sensitive.

### Evaluation of RVP performance in a clinical setting

**Fig. 3:**
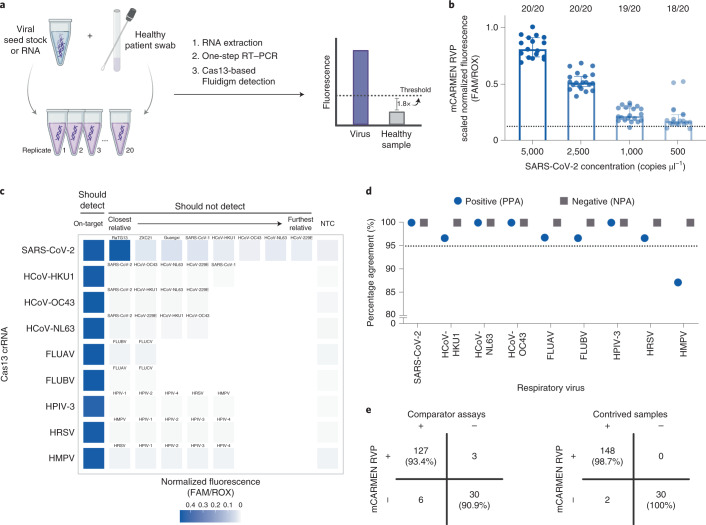
Clinical evaluation of RVP in a CLIA-certified laboratory. **a**, Workflow for LOD studies according to the FDA guidelines for establishing assay sensitivity. **b**, Fluorescence values for SARS-CoV-2 target LOD at the indicated SARS-CoV-2 concentrations; 20 replicates were performed. **c**, Normalized fluorescence signal at 1 h post-reaction initiation for each virus on the RVP using the on-target sequence (should detect), closely related sequences (should not detect) and an NTC (see Supplementary Table 2 for sequence information). Should and should not detect activities were based on ADAPT design predictions (Methods). Closest to further relatives are based on percentage nucleotide homology to the corresponding on-target sequence. **d**, Positive and negative percentage agreement (PPA, NPA, respectively) for each virus on the RVP, calculated based on clinical data in Supplementary Table 5. Dashed line: FDA agreement cutoff for assay performance. **e**, Concordance of the performance of RVP to concurrent comparator assays for 166 retrospective patient specimens tested (left) and 150 contrived samples (right).

**Extended Data Fig. 5:**
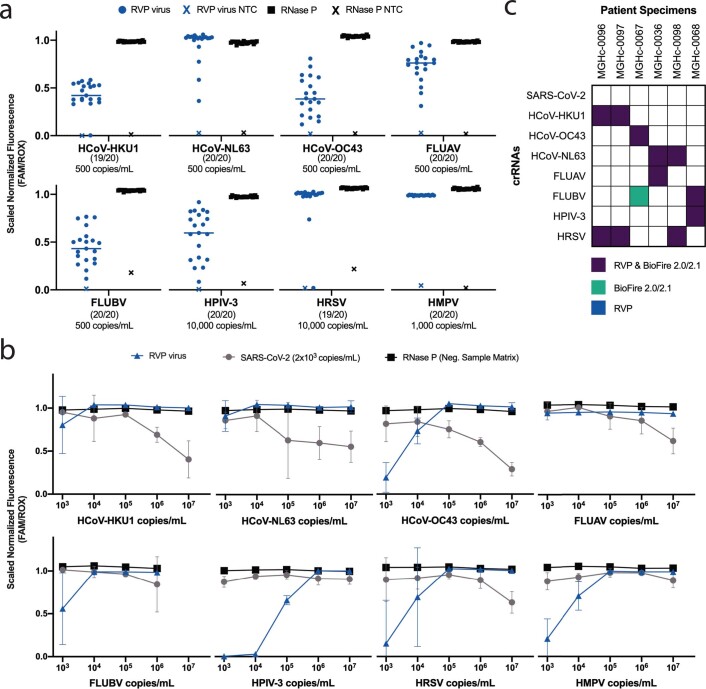
mCARMEN RVP sensitivity evaluation, LOD and co-infections, at MGH. **a**, Fluorescent values at the LOD of all 9 viruses on RVP as established by spiking viral seed stock, isolated RNA extracted from clinical specimens, or synthetic RNA into confirmed negative patient specimen for 20 replicates. NTC, no template control; Viral scaled normalized fluorescence values, circles; RNase P values, squares; NTC-noMg values, ‘X’; Line at median fluorescence. **b**, Scaled normalized fluorescence values at 1 hr post-reaction initiation are shown for SARS-CoV-2 and RNase P, as well as another RVP virus. SARS-CoV-2 viral seed stock was spiked into pooled negative specimen at 2,000 copies µl^−1^ (2x the LOD established in Fig. 3b). Additionally, viral seed stock, isolated RNA extracted from clinical specimens, or synthetic RNA was spiked at 10^7^-10^3^ copies µl^−1^. Error bars represent the 95% confidence intervals around the mean fluorescence values (n = 3). Blue: RVP virus at varying concentrations; light purple: SARS-CoV-2; black: RNase P. **c**, Concordance between RVP and Biofire RP 2.01/2.1 for 6 patient specimens with co-infections visualized as a split heatmap. Purple: RVP and BioFire 2.0/2.1 positive; blue: RVP virus-positive; green: Biofire RP2.0/2.1 virus-positive; white: virus-negative.

We implemented mCARMEN RVP in the Clinical Laboratory Improvement Amendments (CLIA)-certified Clinical Microbiology Laboratory at Massachusetts General Hospital (MGH) to establish assay sensitivity and specificity for clinical validation according to U.S. Food and Drug Administration (FDA) guidelines. We first evaluated the LOD, defined as the lowest concentration yielding positive results for at least 19 of 20 replicates. After recapitulating the 9 viral targets on RVP, we found that the LOD for HCoV-HKU1, HCoV-NL63, HCoV-OC43, FLUAV and FLUBV were 500 copies μl^−1^ while HMPV and SARS-CoV-2 were 1,000 copies μl^−1^ and HPIV-3 and HRSV were 10,000 copies μl^−1^ (Fig. 3a,b, Extended Data Fig. 5a and Supplementary Table 7). The LOD likely varies between viral targets for a few reasons: the crRNAs have varying activity levels on their intended target and differing input materials were used based on sample availability.

After establishing the single-analyte LODs, we asked whether coinfections impacted the sensitivity for each virus detected by RVP. To do so, we added SARS-CoV-2 at a constant, 2× LOD concentration to the remaining 8 viruses on the RVP at varying concentrations at and above their respective LOD (Extended Data Fig. 5b). We observed no loss in our ability to detect SARS-CoV-2. However, we noticed a decrease in signal intensity for the other viruses at lower concentrations, yet only one virus, HPIV-3, had a tenfold higher LOD.

Although we observed no cross-reactivity between RVP panel members in the research setting (Figs. 1 and 2), we followed FDA guidelines to conduct more stringent assay inclusivity and specificity analyses against common respiratory flora and other viral pathogens. In silico analysis revealed that the primers on RVP were >92% inclusive of the known genetic diversity of each viral species, with additional inclusivity coming from crRNA target recognition, for an overall >95% inclusivity (Supplementary Table 8). When examining off-target activity in silico, the FDA defines cross-reactivity as >80% homology between one of the primers or probes to any microorganism. We found no more than 75% homology between the RVP primer and crRNA sequences to other closely related human pathogens (Supplementary Table 9). This implies that off-target detection will rarely, if ever, occur.

**Extended Data Fig. 6:**
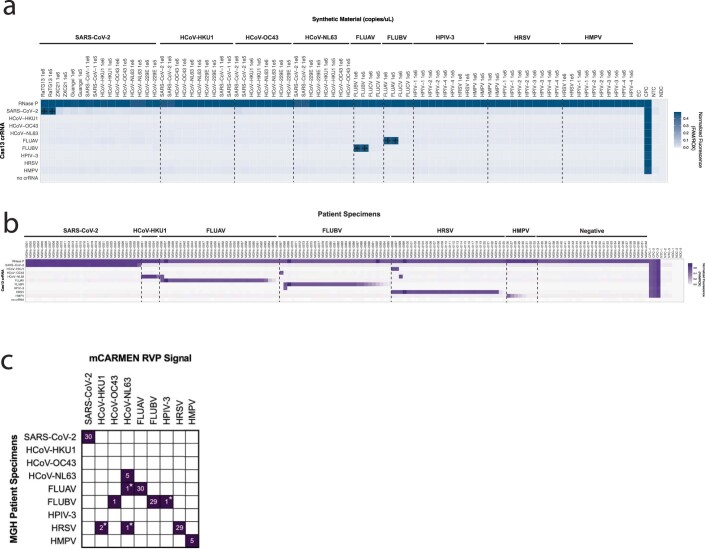
mCARMEN RVP specificity testing at MGH on *in silico* designed synthetic targets and patient specimens. **a**, Fluorescence at 1 hr post-reaction initiation on *in silico* designed synthetic targets spiked into pooled negative patient specimen at 10^6^ copies µl^−1^ for specificity evaluation. Plus sign (+) represents expected cross-reactive signal. Dashed vertical line for visual aid. **b**, Fluorescence at 1 hr post-reaction initiation for 166 patient specimens with known infection status from Fig. 3d–f. Dashed vertical line for visual aid. **c**, Patient specimen summary from **b** comparing RVP and comparator assay results (Cepheid and BioFire 2.0/2.1). Asterisk (*) represents co-infections that were confirmed by BioFire 2.0/2.1.

After in silico analysis, we evaluated RVP specificity experimentally. We computationally designed position-matched synthetic gene fragments from closely related viral species, including both human- and nonhuman-infecting species. When evaluating these gene fragments, only SARS-CoV-2 and bat coronavirus RaTG13 showed cross-reactivity (Fig. 3c and Extended Data Fig. 6). However, this cross-reactivity was expected because the RaTG13 amplicon evaluated shares 100% nucleotide identity with the SARS-CoV-2 amplicon in our assay. We did not observe any cross-reactivity when using viral seed stocks, genomic RNA or synthetic RNA from ATCC or BEI (Supplementary Table 10). Therefore, we found the RVP to have 100% analytical specificity.

Finally, the FDA recommends testing a minimum of 30 known-positive clinical specimens for each pathogen in an assay, as well as 30 negative specimens. Where positive specimens are not available, the FDA allows the creation of contrived samples by spiking viral genomic material at clinically relevant concentrations into a negative specimen. Each virus evaluated must have a minimum of 95% agreement performance, both positive percentage agreement (PPA) and negative percentage agreement (NPA), to clinically approved comparator assays.

**Extended Data Fig. 7:**
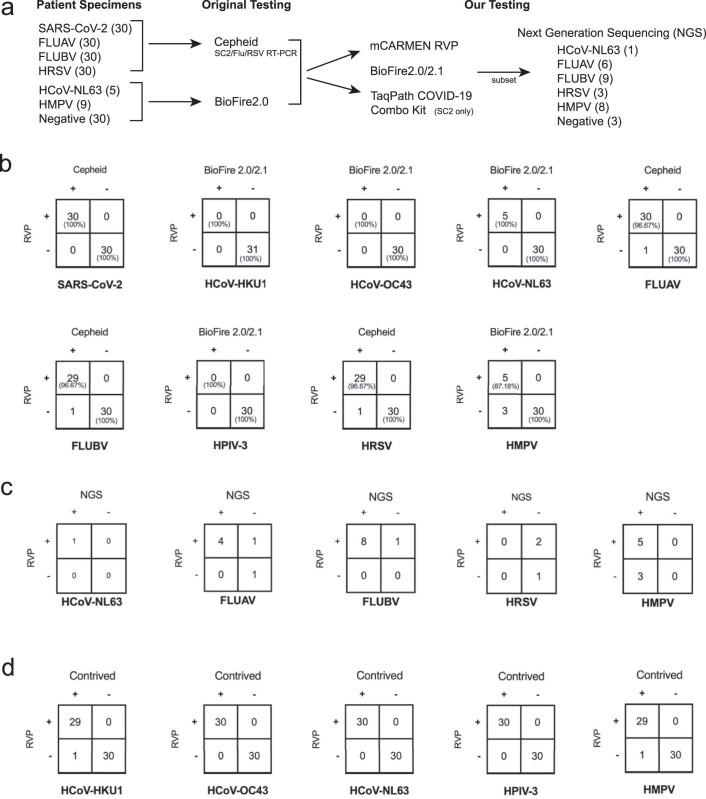
Agreement of mCARMEN RVP patient and contrived sample testing in CLIA-certified laboratory. **a**, Workflow of patient specimen evaluation. Patient specimens were originally tested by either Cepheid and BioFire RP2.0/2.1. We tested these specimens by both RVP and BioFire RP2.0/2.1 or TaqPath COVID-19 Combo Kit then a subset of these specimens were subjected to unbiased NGS. **b**, Concordance between RVP and either Cepheid or BioFire RP2.0/2.1 for 166 patient specimens. Positive and negative percent agreements are shown in parenthesis. **c**, Concordance between RVP and NGS for 30 out of the 166 patient specimens. **d**, Concordance of contrived samples to RVP. Contrived samples were prepared by spiking viral seed stock, genomic RNA, or synthetic RNA into negative patient specimens.

At MGH, archived clinical specimens had been evaluated at the time of collection using one of two comparator assays: Cepheid Xpert Xpress SARS-CoV-2/Flu/RSV multiplexed assay or BioFire RP2.0 multiplexed assay (Extended Data Fig. 7 and Supplementary Table 5). These included 166 specimens with 137 total viral clinical results: 31 FLUAV, 30 SARS-CoV-2, 30 HRSV, 29 FLUBV, 8 HMPV, 5 HCoV-NL63, 1 FLUBV and HCoV-NL63 coinfection, 1 HCoV-HKU1, 1 HCoV-OC43 and 30 clinically negative. Given that these specimens can be degraded by multiple freeze–thaw cycles, we concurrently tested all specimens by BioFire RP2.0 or TaqPath COVID-19 Combo Kit. We also supplemented this evaluation with 30 contrived samples for each of the following viruses for which we did not have enough positive specimens: HCoV-HKU1, HCoV-OC43, HCoV-NL63, HPIV-3 and HMPV (described in the Methods), for a total of 150 contrived samples.

All of the RVP viral targets individually had 100% NPA, and all, except HMPV, had >95% PPA to their respective previous comparator assay result, exceeding the minimum clinical performance set by the FDA (Fig. 3d). Of the 137 previously positive clinical results, mCARMEN correctly detected viral nucleic acids 95% (130 out of 137) of the time. For specimens that were evaluated concurrently, mCARMEN and the comparator assay had 9 discordant results (128 out of 137) with equivalent sensitivity to prior results for all but the HMPV specimens; BioFire did not detect virus in 3 specimens (1 FLUAV, 1 FLUBV and 1 HRSV) and mCARMEN did not detect virus in 6 specimens (1 FLUAV, 1 FLUBV, 1 HRSV and 3 HMPV). Both mCARMEN and BioFire identified 5 specimens with coinfections (HCoV-NL63 in a FLUAV specimen, HPIV-3 in a FLUBV specimen, HCoV-HKU-1 in 2 HRSV specimens and HCoV-NL63 in an HRSV specimen). Together with the original clinically detected coinfection, there were 6 (1.1%) coinfections in our specimen set (Extended Data Fig. 5c). Overall, mCARMEN and BioFire were 99.4% (1,485 out of 1,494 individual tests) concordant (Fig. 3e and Extended Data Fig. 7). For the contrived samples, mCARMEN correctly identified 99% (148 out of 150) (Fig. 3e).

We used unbiased metagenomic NGS to further evaluate the 9 discordant specimens (2 FLUAV, 2 FLUBV, 2 HRSV and 3 HMPV), generating an average of 13 million reads per specimen. Either no viral reads were present by NGS or partial genomes were assembled but the RVP amplicon was missing, making it unlikely for our assay to return a positive result (Extended Data Fig. 7 and Supplementary Table 5). Based on these results and our previous NGS testing, which indicated that NGS was not as sensitive as RVP or the comparator assays, we cannot determine the viral positivity status of these specimens.

### Quantification of viral copies using Cas12 and Cas13 kinetics

Similar to widely used multiplexed approaches, such as BioFire^13^, the original design of CARMEN^40^ did not provide a quantitative assessment of viral genome copies present in a sample. Establishing the total viral quantity in a patient is important for assessing the stage of infection, transmission risk and most effective treatment plan^8,9^. The criterion standard assay for sample quantification, RT–qPCR, leverages the standard curve—serial dilutions of a given target at a known concentration—as a means of using Ct values to approximate viral quantity^44^. We wanted to determine if a similar approach could be applied to mCARMEN.

**Fig. 4:**
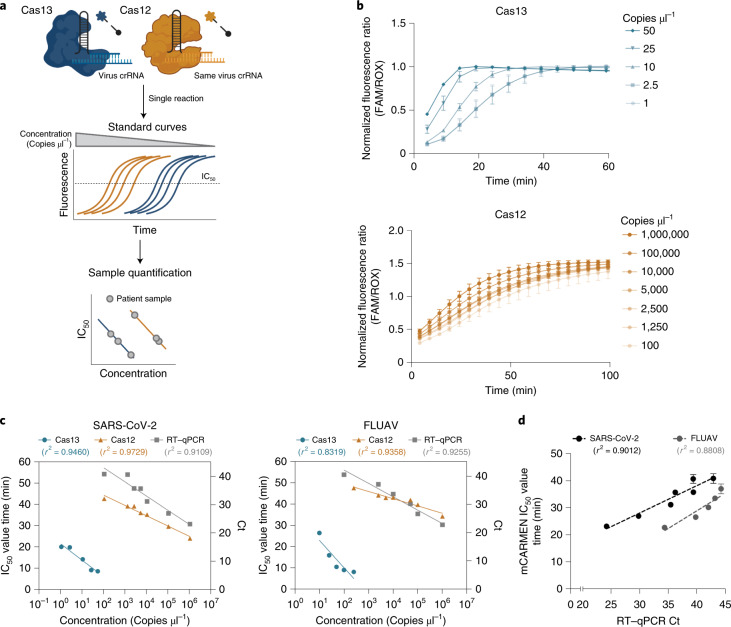
Viral quantification using Cas12 and Cas13 in combination. **a**, Schematic of the procedure for the use of both Cas12 and Cas13 for quantification of viral copy number in samples. Fluorescence was plotted over time to determine the time at which fluorescence signal reaches 50% calculated as the IC_50_ value at each concentration using a sigmoidal 4PL fit. The IC_50_ values were then plotted by concentration to generate a semilog line with an *R*^2^ > 0.8 for Cas12 and Cas13 individually. After line generation, the IC_50_ value of each patient sample was plotted onto these lines to determine viral copies μl^−1^. **b**, Normalized fluorescence ratio of Cas13 crRNA (top) and Cas12 crRNA (bottom) signal over time at varying concentrations of synthetic SARS-CoV-2 Orf1ab RNA. **c**, Plots showing the semilog lines generated by IC_50_ values from the Cas12 and Cas13 crRNA signals and the Ct values from RT–qPCR for more than four target concentrations of SARS-CoV-2 (left) and FLUAV (right) synthetic RNA. Blue: Cas13; orange: Cas12; gray: Ct from RT–qPCR. **d**, Comparison of mCARMEN IC_50_ values to RT–qPCR Ct values using linear regression, with the best line fit shown as a dashed line. Black: SARS-CoV-2; gray: FLUAV.

To make mCARMEN quantitative, we took advantage of the existence of multiple CRISPR–Cas proteins with differing reaction kinetics and enzymatic activities as well as the three fluorescent channels detected by the Fluidigm Biomark (Fig. 4a). We incorporated DNA-targeting CRISPR–Cas12 into the Cas13 reaction and used protein-specific reporters in different fluorescent channels, HEX and FAM, respectively to maximize our multiplexing capabilities. To capture reaction kinetics, images of the IFC chip were taken every 5 min for 3 h to generate sigmoidal curves from the fluorescent signals over time. When considering enzymatic activities, Cas13 has enhanced sensitivity compared to Cas12 since the process of in vitro transcribing the double-stranded DNA (dsDNA) sample input for Cas13 detection results in an increased starting concentration. Thus, we used Cas12 to capture the kinetic curves of higher copy material on the standard curve and Cas13 to capture lower copy material.

**Extended Data Fig. 8:**
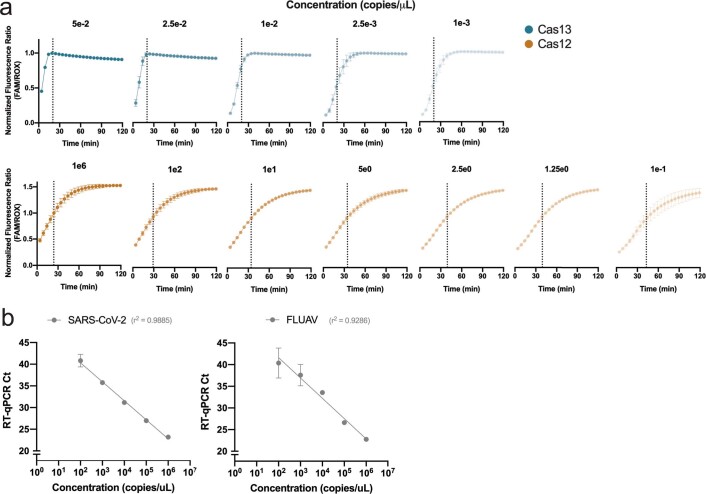
Sample quantification with combined Cas12 and Cas13 detection. **a**, Kinetic curves of SARS-CoV-2 detection by both Cas13 (blue) and Cas12 (orange) for 5 or 7 concentrations, respectively, up to 2 h post-reaction initiation. Dashed lines represent the time at which signal reaches 50% (IC_50_ infection point) for each concentration of target. Error bars represent the 95% confidence intervals around the median fluorescence values (n = 2). **b**, Standard curves for SARS-CoV-2 and FLUAV based on RT-qPCR Ct values. Data related to Fig. 4c, now showing a standard 10-fold dilution series. Error bars represent the 95% confidence intervals around the mean RT-qPCR Ct (n = 6).

We integrated our quantification efforts into the RVP because this assay was evaluated extensively in both research and clinical settings. We manually designed Cas12 crRNAs in the same region of the viral genome that the RVP Cas13 crRNAs target for a two-step standard curve generation on the same target amplicon for Cas12 and Cas13 proteins individually. The first step requires plotting the fluorescence for a range of concentrations (Cas12: 10^7^–10^3^ copies μl^−1^; Cas13: 10^3^–10^0^ copies μl^−1^) at each time point to calculate the time at which the fluorescence intensity concentration reaches 50% (IC50) through a sigmoidal, four-parameter logistic (4PL) curve, *R*^2^ > 0.9 (Fig. 4b and Extended Data Fig. 8). In some cases, we could not determine the IC_50_ value because signal saturation occurred too quickly or not at all; therefore, that concentration was excluded from the analysis. In the second step, we plotted the IC_50_ values onto a semilog line, where concentration is logarithmic and time is linear, to generate the standard curves (Fig. 4c). We compared these results to a standard curve generated from RT–qPCR using the same serial dilutions and found a linear relationship between SARS-CoV-2 and FLUAV IC_50_ values to Ct values (*R*^2^ = 0.901 and 0.881, respectively) (Fig. 4d). Taken together, these results suggest that by using Cas12 and Cas13 in combination, we could extrapolate viral quantification—spanning a 10^0^–10^6^ range of target concentrations—from patient specimens with performance similar to RT–qPCR.

### Allelic discrimination distinguishes between SARS-CoV-2 variant lineages

Since current clinical diagnostics are not well positioned to identify mutations—single-nucleotide polymorphisms (SNPs), insertions or deletions—carried in SARS-CoV-2 variant lineages^6,17,18^, we wanted to develop a single platform with both diagnostic and surveillance capabilities for comprehensive detection of 26 SARS-CoV-2 Spike gene mutations. We selected these 26 mutations to distinguish between or detect mutations shared among the Alpha, Beta, Gamma, Delta and Epsilon variant lineages (Supplementary Table 11; B.1.1.7, B.1.351, P.1, B.1.617.2 and B.1.427/9 using the Pango nomenclature system, respectively; World Health Organization (WHO) Tracking SARS-CoV-2 variants) and then used a generative sequence design algorithm (Mantena, S. et al., manuscript in preparation) to produce crRNAs for allelic discrimination.

**Fig. 5:**
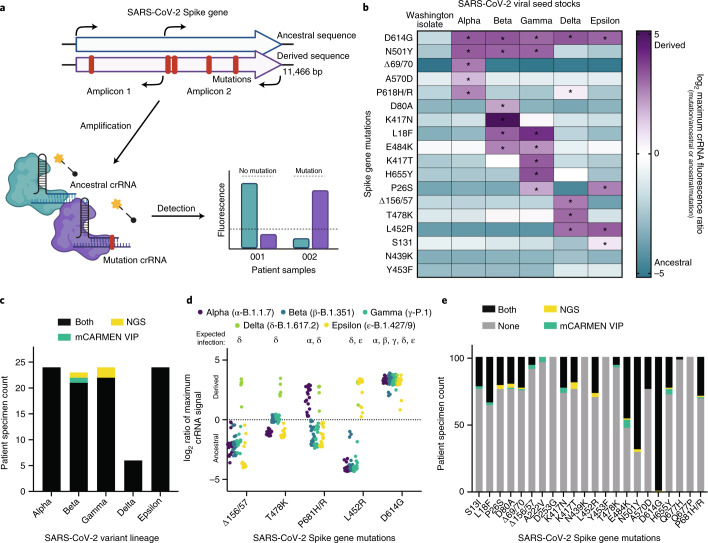
SARS-CoV-2 variant identification using SNP-determining Cas13 crRNA combinations. **a**, Schematic of the procedure for the mCARMEN VIP. The entire SARS-CoV-2 Spike gene was amplified to detect the presence of a mutation by differentiating between the ancestral, no mutation, sequence or the derived, mutation-containing sequence with highly specific Cas13 crRNAs. **b**, SARS-CoV-2 viral seed stocks from ancestral (Washington isolate), Alpha, Beta, Gamma, Delta or Epsilon variant lineages (10^6^ copies μl^−1^) were amplified by 2 primer pairs and tested for the presence or absence of Spike gene mutations. Data are shown as the log_2_ of the maximum crRNA fluorescence ratio at any time point up to 3 h post-reaction initiation. log_2_ fluorescence ratios were calculated by (−1)*ancestral/mutation or mutation/ancestral representing either the presence of the ancestral (blue) or derived sequence (purple), respectively. The single asterisk indicates that the particular mutation was confirmed by NGS. **c**, Comparison of the performance of and NGS on 101 SARS-CoV-2-positive variant patient specimens, based on the final variant call as assessed by unique combinations of mutations (see the Methods for details). Black: variant correctly identified by both VIP and NGS; yellow: NGS only; green: VIP only. **d**, Plot showing the log_2_ maximum crRNA fluorescence ratio of mutation/ancestral (positive, derived) or ancestral/mutation (negative, ancestral) at any time point up to 3 h post-reaction initiation for 101 variant patient specimens tested for various SNPs by VIP. Patient specimens are classified as Alpha (purple), Beta (blue), Gamma (teal), Delta (green) or Epsilon (yellow) based on a combination of mutations expected for that variant lineage. **e**, Analysis of how VIP compared to NGS for the 101 variant patient specimens. Black: mutation correctly identified by both VIP and NGS; yellow: NGS only; green: VIP only; gray: ancestral for VIP and NGS.

With the continuous emergence of mutations that can lead to increased transmissibility or enhanced virulence, we also wanted to greatly streamline assay generation for each new SARS-CoV-2 mutation or variant. Thus, we developed an easily adaptable method to track these changes that we called the mCARMEN variant identification panel (VIP). VIP has two nonoverlapping primer pair sets within conserved regions of the Spike gene to amplify the full-length sequence for use with any crRNA pair. These 26 crRNA pairs, individually or in combination, allowed us to track existing variants and identify emerging variants (Fig. 5a). Initially, we tested over 60 combinations of crRNAs on unamplified synthetic material to identify the crRNA pairs with the largest fluorescence ratio of expected divided by unexpected signal for each mutation (Supplementary Fig. 4).

**Extended Data Fig. 9:**
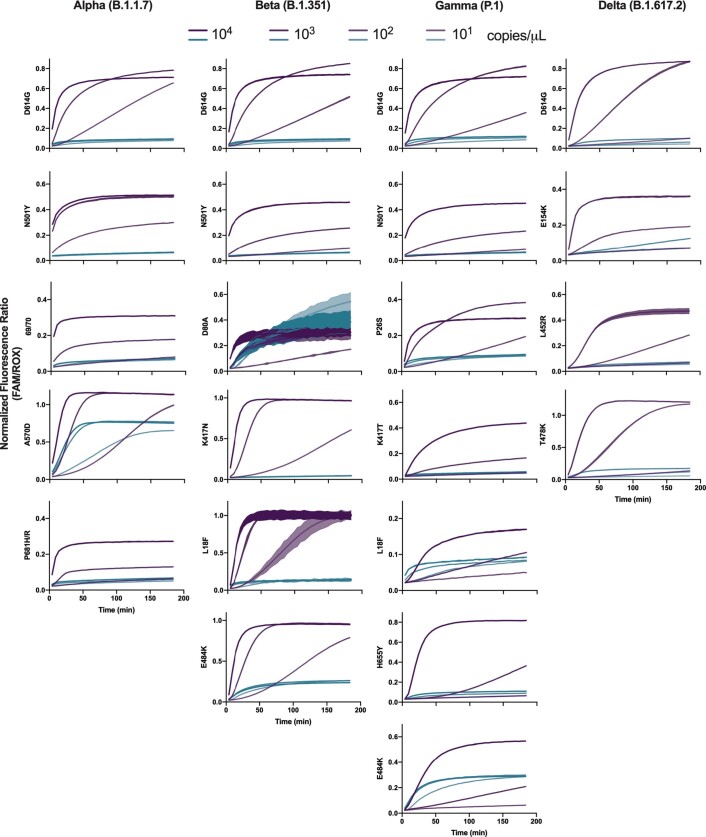
Mutation detection for Alpha, Beta, Gamma, and Delta SARS-CoV-2 variant lineages. Kinetics curves of 4-7 SNPs that make up four different variant lineage seed stocks being evaluated. Data shown as normalized fluorescence every 5 mins for 3-4 concentrations of viral seed stock. WA, Alpha, Beta, Gamma: 10^4^-10^2^ copies µl^−1^, and Delta: 10^3^-10^1^ copies µl^−1^. Purple: variant lineage; Blue: ancestral seed stock; colored from high-to-low concentration. Shaded regions represent 95% confidence intervals around the median fluorescence values and lines are at the median fluorescence.

We validated the flexible VIP method by testing RNA extracted from SARS-CoV-2 viral seed stocks, for the ancestral (Washington isolate: USA-WA1; ATCC) lineage and the Alpha, Beta, Gamma, Delta and Epsilon lineages (Fig. 5b and Extended Data Fig. 9). As expected, the Washington SARS-CoV-2 viral seed stock isolate showed ancestral signals for all mutations tested. Alpha, Beta, Gamma, Delta and Epsilon had expected signals for every mutation confirmed by NGS (Supplementary Table 11; description in the Methods). Although each crRNA had different kinetics owing to varying hit-calling thresholds, we almost always observed a higher expected signal above the unexpected signal, which is important in the prevention of false positive results (Supplementary Fig. 5).

For clinical relevance, we developed an automated variant calling procedure that evaluates the mutation-specific signal in SARS-CoV-2-positive patient specimens and returns a variant lineage result (Supplementary Fig. 6a; described in the Methods). For some mutations at the same or similar genomic position, we observed cross-reactive signals that we overcame by comparing the maximum fluorescence ratios between those mutations and assigning the positive call to the higher of the two (Supplementary Fig. 6b).

We applied VIP and the analysis pipeline to identify the variant lineage in 101 known SARS-CoV-2-positive patient specimens: 24 Alpha, 23 Beta, 24 Gamma, 6 Delta and 24 Epsilon. Of the 101 specimens with NGS results, all but 3 (97%) specimens (1 Beta and 2 Gammas) were given the correct variant lineage identification (Fig. 5c,d, Supplementary Fig. 7a and Supplementary Table 5). The Beta specimen had signal for a Beta-specific SNP, K417N, but also had signal for Δ156/57, a Delta-specific SNP. The Gamma specimens had no unique signals and shared signals for mutations overlapping with the Beta lineage resulting in a ‘variant not identified’ call.

Focusing on the results for the individual mutations themselves, we found only one mutation, E484K, had more than five specimens that differed in their results between NGS and VIP (Fig. 5e). The 7 E484K discrepancies are attributed to the cross-reactive signals between E484K and T478K; thus, new crRNA designs are likely needed to optimally differentiate these signals (Supplementary Figs. 6b and 7b,c). Altogether, we found that VIP had 97.7% concordance to NGS at allelic discrimination.

### VIP identifies Omicron at local and statewide levels

In November 2021, the SARS-CoV-2 variant lineage Omicron (BA.1) was first identified by NGS in South Africa and was quickly associated with a rapid increase in case counts (WHO Coronavirus (COVID-19) Dashboard)^45,46^. By December, Omicron was detected in the USA and has since driven the recent global COVID-19 wave^47^. However, detecting and tracking Omicron has been challenging, with NGS results lagging behind by 7–14 d from collection date. Although S gene target failure (SGTF) by the TaqPath COVID-19 Combo Kit (Thermo Fisher Scientific) can be associated with Omicron, the failure is not specific to Omicron^47,48^. The swift emergence of Omicron has revealed a need for a nucleic acid-based diagnostic with turnaround times similar to RT–qPCR but with mutation-specific information that only NGS currently provides. mCARMEN is uniquely poised to fulfill this need by providing results the same day approximately one week before NGS.

**Fig. 6:**
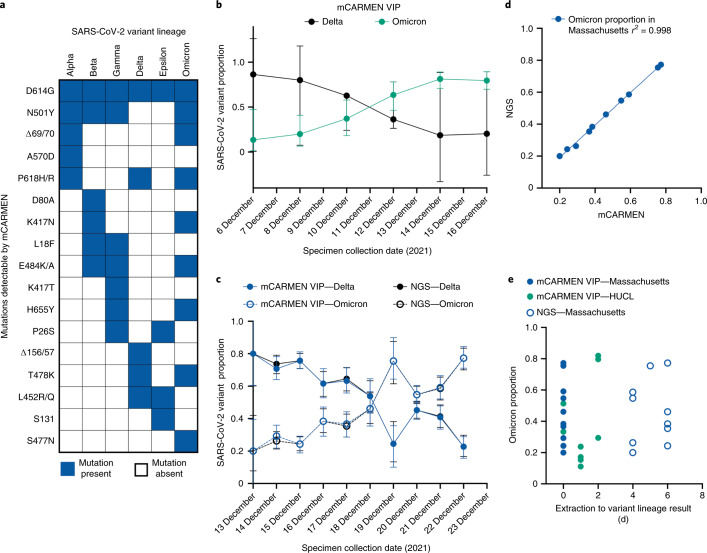
Rapid and specific identification of the Omicron variant using the mCARMEN VIP. **a**, Expected mutations across six SARS-CoV-2 variant lineages (Alpha, Beta, Gamma, Delta, Epsilon, Omicron) detectable by VIP. The blue boxes represent the presence of a mutation; the white boxes represent the absence of a mutation. **b**, Proportion of Omicron and Delta variant lineages as assessed by VIP in specimens (*n* = 430) collected on 6–16 December at HUCL. Green: Omicron; black: Delta. The error bars represent binomial sampling 95% confidence intervals (CIs). **c**, Proportion of Omicron and Delta variant lineages as assessed by VIP and NGS in specimens (*n* = 1,557) collected on 13–22 December from throughout the state of Massachusetts. Blue: Omicron; black: Delta; closed circles: mCARMEN; open circles: NGS. The error bars represent binomial sampling 95% CIs. **d**, Scatter plot of the proportion of Omicron based on the VIP and NGS variant lineage results. The linear regression line fit is shown as a blue line; *R*^2^ = 0.998. **e**, Comparison of the time delay from specimen extraction to determination of the variant lineage for VIP and NGS. The data represent the proportion of Omicron from specimens collected at HUCL and within the state of Massachusetts. Blue closed circles: VIP Massachusetts specimens from **c**; blue open circles: NGS Massachusetts specimens from **c**; green: VIP HUCL specimens from **b**.

**Extended Data Fig. 10:**
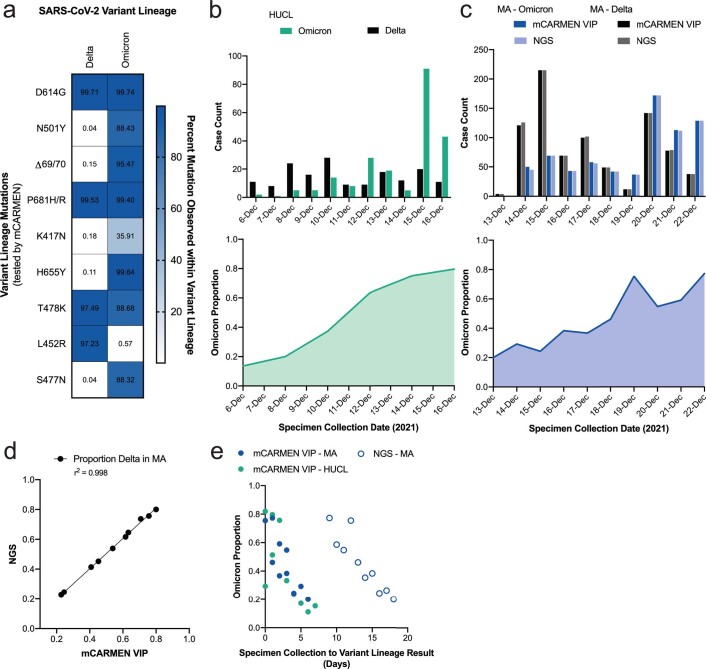
Specimens evaluated for the presence of Omicron by VIP. **a**, Heatmap colored by the percent of available variant genomes on GISAID with a particular variant mutation broken down by variant lineage. **b**, Delta and Omicron case counts and proportion of Omicron by mCARMEN VIP from specimens collected at the Harvard University CLIA Laboratory (HUCL) from December 6-16 2021, related to Fig. 6b (n = 430). Green: Omicron; Black: Delta. **c**, Delta and Omicron case counts between VIP and NGS results with the proportion of Omicron by VIP below (n = 1,557). Specimens collected at the Broad Institute from the state of Massachusetts. Blue: Omicron; Black: Delta. **d**, Scatter plot of the proportion of Delta based on the VIP and NGS variant lineage results from c and Fig. 6c. Linear regression line fit shown as black line; R^2^ = 0.998. **e**, Time delay from specimen collection to variant lineage result between VIP and NGS. Data represents the proportion of Omicron from specimens collected at HUCL and within the state of Massachusetts. Blue closed circles: VIP MA specimens from c; Blue open circles: NGS MA specimens from c; Green: VIP HUCL specimens from b.

At the time of Omicron emergence, mCARMEN VIP could already uniquely differentiate it from the other SARS-CoV-2 variants by specifically detecting nine Omicron-tagging mutations among our variant panel (Fig. 6a). The unique combination of these Spike gene mutations allows for the specificity required to make the proper variant lineage call. In particular, just the combination of S477N and N501Y covers 98.6% of Omicron sequences in GISAID and is 99.9% specific to Omicron (Extended Data Fig. 10a). We rapidly applied VIP to 430 specimens collected at Harvard University CLIA Laboratory (HUCL) from 6 to 16 December and found that the rate of Omicron increased from 15% to 80% in 10 d, overtaking the previously predominant variant, Delta (Fig. 6b and Extended Data Fig. 10b).

Based on the public health importance of this data, the Massachusetts Department of Public Health (MADPH) requested our support for Omicron surveillance across the state. Using the mCARMEN VIP, we tested 1,557 specimens collected across the state of Massachusetts for the presence of Delta or Omicron from 13 to 22 December 2021. The rate of Omicron increased from approximately 20% to 77% across Massachusetts in 10 d (Fig. 6c and Extended Data Fig. 10c). In partnership with MADPH and the Broad Genomics Platform, we were able to confirm the mCARMEN variant lineage results with lineage results determined by NGS and found 99.5% (1,549 out of 1,557) concordance between mCARMEN and NGS (Fig. 6d and Extended Data Fig. 10d). Of the eight discordant samples, seven had low signal for all mutations evaluated by VIP, suggesting low viral quantity. The remaining discordant specimen had clear signal for several Omicron-specific mutations; yet, by NGS, it had Delta signatures, which would suggest likely contamination or sample swap in one of the two tests. In all, the mCARMEN VIP was applied in real-time to a local Omicron outbreak and a statewide Omicron wave with near-perfect concordance to NGS, by providing results the same or following day while NGS lagged behind by approximately 4–7 d (Fig. 6e).

## Discussion

In this study, we report mCARMEN, a high-throughput, multiplexed and microfluidic diagnostic and surveillance platform with panels for respiratory viruses and SARS-CoV-2 variants that can be parallelized to test 300–550 patient specimens in an 8-h working day. To make mCARMEN a clinically relevant technology, we built on CARMEN v.1 (ref. ^40^) by streamlining the workflow and incorporating commercially available Fluidigm instrumentation. We validated mCARMEN on 2,881 patient specimens to detect 9–21 human respiratory viruses (RVP) or SARS-CoV-2 variant mutations (VIP) with high concordance to comparator assays that passed the FDA’s performance criteria for all but 1 virus. Notably, when testing previously positive clinical specimens, we found that a substantial proportion were not positive by concurrent testing but were positive by mCARMEN. This suggests sample degradation issues—a known problem when detecting RNA viruses in clinical specimens^42,49^—that mCARMEN is more robust at handling than RT–qPCR or NGS. Although we cannot rule out false positives, we did not detect SARS-CoV-2 in specimens before the pandemic and we had 100% concordance with true virus-negative specimens.

To enhance mCARMEN’s clinical diagnostic relevance and meld it with surveillance technology requirements, we further maximized its multiplexing capabilities by discriminating between mutations for variant lineage classification in patient specimens. Currently, variant lineage classification is only evaluated by NGS, which is costly and relies on specialized expertise found outside the clinic^17,19^. VIP gives similarly rich information about key SARS-CoV-2 mutations at 5–10 times cheaper in cost, per sample, than NGS and is far more comprehensive than current nucleic acid-based diagnostics. Importantly, since we routinely design guides to preemptively identify mutations of interest in the Spike gene in preparation for emerging variants, VIP was poised to differentiate Omicron immediately. VIP allowed us to identify the rapid emergence of Omicron in Massachusetts approximately 8 d before NGS and provided us with specificity, unlike the widely used SGTF of RT–qPCR. Given the number of mutations detected by VIP, we expect to observe distinct mutation signatures between variant lineages that will allow us to differentiate these and future VOCs from each other without assay redesign.

We also adapted mCARMEN for dual Cas12 and Cas13 detection by capitalizing on the differing protein kinetics. A few groups have studied Cas12 and Cas13 reaction kinetics to inform assay quantification^50,51^ but the range of quantifiable concentrations has been limited due to reaction saturation. We expanded the quantifiable concentration range to 5–6 orders of magnitude, which is similar to RT–qPCR. These mCARMEN applications have the potential to provide a more holistic diagnosis to the patient but validation on patient samples is needed.

We rapidly developed mCARMEN for use in the COVID-19 pandemic but faced challenges during the clinical validation and approval process needed for a large-scale rollout. Specifically, it was difficult to obtain at least 30 previously confirmed clinical specimens for each virus on RVP with enough material available for extensive concurrent testing, while also facing specimen degradation issues that inevitably occur over time. Although our findings indicate that mCARMEN’s performance exceeds the FDA’s requirements for emergency use authorization (EUA), such authorization has not yet been granted.

Further work will be required to bring mCARMEN fully to the clinic, such as obtaining FDA approval, integrating RVP and VIP into a single panel, decreasing the amount of manual labor and easing Fluidigm equipment constraints. Nonetheless, we have taken substantial steps to streamline the assay workflow while enhancing sensitivity without sacrificing specificity. By combining high-throughput, multiplexed pathogen testing with variant tracking, the mCARMEN platform is highly scalable and amenable to clinical laboratory settings for the detection of respiratory pathogens and variants. This technology also has the potential to test for other types of infectious diseases^52^ and can be used on other sample types^40,53^ to achieve even more comprehensive diagnostic and surveillance capabilities.

## Methods

### Patient samples and ethics statement

Use of clinical excess of human specimens from patients with SARS-CoV-2 from the Broad Institute’s Genomics Platform CLIA Laboratory was approved by the MIT institutional review board (IRB) protocol no. 1612793224. Additional SARS-CoV-2 samples were collected from consented individuals under Harvard Longwood Campus IRB no. 20-1877 and covered by an exempt determination (EX-7295) at the Broad Institute. Other human-derived samples from patients with SARS-CoV-2 were collected by the CDC and determined to be nonhuman participant research; the Broad Office of Research Subject Protections determined these samples to be exempt (EX-7209). Human specimens from patients with SARS-CoV-2, HCoV-HKU1, HCoV-NL63, FLUAV, FLUBV, HRSV and HMPV were obtained under a waiver of consent from the Mass General Brigham IRB protocol no. 2019P003305. Researchers at Princeton were determined to be conducting not-engaged human participant research by the Princeton University IRB.

We gratefully acknowledge the personnel at Rhode Island Department of Public Health for the samples they provided, in particular: E. King, Associate Director of Health, and R. C. Huard, Chief Clinical Laboratory Scientist, both at the Division of State Laboratories and Medical Examiner at Rhode Island Department of Health.

### General mCARMEN procedures

A detailed description of running the mCARMEN RVP as a standard operating procedure can be found in Supplementary Note 1.

### Preparation and handling of synthetic materials

crRNAs were synthesized by Integrated DNA Technologies, resuspended in nuclease-free water to 100 μM and further diluted for input into the detection reaction. Primer sequences were ordered from Eton or Integrated DNA Technologies, resuspended in nuclease-free water to 100 μM and further combined at varying concentrations for pooled amplification.

### Preparation of IVT material

DNA targets were ordered from Integrated DNA Technologies and in vitro-transcribed (IVT) using the HiScribe T7 High Yield RNA Synthesis Kit (New England Biolabs). Transcriptions were performed according to the manufacturer’s recommendations with a reaction volume of 20 μl that was incubated overnight at 37 °C. The transcribed RNA products were purified using RNAClean XP beads (Beckman Coulter) and quantified using NanoDrop One (Thermo Fisher Scientific). Depending on the experiment, the RNA was serially diluted from 10^11^ down to 10^−^^3^ copies μl^−1^ and used as input into the amplification reaction.

### Manual or automated extraction

RNA was manually extracted from input material using the QIAamp Viral RNA Mini Kit (QIAGEN) according to the manufacturer’s instructions. RNA was extracted from 140 μl of input material with carrier RNA and samples were eluted in 60 μl of nuclease-free water and stored at −80 °C until use. RNA was automatically extracted using the MagMAX DNA Multi-Sample Ultra 2.0 Kit on a KingFisher Flex Magnetic Particle Processor with 96 Deep Well Head (Thermo Fisher Scientific). RNA was extracted from 200 µl of input material and was run according to the ‘Extract RNA-automated method (200-μl sample input volume)’ protocol in the TaqPath COVID-19 Combo Kit Protocol, on pages 21–24. The MVP_2Wash_200_Flex protocol was used. Samples were eluted in 50 μl of elution solution and either directly added to the amplification reaction or stored at −80 °C until use.

### QIAGEN or SuperScript IV amplification

We followed the CARMEN v.1 platform for two-step reverse transcription amplification then transitioned to a single-step amplification reaction after the experiments depicted in Fig. 1. We used the QIAGEN OneStep RT–PCR Mix for Figs. 2, 3 and 5 and the Invitrogen SuperScript IV One-Step RT–PCR System for Fig. 4. For the QIAGEN OneStep RT–PCR, a total reaction volume of 50 μl was used with some modifications to the manufacturer’s recommended reagent volumes, specifically a 1.25× final concentration of OneStep RT–PCR buffer, 2× more QIAGEN enzyme mix and 20% RNA input. Final concentrations for all viral primers were 300 and 100 nM for the RNase P primers. The following thermal cycling conditions were used: (1) reverse transcription at 50 °C for 30 min; (2) initial PCR activation at 95 °C for 15 min; and (3) 40 cycles at 94 °C for 30 s, 58 °C for 30 s and 72 °C for 30 s. For the Invitrogen SuperScript IV One-Step RT–PCR, a total reaction volume of 25 μl with 20% RNA input and final primer concentrations at 1 μM were used. The following thermal cycling conditions were used: (1) reverse transcription at 50 °C for 10 min; (2) initial PCR activation at 98 °C for 2 min; (3) 35 cycles at 98 °C for 10 s, 60 °C for 10 s and 72 °C for 1 min 30 s; and (4) final extension at 72 °C for 5 min. See Supplementary Table 4 for information on the primer sequences used in each mCARMEN panel.

### Fluidigm detection

The Cas13 detection reactions were made into two separate mixes—assay mix and sample mix—for loading onto a microfluidic IFC (depending on the experiment, either gene expression or genotyping IFCs were used in either a 96.96 or 192.24 format) (Fluidigm).

#### Assay mix

The assay mix contained LwaCas13a (GenScript) and on occasion LbaCas12a (New England Biolabs). Concentration varied with experiment: 1× Assay Loading Reagent (Fluidigm), 69U T7 RNA Polymerase mix (Lucigen) and crRNA concentration varied with experiment for a total volume of 16 μl per reaction. See below for details pertaining to each mCARMEN panel.

#### Sample mix

The sample mix contained 25.2 U RNase Inhibitor (New England Biolabs), 1× ROX reference dye (Invitrogen), 1× gene expression sample loading reagent (Fluidigm), 1 mM ATP, 1 mM GTP, 1 mM UTP, 1 mM CTP, 9 mM MgCl_2_ in a nuclease assay buffer (40 mM Tris-HCl, pH 7.5, 1 mM dithiothreitol). Either a 500-nM quenched synthetic fluorescent RNA reporter (FAM/rUrUrUrUrUrUrU/3IABkFQ/ or VIC/rTrTrArTrTrArTrT/3IABkFQ/; Integrated DNA Technologies) or RNaseAlert v2 (Invitrogen) was used for a total volume of 12.6 μl. See below for details on each mCARMEN panel.

#### IFC loading and run

Syringe, actuation fluid, pressure fluid (Fluidigm) and 4 μl of assay or sample mixture were loaded into their respective locations on a microfluidic IFC (depending on the experiment, either gene expression or genotyping IFCs were used in either a 96.96 or 192.24 format) and were run according to the manufacturer’s instructions. The IFC was loaded onto the IFC Controller RX or Juno (Fluidigm) where the ‘Load Mix’ script was run. After proper IFC loading, images over either a 1- or 3-h period were collected using a custom protocol on Fluidigm’s EP1 or Biomark HD.

### Fluidigm data analysis

We plotted reference-normalized, background-subtracted fluorescence for guide-target pairs. For a guide-target pair (at a given time point, t, and target concentration), we first computed the reference-normalized value as (median(P_t_ − P_0_) / (R_t_ − R_0_)) where P_t_ is the guide signal (FAM) at the time point, P_0_ is its background measurement before the reaction, R_t_ is the reference signal (ROX) at the time point, R_0_ is its background measurement and the median is taken across replicates. We performed the same calculation for the NTC of the guide, providing a background fluorescence value for the guide at t. (When there were multiple technical replicates of such controls, we took the mean value across them.) The reference-normalized, background-subtracted fluorescence for a guide-target pair is the difference between these two values.

### 21 respiratory viruses

#### Design

The oligonucleotide primers and crRNA guides were designed to detect the conserved regions of the following respiratory viruses: SARS-CoV-2, HCoV-229E, HCoV-HKU1, HCoV-NL63, HCoV-OC43, FLUAV, FLUBV, HMPV, HRSV, HPIV-1, 2, 3, 4, adenovirus, HEV-A, B, C, D, SARS-CoV, Middle East respiratory syndrome (MERS)-CoV and HRV. More specifically, complete genomes for all viruses on the panel were downloaded from the National Center for Biotechnology Information (NCBI) and aligned using MAFFT^54^. For viral species with fewer than 1,000 sequences, the MAFFT ‘FFT-NS-ix1000’ algorithm was used. For viral species with >1,000 sequences, the MAFFT ‘FFT-NS-1’ algorithm was used. These aligned sequences were then fed into ADAPT for crRNA design with high coverage using the ‘minimize guides’ objective (>90% of sequences detected). Once highly conserved regions of the viral genome were selected with ADAPT for optimal guide design, primers were manually designed to amplify a 100–250 base pair (bp) target region with the crRNA predicted to bind in the middle of the fragment. ADAPT’s constraints on primer specificity were relaxed; in some cases, multiple primers were needed to encompass the full genomic diversity of a particular virus species. For optimal amplification, the primers were split into two pools. These primer pools and crRNA sequences are listed in Supplementary Table 4.

#### Target control: PIC1 and PIC2

The consensus sequences generated directly above after multiple genome alignment with MAFFT were used to order a 500-bp dsDNA fragment encompassing the primer and crRNA binding sites. RNA was generated according to the method described in ‘General mCARMEN procedures-Preparation of IVT material’ and diluted to 10^6^ copies μl^−1^ in pools based on the primer pools mentioned above (PIC1, PIC2). The PICs were used as input into the CARMEN v.1 or mCARMEN detection reaction to function as a detection-positive control.

#### Manual or automated sample extraction

Automated and manual extraction was performed according to methods described under ‘General mCARMEN procedures-Extraction’.

#### Two-step amplification

We followed the CARMEN v.1 platform for two-step reverse transcription amplification, which was performed first by complementary DNA synthesis and then by PCR.

#### cDNA synthesis using SuperScript IV

A total of 10 μl of extracted RNA was converted into single-stranded cDNA in a 40-μl reaction. First, Random Hexamer Primers (Thermo Fisher Scientific) were annealed to sample RNA at 70 °C for 7 min, followed by reverse transcription using SuperScript IV for 20 min at 55 °C. cDNA was stored at −20 °C until use. DNase treatment was not performed at any point during sample preparation.

#### Q5 DNA amplification

Nucleic acid amplification was performed via PCR using Q5 Hot Start High-Fidelity DNA Polymerase (New England Biolabs) using primer pools (with 150 nM of each primer) in 20-μl reactions. Amplified samples were added directly into the detection reaction or stored at −20 °C until use. The following thermal cycling conditions were used: (1) initial denaturation at 98 °C for 2 min; (2) 45 cycles at 98 °C for 15 s, 50 °C for 30 s and 72 °C for 30 s; and (3) final extension at 72 °C for 2 min. Each target was amplified with its corresponding primer pool as listed under ‘oligonucleotides used in this study’.

#### CARMEN v.1 detection

For color coding, unless specified otherwise, amplified samples were diluted 1:10 into nuclease-free water supplemented with 13.2 mM MgCl_2_ before color coding to achieve a final concentration of 6 mM after droplet merging. Detection mixes were not diluted. Color-coded stocks (2 µl) were arrayed in 96-well plates. (For detailed information on the construction of color codes, see ‘Color code design, construction and characterization’.) Each amplified sample or detection mix (18 µl) was added to a distinct color code and mixed by pipetting.

For emulsification, the color-coded reagents (20 µl) and 2% 008-fluorosurfactant (RAN Biotechnologies) in fluorous oil (3M 7500, 70 µl) were added to a droplet generator cartridge (Bio-Rad Laboratories) and reagents were emulsified into droplets using a QX200 droplet generator (Bio-Rad Laboratories) or a custom aluminum pressure manifold.

For droplet pooling, a total droplet pool volume of 150 µl of droplets was used to load each standard chip; a total of 800 µl of droplets was used to load each mChip. To maximize the probability of forming productive droplet pairings (amplified sample droplet + detection reagent droplet), half the total droplet pool volume was devoted to target droplets and half to detection reagent droplets. For pooling, individual droplet mixes were arrayed in 96-well plates. A multichannel pipette was used to transfer the requisite volumes of each droplet type into a single row of eight droplet pools, which were further combined to make a single droplet pool. The final droplet pool was pipetted up and down gently to fully randomize the arrangement of the droplets in the pool. The pooling step was rapid (<10 min) and the small-molecule exchange between droplets during this period did not substantially alter the color codes.

#### mCARMEN detection

We followed the methods under ‘General CARMEN Procedures-Detection-Fluidigm detection’ with the following modifications: 42.5 nM LwaCas13 and 212.5 nM crRNA in each assay mix reaction and 500 nM RNaseAlert v2 in each sample mix reaction.

#### CARMEN v.1 analysis

We followed the data analysis pipeline from CARMEN v.1 (ref. ^40^) to demultiplex and read out the fluorescence intensity of the reporter channel for each droplet reaction performed (MATLAB 2013). Briefly, premerge imaging data were processed using custom Python 3 scripts to detect fluorescently encoded droplets in microwells and identify their inputs based on their fluorescence intensity in 3 encoding channels, 647 nm, 594 nm and 555 nm. Subsequently, post-merge imaging data were analyzed to extract the reporter signal of the assay in the 488 nm channel and those reporter fluorescence intensities were physically mapped to the contents of each microwell. Quality control filtering was performed based on the appropriate size of a merged droplet from two input droplets and the closeness of a droplet’s color code to its assigned color code cluster centroid. The median and standard error were extracted from the replicates of all assay combinations generated on the array.

#### mCARMEN analysis

We followed the methods under ‘General CARMEN Procedures-Fluidigm Data Analysis’ and further visualized the data using Python 3, R v.4 and Prism 9 (GraphPad Software).

### Single-step amplification troubleshooting

The following RT–PCR kits were tested to determine the best performing assay: (1) OneStep RT–PCR Kit (QIAGEN); (2) TaqPath 1-Step Multiplex Master Mix (Thermo Fisher Scientific); (3) One-Step PrimeScript RT–PCR Kit (Takara Bio); (4) GoTaq Probe qPCR Kit (Promega Corporation); (5) UltraPlex 1-Step ToughMix (4X) (Quanta BioSciences); and (6) iTaq Universal One-Step Kit for RT–PCR (Bio-Rad Laboratories). Of the kits tested, the OneStep RT–PCR Kit (QIAGEN) was chosen for the final mCARMEN protocol.

#### Qiagen OneStep RT–PCR Kit

All or a combination of the following thermal cycling conditions were tested to shorten assay run time: reverse transcription at 50 °C for 15–30 min, PCR activation at 95 °C for 5–15 min, denaturation step at 94 °C for 10–30 s and extension step at 72 °C for 10 s to 1 min. The final extension at 72 °C for 10 min was omitted in all runs. The following primer pool conditions were also tested to optimize the assay: 150, 300, 500 and 600 nM of virus-specific primer and 100 and 150 nM of RNase P primers, with 5 μM of each virus-specific primer and 1.7 μM of RNase P primers. The reaction volumes tested include: 20 μl with 10% RNA template input, 30 μl with 20% RNA template input and 50 μl with 20% RNA template input. The final amplification conditions used for the RVP panel are described under ‘General mCARMEN Procedures-Amplification’.

#### TaqPath 1-Step Multiplex Master Mix Kit

The TaqPath 1-Step Multiplex Master Mix Kit was used to amplify nucleic acid according to the manufacturer’s instructions, using custom primer pools in 20-μl reactions. Primer pools of 150, 300 and 500 nM and annealing temperatures of 58 and 60 °C were all tested and compared to determine optimal conditions. The following thermal cycling conditions were used: (1) uracil-DNA glycosylase passive reference incubation at 25 °C for 2 min; (2) reverse transcription incubation at 50 °C for 15 min; (3) enzyme activation at 95 °C for 2 min; and (4) 40 cycles at 95 °C for 3 s and 60 °C for 30 s. Amplified samples were directly added into the detection reaction or stored at −20 °C until use.

#### GoTaq Probe qPCR Kit

The GoTaq Probe qPCR Kit was used to amplify nucleic acid via RT–PCR according to the manufacturer’s instructions, using custom primer pools in 20-μl reactions. Primer pools of 200, 300 and 500 nM were tested and compared to determine optimal conditions. Each target in the panel was amplified with its corresponding primer pool. The following thermal cycling conditions were used: (1) reverse transcription at 45 °C for 15 min and 95 °C for 2 min; and (2) 40 cycles at 95 °C for 15 s, 60 °C for 1 min. Amplified samples were directly added into the detection reaction or stored at −20 °C until use.

#### UltraPlex 1-Step ToughMix (4X)

The UltraPlex 1-Step ToughMix (4X) was used to amplify nucleic acid via RT–PCR according to the manufacturer’s instructions, using custom primer pools in 20-μl reactions. Primer pools of 200, 300 and 500 nM were tested and compared to determine optimal conditions. Each target in the panel was amplified with its corresponding primer pool. The following thermal cycling conditions were used: (1) reverse transcription at 50 °C for 10 min and 95 °C for 3 min; and (2) 45 cycles at 95 °C for 10 s, 60 °C for 1 min. Amplified samples were directly added into the detection reaction or stored at −20 °C until use.

### RVP testing at the Broad Institute research laboratory

#### Design of nine virus respiratory panel and RNase P

We designed this panel according to the methods described above under ‘Respiratory Panel-Design’ for these nine viruses: SARS-CoV-2, HCoV-HKU1, HCoV-OC43, HCoV-NL63, FLUAV/FLUAV-g4, FLUBV, HPIV-3, HRSV and HMPV, with the addition of an RNase P primer pair and crRNA. RNase P primers and crRNAs were designed within the same region of the gene as the CDC RT–qPCR assay (Supplementary Table 4).

#### Patient specimen validation

All patient specimens evaluated on the RVP were additionally evaluated concurrently with the CDC 2019-nCoV Real-Time RT–PCR Diagnostic Panel for N1 and RNase P. A specimen subset was selected for further study using NGS.

The CDC 2019-nCoV EUA recommends a Ct cutoff of <40 for RNase P and/or SARS-CoV-2. Eight specimens failed quality control metrics and were therefore removed from further analysis. Five specimens were previously positive and three were negative by prior RT–PCR testing done by the Broad Genomics Platform.

Of the 525 patient specimens evaluated by mCARMEN, only 2 specimens had no detectable levels of RNase P above threshold, 1 of which was positive for SARS-CoV-2 while the other was virus-negative. The RNase P-negative, but virus-positive, specimen likely had a high concentration of viral RNA, which sequestered amplification materials during the reaction, limiting RNase P amplification. The double-negative specimen suggested possible extraction failure or sample integrity issues and was thus excluded from further analysis.

#### RVP detection

Specimen preparation was performed according to the method outlined in ‘General mCARMEN Procedures-Sample extraction’ with 200 µl of input material. Amplification was performed according to the methods outlined in ‘General mCARMEN Procedures-Amplification’. Detection reactions were prepared as described in ‘General mCARMEN Procedures-Detection’ with the following modifications: 42.5 nM LwaCas13 and 212.5 nM crRNA in each assay mix reaction and 500 nM quenched synthetic fluorescent RNA reporter (FAM/rUrUrUrUrUrUrU/3IABkFQ/) in each sample mix reaction. Results were analyzed according to the methods outlined under ‘General mCARMEN procedures-Data analysis’.

#### CDC 2019-nCoV Real-Time RT–PCR Diagnostic Panel (research use only)

The CDC 2019-nCoV Real-Time RT–PCR Diagnostic Panel was performed using the TaqPath 1-Step RT–qPCR Master Mix (Thermo Fisher Scientific) with a 1-μl template RNA of either SARS-CoV-2 or RNase P in 10-μl reactions, run in triplicate. Primers from the 2019-nCoV RUO Kit (Integrated DNA Technologies) were used. For SARS-CoV-2, a primer pool at 800 μM and probe at 200 μM were used. For RNase P, a primer pool at 500 μM and a probe at 125 μM were used. The following thermal cycling conditions were used: (1) enzyme activation at 25 °C for 2 min; (2) reverse transcription at 50 °C for 15 min; (3) PCR activation at 95 °C for 2 min; and (4) 45 cycles at 95 °C for 3 s and 55 °C for 30 s. Standard curves were made with spike-in of the RNA template (SARS-CoV-2 and RNase P) to make a tenfold serial dilution from 10^0^ to 10^6^ copies μl^−1^. This was run on the QuantStudio 6 Flex Real-Time PCR System (Thermo Fisher Scientific).

#### NGS

Metagenomic sequencing libraries were generated as described previously^5,55^. Briefly, extracted RNA was DNase-treated to remove residual DNA then human ribosomal RNA was depleted. cDNA was synthesized using random hexamer primers. Sequencing libraries were prepared with the Illumina Nextera XT DNA Library Preparation Kit and sequenced with 100- or 150-nucleotide paired-end reads. Data analysis was conducted on the Terra platform (app.terra.bio); all workflows are publicly available on the Dockstore tool registry service. Samples were demultiplexed using demux_plus to filter out known sequencing contaminants. Viral genomes were assembled using assemble_refbased, discordant specimens with viral genomes were assembled using assemble_denovo and additionally visualized using classify_kraken, blastn, blastx, geneious and R. A virus was determined to be present if more than 10 reads mapped to a particular viral genome. Full genomes were deposited with GenBank (BioProject accession no. PRJNA802370).

### Clinical evaluation of RVP in CLIA-certified laboratory at MGH

#### Design

We designed this panel according to the methods described above under RVP Testing at the Broad Institute Research Laboratory.

#### Extraction control

Extraction negative control is an RNA extraction control and is prepared by adding 200 μl pooled human sample (negative for all viruses on the panel) to a well with 280 μl binding bead mix. The extraction negative control should yield a positive result for the RNase P crRNA and primer pair and a negative result for all other targets.

#### Amplification control

NTC is a negative control for nucleic acid amplification and is prepared by adding 10 μl nuclease-free water (instead of RNA) into 40 μl of OneStep RT–PCR Kit mastermix. This should yield a negative result for all targets on the panel. Combined positive control is a positive control for nucleic acid amplification and is prepared by pooling IVT synthetic RNA of all the targets on the panel to 10^3^ copy µl^−1^; 11-µl aliquots of this mix were stored at −80 °C until use, when 10 μl were added to 40 μl of OneStep RT–PCR Kit mastermix. This should yield a positive result for all targets on the panel.

#### Detection controls

Negative detection control is a negative control for the Fluidigm detection step and is prepared by adding nuclease-free water (instead of amplified RNA) to the sample mix without MgCl_2_. This should yield a negative result for all targets on the panel. No crRNA control is a negative control for the Fluidigm detection step and is prepared by adding nuclease-free water (instead of 1 µM crRNA) to the assay mix. This should yield a negative result for all targets on the panel.

#### Batch preparation of sample and assay mixtures

Sample and assay mixtures can be prepared in advance for multiple 96-sample batches according to similar methods, with the following changes: the batch sample mix contained all reagents described above excluding 9 mM MgCl_2_, and the batch assay mix contained all reagents described above excluding the 2x Assay Loading Reagent. Both mixtures were calculated with 10% coverage. Both mixtures were stored at −80 °C until use; 9 mM MgCl_2_ was added to the sample mix and 2x Assay Loading Reagent was added to each assay mix before use.

#### SYBR RT–qPCR of viral seed stock and genomic RNA

Quantification of all viral seed stock and genomic RNA received from ATCC and BEI Resources was performed using the Power SYBR Green RNA-to-C_T_ *1-Step* Kit (Thermo Fisher Scientific). Reactions were run in triplicate with 1 μl RNA input in 10-μl reactions. A primer mix at 500 nM was used and all primer sequences used are listed in Supplementary Table 4. The following thermal cycling conditions were used: (1) reverse transcription at 48 °C for 30 min; (2) enzyme activation at 95 °C for 10 min; (3) 40 cycles at 95 °C for 15 s and 60 °C for 1 min; and (4) melt curve of 95 °C for 15 s, 60 °C for 15 s and 95 °C for 15 s. Standard curves were made with spike-in of RNA template to make a tenfold serial dilution from 10^0^ to 10^6^ copies μl^−1^. This was run on the QuantStudio 6 Flex Real-Time PCR System.

#### LOD

Samples were prepared for the LOD experiments using either quantified viral isolates, genomic RNA or IVT partial gene fragments. For the SARS-CoV-2, HCoV-OC43, HRSV and HPIV-3 assays, quantified viral isolates of known titer (RNA copies µl^−1^) spiked into a pooled negative human sample (negative for all viruses on the panel) in universal transport medium (UTM), to mimic a clinical specimen. The pooled human negative samples were incubated in the binding bead mix solution according to the methods described in ‘General mCARMEN Procedures-Automated extraction’. Since no quantified virus isolates for HCoV-NL63, HCoV-HKU-1, FLUAV, FLUAV-g4, FLUBV and HMPV were available for use at the time the study was conducted, assays designed for RNA detection of these viruses were tested with either genomic RNA from ATCC (FLUAV: catalog no. NR-43756; FLUBV: catalog no. VR-1804) or IVT RNA of known titer and spiked into pooled negative human samples in UTM.

RNA was extracted from 200 µl of input material using the MagMAX DNA Multi-Sample Ultra 2.0 Kit on a KingFisher Flex Magnetic Particle Processor with 96 Deep Well Head. This was run according to the protocol listed in the TaqPath COVID-19 Combo Kit Protocol under ‘KingFisher, Extract RNA-Automated method (200-μl input volume)’ with the following differences. To prepare the binding bead mix, the following was added: 265 μl binding solution, 10 μl total nucleic acid magnetic beads, 5 μl Proteinase K with 10% coverage for multiple samples. Then, 280 μl of the binding bead mix was added to each sample well. The 200 μl of input material included negative human sample and RNA: 160 μl of pooled human samples (negative for all viruses on the panel) was added to each sample well and incubated for 20 min before 40 μl of RNA was spiked in. Samples were eluted in 50 μl of elution solution and either directly added to the amplification reaction or stored at −80 °C until use.

A preliminary LOD for each assay was determined by testing triplicates of RNA purified using the extraction method described in the ‘RVP panel’. The approximate LOD was identified by extraction, amplification and detection of tenfold serial dilutions of IVT RNA of known titer (copies µl^−1^) for five replicates. These concentrations ranged from 10^4^–10^−3^ copies μl^−1^. The lower bound of the LOD range was determined as the lowest concentration where five out of five replicates were positive; the upper bound was determined as the concentration tenfold above the lower bound.

A confirmation of the LOD for each assay was determined by testing 20 replicates of RNA purified using the extraction method described in the ‘RVP panel’. The approximate LOD was identified by extraction, amplification and detection of twofold serial dilutions of the input sample, quantified viral isolates and genomic RNA or IVT RNA. These concentrations ranged from 20 to 0.5 copies μl^−1^, depending on the virus. The LOD was determined as the lowest concentration where ≥95% (19 out of 20) of the replicates were positive.

### Specificity

#### In silico analysis: inclusivity

Inclusivity was tested by performing an in silico analysis using all publicly available sequences of all targets on the RVP panel. Complete genomes for all viruses were downloaded from NCBI on 2 April 2021 and aligned using MAFFT v.7. For viral species with fewer than 1,000 sequences, the FFT-NS-ix1000 algorithm was used to create the MAFFT alignment. For viral species with >1,000 sequences, the FFT-NS-1 algorithm was used to create the MAFFT alignment. The primer and crRNA sequences were then mapped to the aligned viral sequences using a consensus alignment to determine the percentage identity (homology) and the number of mismatches. The average homology and mismatches were taken across the total number of sequences evaluated. Please note that mismatches for crRNA sequences do not take wobble base pairing (G-U pairing) into account. Results are summarized in Supplementary Table 8.

Additionally, the SARS-CoV-2 crRNA and primer sequences were tested by NCBI BLAST+ against the nonredundant/nucleotide databases (updated 31 March 2021, *n* = 68,965,867 sequences analyzed) and the Betacoronavirus database (updated 1 April 2021, *n* = 140,760). The search parameters were adjusted to blastn-short for short input sequences. The match and mismatch scores were 1 and −3, respectively. The penalty to create and extend a gap in an alignment was 5 and 2, respectively. The BLAST results confirmed only perfect matches to SARS-CoV-2.

#### In silico analysis: specificity

Complete genomes for all viruses were downloaded from the NCBI on 2 April 2021 and aligned using MAFFT. For viral species with fewer than 1,000 sequences, FFT-NS-ix1000 was used. For viral species with >1,000 sequences, FFT-NS-1 was used for the MAFFT alignment. The primer and crRNA sequences were then mapped to the aligned viral sequences using a consensus alignment to determine percentage identity (homology). The average homology was taken across the panel sequences and the total number of sequences were evaluated. The text in bold represents on-target primers/crRNA to the intended viral sequences. Not all sequence combinations were evaluated because whole-genome homology between many viruses is less than 80%. All primer and crRNA sequences do not have >80% homology to other unintended viral or bacterial sequences, making the panel highly specific to particular viruses of interest. More specifically, no in silico cross-reactivity >80% homology between any primers and crRNA sequences on the RVP was observed for the following common respiratory flora and other viral pathogens: SARS-CoV-1, HCoV-MERS, adenovirus, enterovirus, rhinovirus, *Chlamydia pneumoniae*, *Haemophilus influenzae*, *Legionella pneumophila*, *Mycobacterium tuberculosis*, *Streptococcus pneumoniae*, *Streptococcus pyogenes*, *Bordetella pertussis*, *Mycoplasma pneumoniae*, *Pneumocystis jirovecii*, *Candida albicans*, *Pseudomonas aeruginosa*, *Staphylococcus epidermis* and *Streptococcus salivarius*. The results of the in silico analysis are summarized in Supplementary Table 9.

#### In vitro analysis

Targets were selected for in vitro specificity testing based on closely related viral species with high nucleotide identity. The synthetic DNA targets contained the consensus sequence of a particular virus that was position-matched to the location of the RVP virus of interest targets in the viral genome. Samples were prepared for the specificity experiments according to the methods described above in ‘General mCARMEN procedures-Preparation of IVT material’; samples were serially diluted down to a concentration of 10^6^ and 10^5^ copies µl^−1^. For all samples prepared for the specificity experiments, RNA was extracted from 200 µl of input material using the MagMAX DNA Multi-Sample Ultra 2.0 Kit on a KingFisher Flex Magnetic Particle Processor. This was run according to the extraction, amplification and detection methods described above under ‘RVP testing at the Broad Institute research laboratory’.

### Patient specimen validation

#### Specimen preparation before extraction

All patient specimens from the MGH Clinical Microbiology Laboratory were initially reported to be positive for HCoV-HKU1, HCoV-NL63 and HMPV via BioFire FilmArray Respiratory Panel (RP2) (Biofire Diagnostics) or positive for SARS-CoV-2, FLUAV (H3), FLUBV and HRSV via Xpert Xpress SARS-CoV-2/Flu/RSV (Cepheid). SARS-CoV-2 was aliquoted as follows: 220 μl for testing using the RVP panel and 220 μl for testing using the TaqPath COVID-19 Combo Kit; remaining specimens were stored at −80 °C. All negative specimens were aliquoted as follows: 220 μl for RVP panel testing, 220 μl for TaqPath COVID-19 Combo Kit testing and 400 μl for BioFire FilmArray Respiratory Panel (RP2) testing; remaining specimen was stored at −80 °C. All other specimens were aliquoted as follows: 220 μl for RVP panel testing and 400 μl for BioFire FilmArray Respiratory Panel (RP2) testing; remaining specimen was stored at −80 °C.

#### Preparation of contrived samples before extraction

Contrived patient samples of the HCoV-HKU1, HCoV-OC43, HCoV-NL63, FLUAV-g4, HPIV-3 and HMPV viruses were prepared by diluting either viral seed stock (HCoV-OC43 and HPIV-3) or template RNA (HCoV-HKU1 and HCoV-NL63). See Supplementary Table 10 for viral seed stock vendor details. Viral seed stock or template RNA was added to non-pooled human specimens (negative for all targets, except RNase P, on the RVP panel) at a concentration 2 times the LOD; the concentrations for these samples ranged from 10^2^ to 10^6^ copies µl^−1^.

#### RVP

All materials were extracted, amplified, detected and analyzed using the methods described under ‘General mCARMEN procedures’ and ‘RVP testing at the Broad Institute research laboratory-Patient specimen validation’.

#### TaqPath COVID-19 Combo Kit

A subset of patient specimens, all SARS-CoV-2 and negative patient specimens, from the MGH Clinical Microbiology Laboratory were verified using the TaqPath COVID‑19 Combo Kit. These samples were initially reported to be positive for SARS-CoV-2 via Xpert Xpress SARS-CoV-2/Flu/RSV or reported to be negative for all targets (excluding RNase P) on the RVP panel via BioFire FilmArray Respiratory Panel (RP2). The TaqPath COVID‑19 Combo Kit was performed according to the manufacturer’s instructions. The assay was performed using the Applied Biosystems 7500 Real-Time PCR System.

#### BioFire FilmArray Respiratory Panel (RP2)

A subset of patient specimens from the MGH Clinical Microbiology Laboratory, all HCoV-HKU1, HCoV-NL63, FLUAV (H3), FLUBV, HRSV, HMPV and negative patient specimens, were verified using the BioFire FilmArray Respiratory Panel (RP2). These specimens were either initially reported to be positive for HCoV-HKU1, HCoV-NL63 and HMPV via BioFire FilmArray Respiratory Panel (RP2) or positive for FLUAV (H3), FLUBV and HRSV via Xpert Xpress Flu/RSV (Cepheid). For each run, one patient specimens in UTM at 300 µl was verified using the BioFire FilmArray Respiratory Panel (RP2) according to the manufacturer’s instructions. Any remaining specimen was stored at −80 °C.

Controls for this assay were received with the kit and ready for use. Control 1 was expected to be positive for adenovirus, HMPV, human rhino/enterovirus, FLUAV (H1-2009), FLUAV (H3), HPIV-1 and HPIV-2. Control 2 was expected to be positive for HCoV-229E, HCoV-HKU1, HCoV-NL63, HCoV-OC43, FLUAV (H1), FLUBV, HPIV-2, HPIV-3 and HRSV.

The results were automatically displayed on the FilmArray System with each target in a run reported as ‘detected’ or ‘not detected’. If either control failed, the software marked this run as ‘invalid’. When sufficient human sample volume was available, samples with invalid results were rerun.

#### Analysis software

The analysis software comprises Python scripts executing the data analysis described under ‘General CARMEN Procedures-Fluidigm Data Analysis.’ They were packaged into an executable with graphical user interface using the Python module Gooey v.1.0.7.

Briefly, the reference-normalized, background-subtracted fluorescence was calculated for the guide-target pairs for measurement after 60 min. Then, the dynamic range and the separation band were assessed: separation band = (mean of positive controls − 3 s.d. of positive controls) − (mean of negative controls − 3 s.d. of negative controls); dynamic range = mean of positive controls − mean of negative controls.

If the ratio of separation band to dynamic range was ≤0.2, the whole assay was invalid. Next, for the positive and negative controls, outliers based on three s.d. were identified. If a positive control had a too low value, or a negative control had a too high value, the respective assay was invalid. For the remaining samples, hit calling was performed based on comparing the signal to the water control. If the signal was 1.8× higher than the water control, the guide-target pair was called a hit. Based on this hit calling, the extraction control, negative and positive detection controls and internal controls were verified. If their result did not correspond to their expected hit status, either the respective assay or specimen was deemed invalid. To be valid, all specimens needed to be either positive for RNase P or at least one other assay. Finally, the software annotated the results as CSV files and visualized them as an annotated heatmap.

### Cas13- and Cas12-based detection with mCARMEN

#### Design for Cas12-based detection

Cas13 crRNAs from the RVP were utilized. Cas12 crRNAs were manually designed in the same region of the viral genome as the Cas13 crRNAs to reduce the need for additional primer design while maintaining the protospacer adjacent motif requirements of Cas12. Only one additional primer was designed to properly amplify all targets on the RVP. All crRNAs and primers are listed in Supplementary Table 4.

#### Detection

We followed the methods outlined under ‘General mCARMEN procedures-Detection’ with the following modifications: 10–60 nM LwaCas13, 10–60 nM LbaCas12a, 125 nM Cas13a crRNA and 125 nM Cas12 crRNA in each assay mix reaction and 500 nM quenched synthetic fluorescent RNA reporter (FAM/rUrUrUrUrUrUrU/3IABkFQ/ and VIC/rTrTrArTrTrArTrT/3IABkFQ) in each sample mix reaction.

#### Data analysis

We generally followed the methods outlined under ‘General mCARMEN procedures-Analysis’, this time taking into account the VIC signal separate from the FAM signal. We used a custom Python script to determine whether the FAM signal of a reaction was significantly above background by comparing it to the NTC. If the background-subtracted and normalized fluorescence intensity was 1.8 higher than the normalized and background-subtracted NTC, the assay was considered positive.

### Variant testing

#### Design

The crRNAs for SNP discrimination were designed using a generative sequence design algorithm (Mantena, S. et al., manuscript in preparation). This approach uses ADAPT’s predictive model to predict the activity of candidate crRNA sequences against on-target and off-target sequences^35^. These predictions of candidate crRNA activity steer the generative algorithm’s optimization process, where it seeks to design crRNA probes that have maximal predicted on-target activity and minimal predicted off-target activity. Using this design algorithm, we selected 26 mutations to detect and discriminate between the variants (Supplementary Table 4).

#### Amplification: SuperScript IV One-Step RT–PCR System

The SuperScript IV One-Step RT–PCR System was used to amplify nucleic acids according to the manufacturer’s instructions, using custom primer pools in 25-μl reactions. Primer pools were made at 10 μM. The following thermal cycling conditions were used: (1) reverse transcription incubation at 50 °C for 15 min; (2) enzyme activation at 98 °C for 2 min; (3) 35–40 cycles at 98 °C for 10 s, 60 °C for 10 s and 72 °C for 1 min 30 s; and (4) final extension at 72 °C for 5 min. Amplified samples were directly added into the detection reaction or stored at −20 °C until use.

#### Detection

We followed the methods outlined under ‘General mCARMEN procedures-Detection’ with the following modifications: 42.5 nM LwaCas13 and 2–212.5 nM crRNA in each assay mix reaction.

#### Data analysis: threshold calculation

To determine if an ancestral or derived sequence was present, the signals between respective ancestral and mutation crRNA pairs had to be evaluated and compared (Supplementary Table 4). First, background-subtracted reporter fluorescence was normalized to the background-subtracted passive reference dye (ROX) fluorescence for each assay in the IFC. Next, the ancestral:mutation and mutation:ancestral ratios were calculated for each 5-min interval time point across 180 min. For each crRNA pair, the ratio reaching a crRNA pair-specific threshold at the earliest time point was selected. If the ancestral:mutation ratio was selected, then the sequence present was determined to be ancestral. If the mutation:ancestral ratio was selected, then the sequence present was determined to contain the mutation targeted by the mutation crRNA within the SARS-CoV-2 spike gene. crRNA pair-specific thresholds were determined based on ancestral and variant control samples, also referred to as seed stock samples, tested in parallel with the unknown samples. For a given crRNA pair, the threshold was set to the lowest value with the maximum combined sensitivity and specificity when applied to the seed stock samples. For crRNA to detect an SNP at the same position, the second lowest threshold with the maximum combined sensitivity and specificity was chosen if possible, without compromising the maximum combined sensitivity and specificity. For crRNA pairs targeting mutations not represented in the variant control samples, a default crRNA pair threshold of 1.5 was set.

The variant identified hit-calling parameters were as follows: (1) if no mutations were detected, a result of ‘ancestral’ was returned; (2) at least one unique crRNA specific to a single SARS-CoV-2 variant must be above the fluorescence ratio threshold. If there was no unique crRNA signal above threshold, a result of ‘variant not identified’ was returned; (3) if two or more mutations for a given variant fell below the threshold, a result of ‘variant not identified’ was returned. All other mutations must surpass the threshold; (4) if three or more unexpected mutations for a given variant were above threshold, a result of ‘variant not identified’ was returned. At most, two unexpected signals could occur as long as parameters 1 and 2 were met.If all three parameters were met, a result of ‘variant identified’ was returned. If the parameters were not met, a result of ‘variant not identified’ was returned. Samples that contained additional mutation signals falling outside the typical variant lineage mutation list follow these parameters: (1) if 1–2 unexpected signals were observed to be slightly above the threshold yet all other signals were correct for a specific variant lineage, then the unexpected signal was disregarded and the variant call was made on the remaining signals. (2) if more than two unexpected signals were observed above the threshold and either all other signals were correct for a specific variant lineage or were not perfectly matching, a result of ‘variant uncertain’ was returned.

Please note that the variant identification pipeline will need to be updated as new SARS-CoV-2 mutations and variant lineages arise for proper identification.

A few exceptions are worth mentioning: we observed crRNAs for SNPs E484Q, P681R, N501T and L452Q, which had undesirable cross-reactive signals with a position-matched or adjacent mutation; thus, they were excluded from further evaluation.

### Reporting Summary

Further information on research design is available in the Nature Research Reporting Summary linked to this article.

## Online content

Any methods, additional references, Nature Research reporting summaries, source data, extended data, supplementary information, acknowledgements, peer review information; details of author contributions and competing interests; and statements of data and code availability are available at 10.1038/s41591-022-01734-1.

### Supplementary information

Supplementary Information Supplementary Figs. 1–7 and Tables 1–11 (titles and captions only).

Reporting Summary

Supplementary Tables See Supplementary Information File for all 11 table titles.

Supplementary Data mCARMEN standard operating procedure.

## Data Availability

All requests for raw and analyzed data and materials will be reviewed by the Broad Institute of Harvard and MIT to verify if the request is subject to any intellectual property or confidentiality obligations. Data and materials that can be shared will be released via a material transfer agreement. RNA sequencing data have been deposited with the Sequence Read Archive under the BioProject accession no. PRJNA802370 and will be made available on request for academic use and within the limitations of the provided informed consent by the corresponding author upon acceptance.

## Code availability

The code used for data analysis in this study is available on Github: https://github.com/broadinstitute/mcarmen.
