# Author Correction: Multiplexed CRISPR-based microfluidic platform for clinical testing of respiratory viruses and identification of SARS-CoV-2 variants

**DOI:** 10.1038/s41591-023-02684-y

**Published:** 2023-11-09

**Authors:** Nicole L. Welch, Meilin Zhu, Catherine Hua, Juliane Weller, Marzieh Ezzaty Mirhashemi, Tien G. Nguyen, Sreekar Mantena, Matthew R. Bauer, Bennett M. Shaw, Cheri M. Ackerman, Sri Gowtham Thakku, Megan W. Tse, Jared Kehe, Marie-Martine Uwera, Jacqueline S. Eversley, Derek A. Bielwaski, Graham McGrath, Joseph Braidt, Jeremy Johnson, Felecia Cerrato, Gage K. Moreno, Lydia A. Krasilnikova, Brittany A. Petros, Gabrielle L. Gionet, Ewa King, Richard C. Huard, Samantha K. Jalbert, Michael L. Cleary, Nicholas A. Fitzgerald, Stacey B. Gabriel, Glen R. Gallagher, Sandra C. Smole, Lawrence C. Madoff, Catherine M. Brown, Matthew W. Keller, Malania M. Wilson, Marie K. Kirby, John R. Barnes, Daniel J. Park, Katherine J. Siddle, Christian T. Happi, Deborah T. Hung, Michael Springer, Bronwyn L. MacInnis, Jacob E. Lemieux, Eric Rosenberg, John A. Branda, Paul C. Blainey, Pardis C. Sabeti, Cameron Myhrvold

**Affiliations:** 1 Broad Institute of MIT and Harvard, Cambridge, MA USA; 2 Harvard Program in Virology, Division of Medical Sciences, Harvard Medical School, Boston, MA USA; 3 Department of Biological Engineering, Massachusetts Institute of Technology, Cambridge, MA USA; 4 Division of Infectious Diseases, Department of Medicine, Massachusetts General Hospital, Boston, MA USA; 5 Wellcome Sanger Institute, Wellcome Genome Campus, Hinxton, UK; 6 Harvard Program in Biological and Biomedical Sciences, Harvard Medical School, Boston, MA USA; 7 Division of Health Sciences and Technology, Harvard Medical School and Massachusetts Institute of Technology, Cambridge, MA USA; 8 Department of Organismic and Evolutionary Biology, Harvard University, Cambridge, MA USA; 9 Harvard/Massachusetts Institute of Technology MD-PhD Program, Harvard Medical School, Boston, MA USA; 10 Department of Systems Biology, Harvard Medical School, Boston, MA USA; 11 State Health Laboratories, Rhode Island Department of Health, Providence, RI USA; 12 Massachusetts Department of Public Health, Boston, MA USA; 13 Influenza Division, National Center for Immunization and Respiratory Diseases, Centers for Disease Control and Prevention, Atlanta, GA USA; 14 African Centre of Excellence for Genomics of Infectious Diseases, Redeemer’s University, Ede, Nigeria; 15 Department of Biological Sciences, College of Natural Sciences, Redeemer’s University, Ede, Nigeria; 16 Molecular Biology Department and Center for Computational and Integrative Biology, Massachusetts General Hospital, Boston, MA USA; 17 Department of Immunology and Infectious Disease, Harvard T.H. Chan School of Public Health, Harvard University, Boston, MA USA; 18 Department of Pathology, Massachusetts General Hospital and Harvard Medical School, Boston, MA USA; 19 Koch Institute for Integrative Cancer Research at Massachusetts Institute of Technology, Cambridge, MA USA; 20 Howard Hughes Medical Institute, Chevy Chase, MD USA; 21 Department of Molecular Biology, Princeton University, Princeton, NJ USA

**Keywords:** Biotechnology, Infectious-disease diagnostics

Correction to: *Nature Medicine* 10.1038/s41591-022-01734-1, published online 7 February 2022.

In the version of the article originally published, some of the oligonucleotide sequences in Supplementary Table 4, on the “21 viruses” and “RVP” tabs, were mislabeled. The Supplementary Tables file has now been corrected.

