# Supplementary material for: Multiplexed CRISPR-based microfluidic platform for clinical testing of respiratory viruses and identification of SARS-CoV-2 variants
Source: Nat Med. 2022 Feb 7;28(5):1083–94. doi: 10.1038/s41591-022-01734-1 (PMC9117129; doi:10.1038/s41591-022-01734-1)
Supplement: Supplementary file 1 — Supplementary Figs. 1–7 and Tables 1–11 (titles and captions only). [file 41591_2022_1734_MOESM1_ESM.pdf]

nature portfolio

https://doi.org/10.1038/s41591-022-01734-1

Supplementary information

Multiplexed CRISPR-based microfluidic platform for clinical testing of respiratory viruses and identification of SARS-CoV-2 variants

In the format provided by the authors and unedited

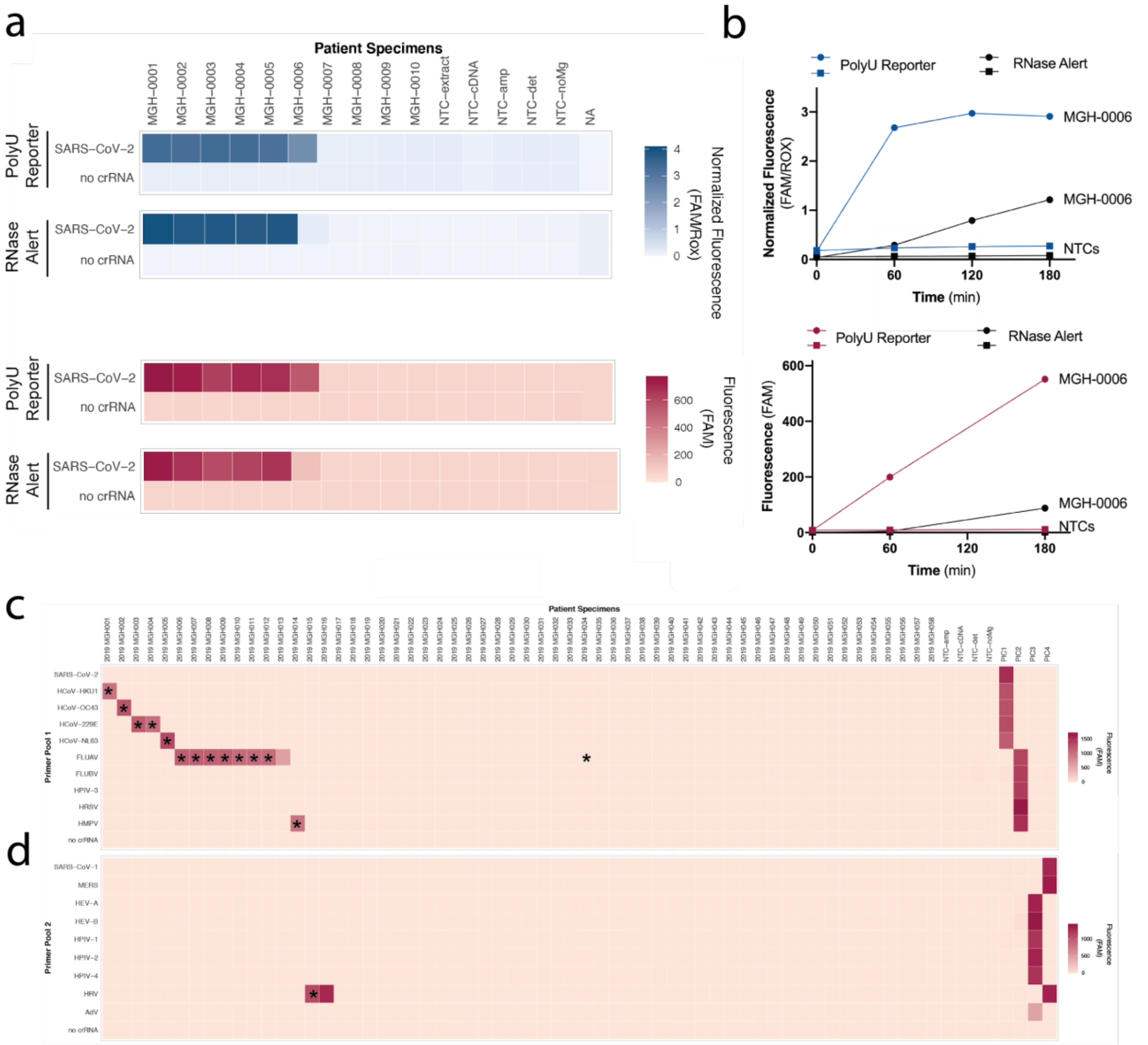

Supplementary Figure 1. Enhanced sensitivity with polyU fluorescent reporter. a, 6 SARS-CoV-2 positive and 4 SARS-CoV-2 negative NP swabs from Fig. 1e. mCARMEN values are shown as normalized fluorescence (blue) and CARMEN v1 values are shown as raw fluorescence (red) while signal using the polyU reporter is shown directly above signal using RNase Alert (Thermo). **b,** Fluorescence over time for SARS-CoV-2 positive patient specimen, MGH-006. Top, mCARMEN fluorescence values for MGH-006 and NTC with blue line representing signal from the polyU reporter; black line representing signal from RNase Alert at 0, 60, 120, and 180 mins post-reaction initiation. Bottom, CARMEN v1 fluorescence values, red line representing signal from polyU reporter and black representing signal from RNase Alert at 0, 60, and 180 mins post-reaction initiation. **c&d,** CARMEN v1 signal shown as raw fluorescence at 3 hrs post-reaction initiation. 58 patient specimens were extracted then amplified in 2 steps with 2 separate primer pools. **c,** Amplification from primer pool 1 **d,** amplification from primer pool 2. All specimens were background subtracted using NTC-amp as a negative control. Asterisks (*) represent specimens with known infections by unbiased NGS, more than 10 reads mapped to the viral genome.

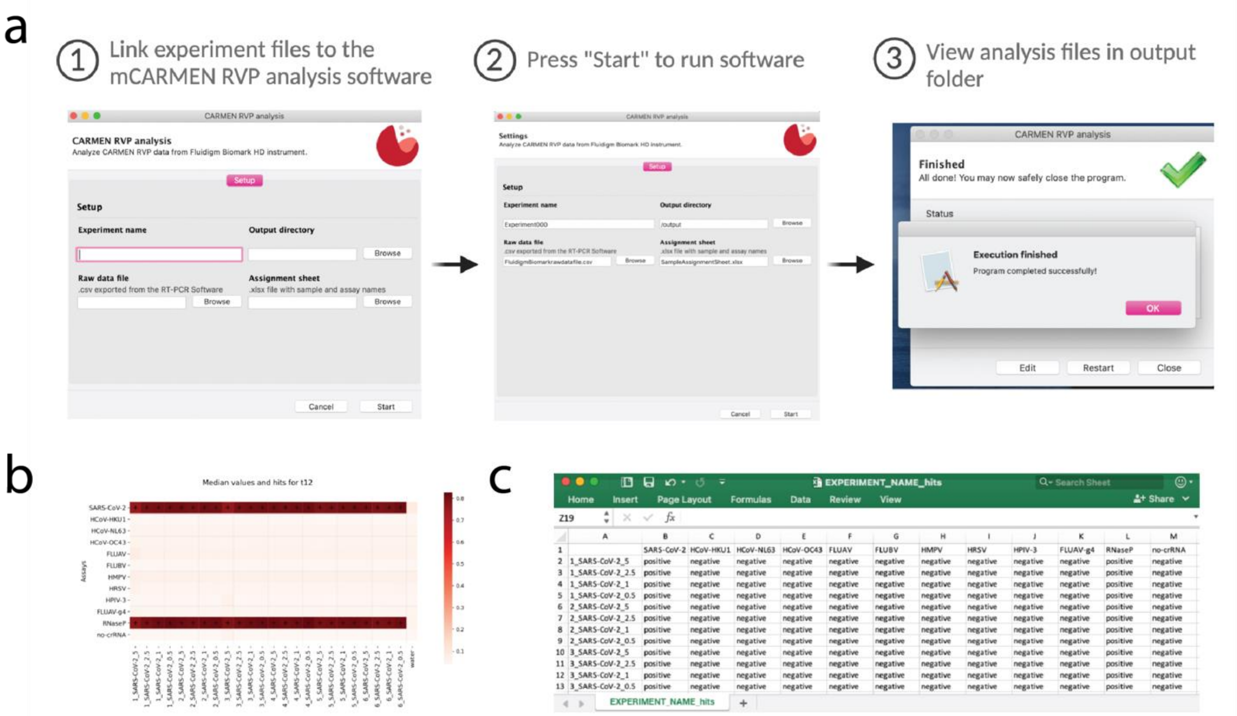

Supplementary Figure 2. mCARMEN RVP Software for automated results and hit calling. **a,** RVP Analysis Software workflow. (Step 1) Once the software has been successfully launched, four sections must be filled in: Experiment name, Output directory, Raw data file, and Assignment sheet. The raw data file comes from the Biomark instrument after the RVP run is complete. The assignment sheet must be filled out prior to software analysis. (Step 2) Press start to run the RVP Software analysis. (Step 3) The analysis can take several minutes to run, but once complete the user can navigate to the specified Output directory to view the six output files. **b,** Example of a condensed heatmap from a successful RVP experiment ([Experiment Name_heatmap_t12.png). **c,** Snapshot of the key file that captures all the RVP results individually broken down by patient specimen ID and respiratory virus (Experiment Name_HitQuantification.csv). The file lists the Specimen IDs accompanied by a positive, negative, or invalid determination, as defined in Clinical Evaluation of "Clinical Evaluation of RVP at MGH - Controls & Software Analysis." The RVP Software is available at: https://github.com/broadinstitute/carmen-rvp.

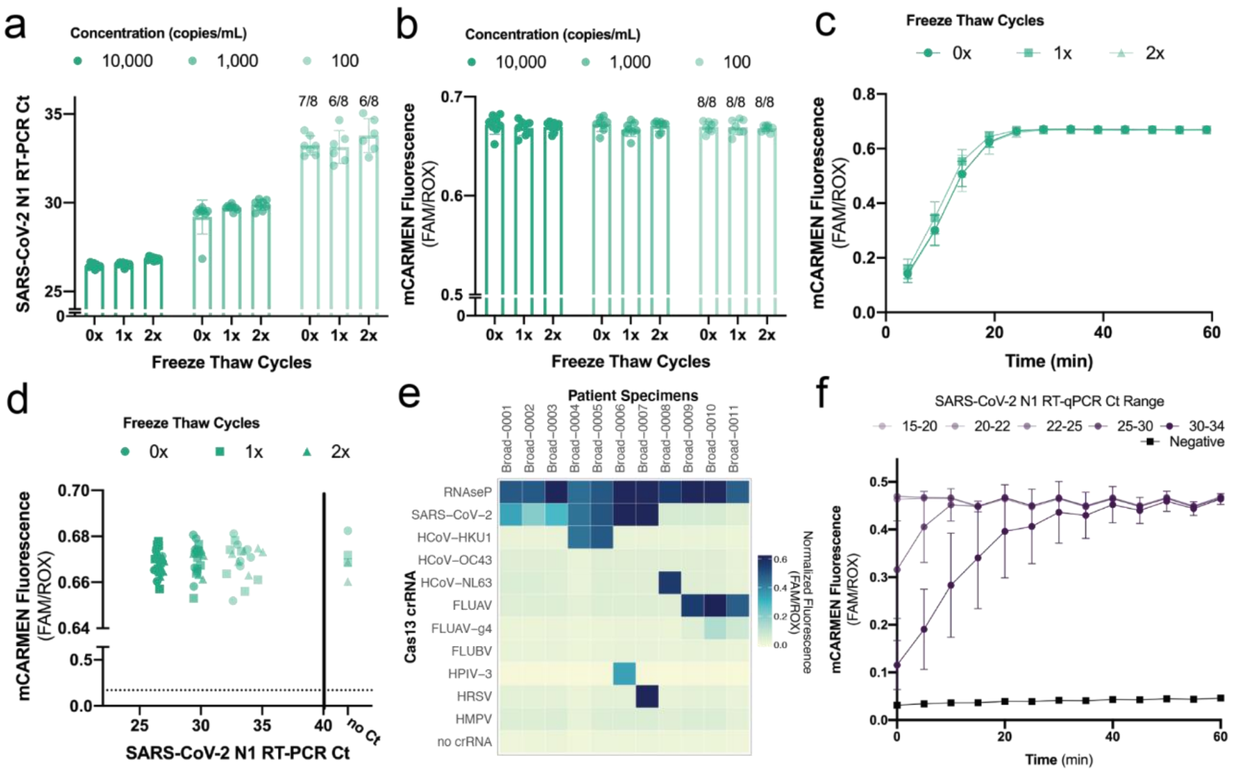

Supplementary Figure 3. Benchmarking mCARMEN RVP sensitivity to gold standard, RT-PCR. a-d,Comparison of mCARMEN RVP fluorescence to SARS-CoV-2 N1 Ct values from RT-PCR across 3 serial dilution series of contrived SARS-CoV-2 samples (100-10,000 copies/mL, n=8) after 0x, 1x, or 2x freeze thaw cycles. **a**, SARS-CoV-2 N1 Ct values across 3 serial dilution series after 0x, 1x, or 2x freeze thaw cycles for 8 replicates. **b**, RVP SARS-CoV-2 normalized fluorescence values across 3 serial dilution series after 0x, 1x, or 2x freeze thaw cycles for 8 replicates at 1 hr post-reaction initiation. **c**, RVP kinetic curves for SARS-CoV-2 at 100 copies/mL across the different freeze thaw cycles. **d**, Scatter plot of RVP fluorescence compared to N1 Ct for all replicate dilutions and freeze thaw cycles. Circle: 0x freeze thaw; Square: 1x freeze thaw; Triangle: 2x freeze thaw; colored from high-to-low concentration. **e**, Normalized fluorescence values at 1 hr post-reaction initiation for 11 of the 525 patient specimens evaluated by RVP and RT-PCR in Fig. 2. **f,** Average normalized fluorescence for SARS-CoV-2 across patient specimens within Ct ranges of 15-20 (n=8), 20-22 (n=13), 22-25 (n=5), 25-30 (n=8), and 30-34 (n=11). Purple lines: known SARS-CoV-2 positive specimens; black line: known SARS-CoV-2 negative specimens; error bars: one standard deviation from the mean fluorescence.

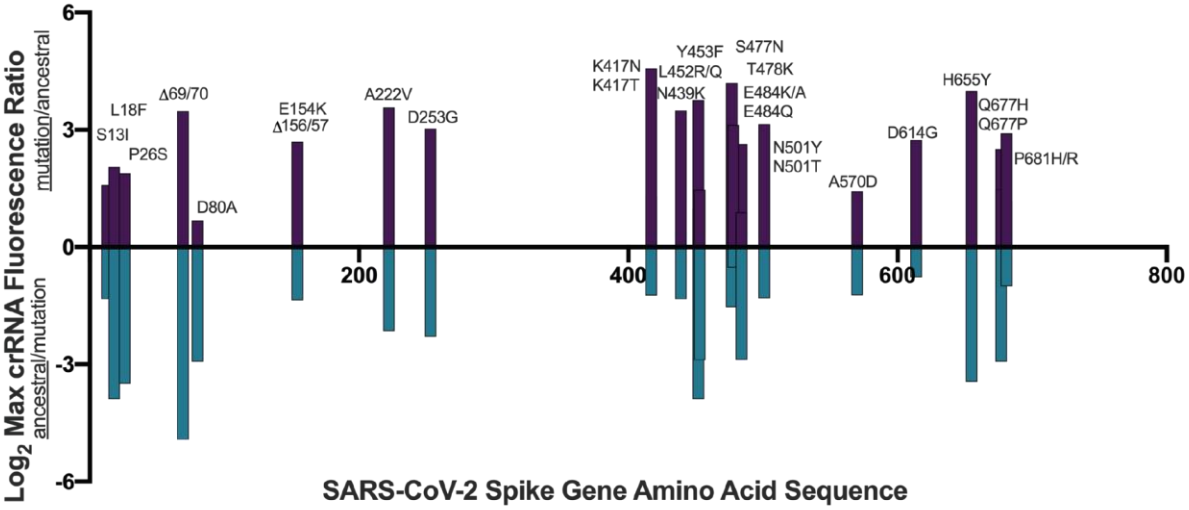

Supplementary Figure 4. Evaluation of crRNA pairs detecting SARS-CoV-2 spike gene mutations on synthetic targets. 26 mutations across the SARS-CoV-2 spike gene were selected based on uniqueness within a variant lineage or due to phenotypic effects on viral fitness. *In vitro* transcribed synthetic RNA targets at 10^{13} copies/mL were used as input. Top, positive values, represents signal on the derived sequence (mutation/ancestral) and bottom, negative values, represents signal on the ancestral sequence (ancestral/mutation). Bars represent $\log_2$ maximum crRNA normalized fluorescence ratio at any 5 min time point from 0-180 mins post-reaction initiation.

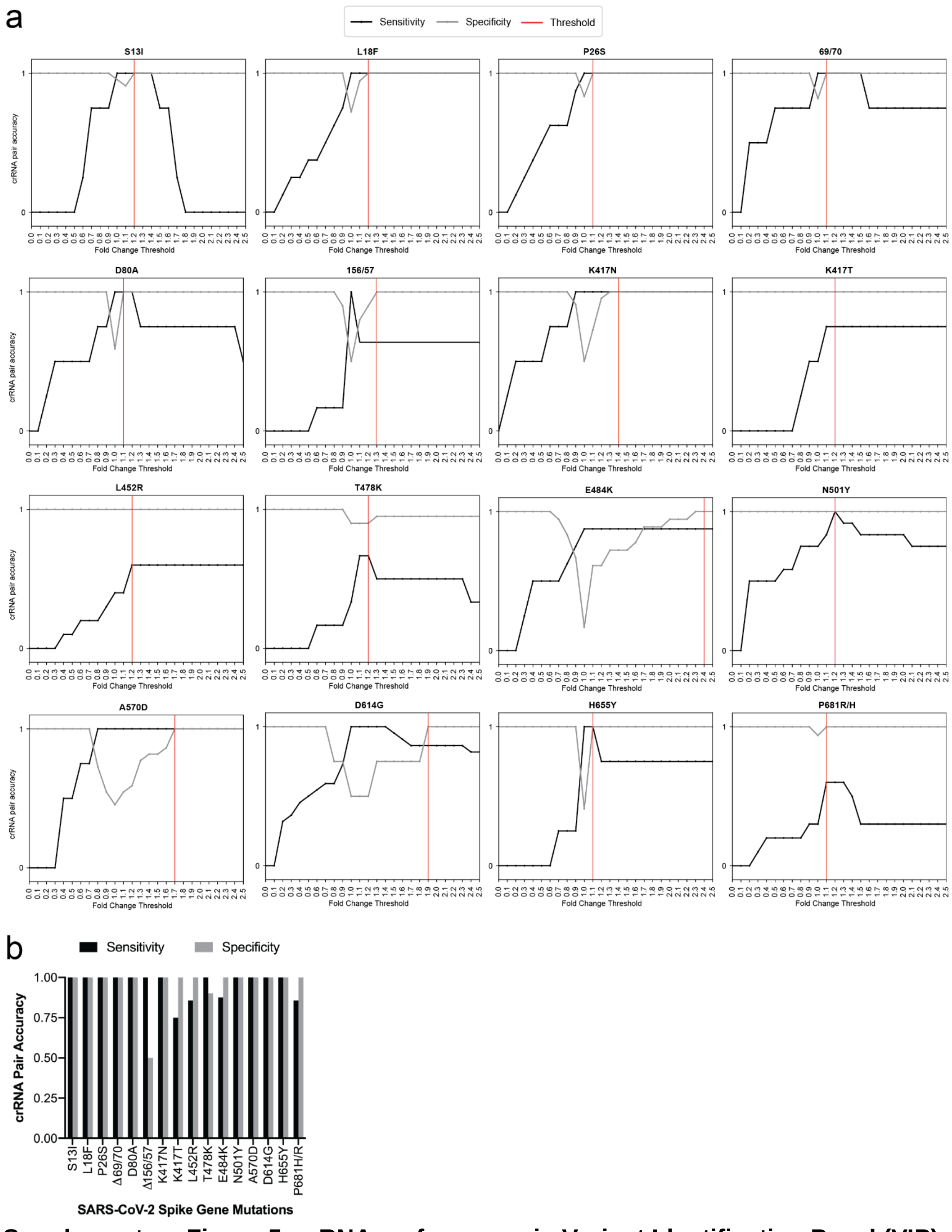

Supplementary Figure 5. crRNA performance in Variant Identification Panel (VIP). a, Evaluation of sensitivity and specificity of crRNA pair detection to establish mutation calling threshold. All values based on viral seed stock testing. Black line: sensitivity/true positive rate; gray line: specificity/true negative rate; red line: mutation calling threshold. **b,** Summary of sensitivity and specificity results from **a**. Black: sensitivity; gray: specificity.

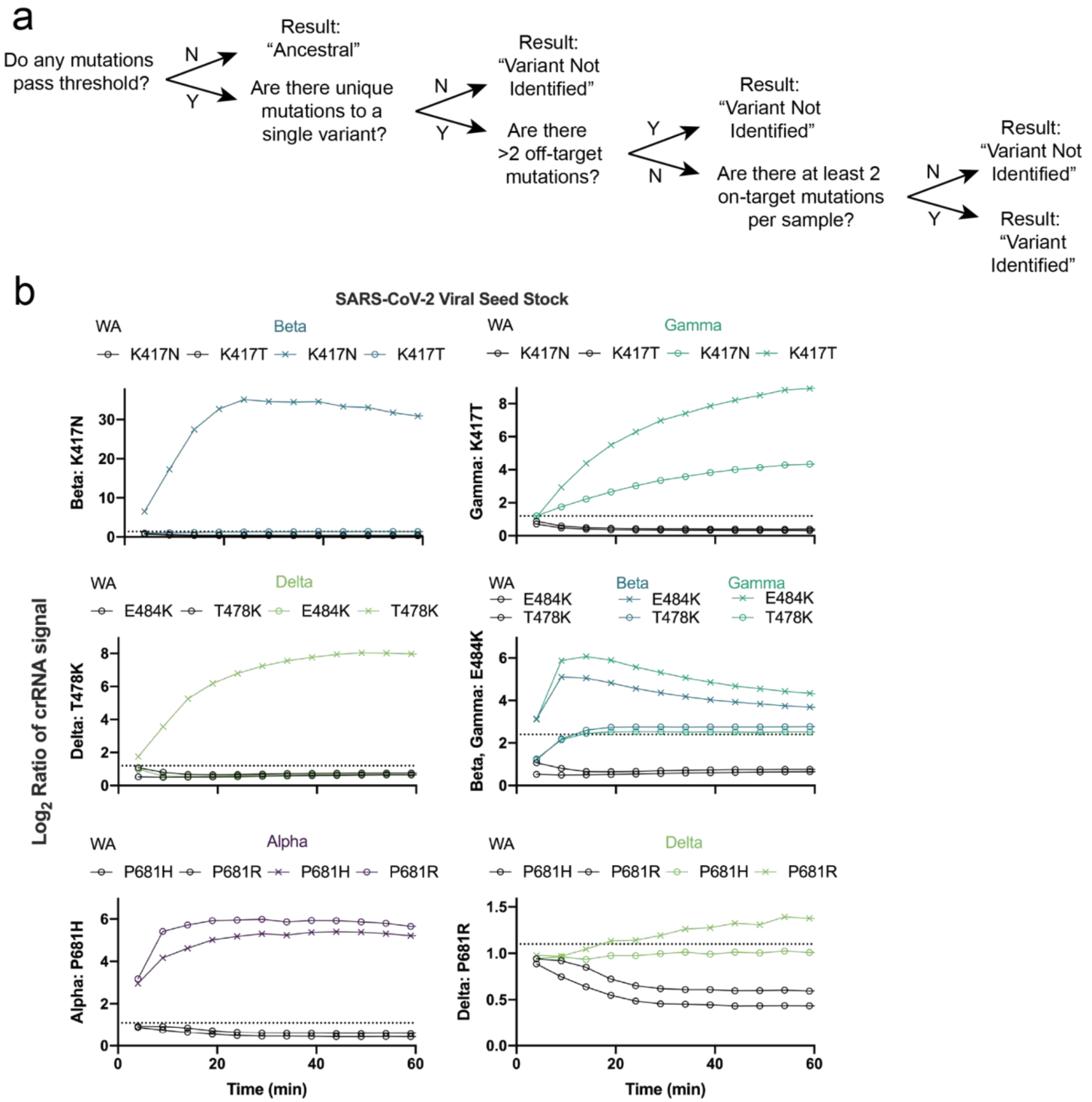

Supplementary Figure 6. General VIP analysis pipeline workflow and special cases. a, Variant identification analysis pipeline method for making variant calls based on fluorescence signals from VIP assay. **b**, Alpha, Beta, Gamma, and Delta viral seed stocks were evaluated using VIP. Kinetic curve data shown as the $\log_2$ of the maximum crRNA fluorescence ratio of mutation/ancestral at five min intervals for the first 60 min post-reaction initiation. Black: ancestral seed stock WA; purple: Alpha; blue: Beta; teal: Gamma; green: Delta. X lines represent the expected SNP based on NGS. O lines represent the unexpected SNP based on NGS. Dashed line represents threshold for variant calling based on Supplementary Fig. 5.

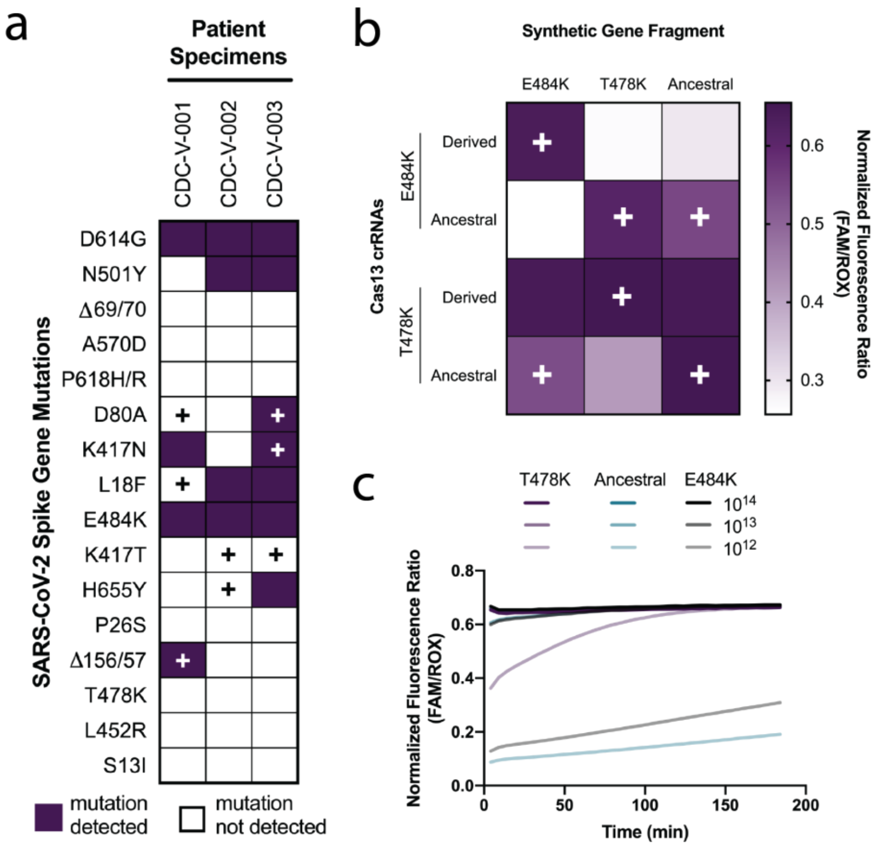

Supplementary Figure 7. SARS-CoV-2 variant lineage discrepancies between mCARMEN VIP and NGS. a, Mutation calls from the variant identification analysis pipeline for 3 SARS-CoV-2 positive patient specimens from Fig. 5c-e where the analysis pipeline returns either a not identified result or an incorrectly identified variant result. Black plus sign (+) represents signal that should be present and while plus sign (+) represents signal that should not be present for the given variant lineage based on NGS results. **b**, Heatmap of the normalized fluorescence at 1 hr post-reaction initiation for E484K and T478K crRNAs on synthetic gene fragments containing the ancestral sequence or either the T478K or E484K SNP. Plus sign (+) represents signal that is expected for the given variant lineage based guide design method. **c**, Kinetics curves for T478K crRNA detection of synthetic gene fragments from the ancestral strain or containing either the T478K or E484K SNP. Data shown as normalized fluorescence every 5 mins for 3 concentrations of target 10^{14}-10^{12} copies/mL. Purple: expected variant lineage; Blue: ancestral lineage; Black: unexpected variant lineage; colored from high-to-low concentration.

Supplementary Table 1. Cost calculation breakdown concerning mCARMEN.

Supplementary Table 2. Oligonucleotides related to this work.

Supplementary Table 3. Comparison chart of mCARMEN and CARMEN v1.

Supplementary Table 4. Comparison chart of CARMEN technologies to other nucleic acid-based diagnostics.

Supplementary Table 5. Patient specimens related to this work.

Supplementary Table 6. Preliminary limit of detection (LOD) results using synthetic targets from 1-10,000,000 copies/mL. A range finding study for LOD for each target was conducted using synthetic materials spiked into pooled negative patient specimens stored in Universal Transport Medium (UTM). The range finding study was conducted with a dilution series ranging from 1 - 10,000,000 copies/mL. The estimated LOD range was determined for each viral target between the lowest dilution detected and the highest undetected concentration for the confirmatory experiments.

Supplementary Table 7. Performance summary of mCARMEN RVP clinical evaluation at MGH.

Supplementary Table 8. *In silico* inclusivity evaluation of CARMEN-RVP. Inclusivity was evaluated by performing an *in silico* analysis using all publicly available sequences of all targets on the panel. Inclusivity was tested by performing an *in silico* analysis using all publicly available sequences of all targets on the panel. Complete genomes for all viruses were downloaded from NCBI on April 2nd 2021 and aligned using MAFFT, a multiple sequence alignment program that stands for “Multiple Alignment using Fast Fourier Transform.” MAFFT utilizes several different alignment algorithms. For viral species with less than 1000 sequences, the FFT-NS-ix1000 algorithm was used to create the MAFFT alignment. For viral species with >1000 sequences, the FFT-NS-1 algorithm was used to create the MAFFT alignment. The primer and crRNA sequences were then mapped to the aligned viral sequences using a consensus alignment to determine the percent identity (homology) and the number of mismatches. The average homology and mismatches were taken across the total number of sequences evaluated. Please note that mismatches below for crRNA sequences do not take wobble base pairing (G-U pairing) into account. Additionally the SARS-CoV-2 crRNA and primer sequences were compared by NCBI BLAST+ against the nr/nt databases (updated 03/31/2021, N=68965867 sequences analyzed) and the Betacoronavirus database (updated 04/01/2021, N=140760). The search parameters were adjusted to blastn-short for short input sequences. The match and mismatch scores are 1 and -3, respectively. The penalty to create and extend a gap in an alignment is 5 and 2, respectively. Blast results confirmed only perfect matches to SARS-CoV-2.

Supplementary Table 9. *In silico* Cross-reactivity (analytical specificity) evaluation of CARMEN-RVP. Complete genomes for all viruses were downloaded from NCBI on April 2nd 2021 and aligned using MAFFT. For viral species with less than 1000 sequences, FFT-NS-ix1000 was used. For viral species with >1000 sequences, FFT-NS-1 was used for the MAFFT alignment. The primer and crRNA sequences were then mapped to the aligned viral sequences using a consensus alignment to determine percent identity (homology). The average homology was taken across the panel sequences and the total number of sequences evaluated. Bolded text represents on-target primers/crRNA to the intended viral sequences. Not all sequence combinations were evaluated since whole genome homology between many viruses is significantly less than 80%. None of the primer or crRNA sequences has >80% homology to other, unintended viral or bacterial sequences, making the panel highly specific to the particular

viruses of interest. More specifically, no *in silico* cross-reactivity >80% homology between any primers and crRNA sequences on the CARMEN-RVP assay is observed for the following common respiratory flora and other viral pathogens: SARS-CoV-1, HCoV-MERS, Adenovirus, Enterovirus, Rhinovirus, *Chlamydia pneumoniae, Haemophilus influenzae, Legionella pneumophila, Mycobacterium tuberculosis, Streptococcus pneumoniae, Streptococcus pyogenes, Bordetella pertussis, Mycoplasma pneumoniae, Pneumocystis jirovecii, Candida albicans, Pseudomonas aeruginosa, Staphylococcus epidermis, Streptococcus salivarius*.

Supplementary Table 10. Viral seed stock and genomic RNA information related to this work.

Supplementary Table 11. Expected mutations for 6 SARS-CoV-2 variant lineages
