# Supplementary material for: Multiplexed CRISPR-based microfluidic platform for clinical testing of respiratory viruses and identification of SARS-CoV-2 variants
Source: Nat Med. 2022 Feb 7;28(5):1083–94. doi: 10.1038/s41591-022-01734-1 (PMC9117129; doi:10.1038/s41591-022-01734-1)
Supplement: Supplementary file 2 — Reporting Summary [file 41591_2022_1734_MOESM2_ESM.pdf]

nature portfolio

Corresponding author(s): Nicole Welch, Cameron Myhrvold, Pardis Sabeti

Last updated by author(s): Feb 1, 2022

Reporting Summary

Nature Portfolio wishes to improve the reproducibility of the work that we publish. This form provides structure for consistency and transparency in reporting. For further information on Nature Portfolio policies, see our Editorial Policies and the Editorial Policy Checklist.

Statistics

For all statistical analyses, confirm that the following items are present in the figure legend, table legend, main text, or Methods section.

n/a	Confirmed	
☐	☒	The exact sample size (*n*) for each experimental group/condition, given as a discrete number and unit of measurement
☐	☒	A statement on whether measurements were taken from distinct samples or whether the same sample was measured repeatedly
☐	☒	The statistical test(s) used AND whether they are one- or two-sided<br>*Only common tests should be described solely by name; describe more complex techniques in the Methods section.*
☐	☒	A description of all covariates tested
☐	☒	A description of any assumptions or corrections, such as tests of normality and adjustment for multiple comparisons
☐	☒	A full description of the statistical parameters including central tendency (e.g. means) or other basic estimates (e.g. regression coefficient) AND variation (e.g. standard deviation) or associated estimates of uncertainty (e.g. confidence intervals)
☐	☒	For null hypothesis testing, the test statistic (e.g. *F*, *t*, *r*) with confidence intervals, effect sizes, degrees of freedom and *P* value noted<br>*Give P values as exact values whenever suitable.*
☒	☐	For Bayesian analysis, information on the choice of priors and Markov chain Monte Carlo settings
☒	☐	For hierarchical and complex designs, identification of the appropriate level for tests and full reporting of outcomes
☒	☐	Estimates of effect sizes (e.g. Cohen's *d*, Pearson's *r*), indicating how they were calculated

Our web collection on statistics for biologists contains articles on many of the points above.

Software and code

Policy information about availability of computer code

Data collection	Data was collected on the Fluidigm Biomark. Droplet data was collected using Matlab 2013 for microscope and camera control.
Data analysis	Python3 custom analysis codes available on github: broadinstitute/mcarmen, MAFFT v7 and ADAPT were used for primer design, crRNA design and in silico specificity analysis. RStudio 4 and Prism 9 were used for plotting.

For manuscripts utilizing custom algorithms or software that are central to the research but not yet described in published literature, software must be made available to editors and reviewers. We strongly encourage code deposition in a community repository (e.g. GitHub). See the Nature Portfolio guidelines for submitting code & software for further information.

Data

Policy information about availability of data

All manuscripts must include a data availability statement. This statement should provide the following information, where applicable:

- Accession codes, unique identifiers, or web links for publicly available datasets
- A description of any restrictions on data availability
- For clinical datasets or third party data, please ensure that the statement adheres to our policy

Sequencing data will be made publicly available on the SRA under the BioProject Accession # PRJNA802370. Raw data may be made available upon request.

Field-specific reporting

Please select the one below that is the best fit for your research. If you are not sure, read the appropriate sections before making your selection.

☒ Life sciences ☐ Behavioural & social sciences ☐ Ecological, evolutionary & environmental sciences

For a reference copy of the document with all sections, see nature.com/documents/nr-reporting-summary-flat.pdf

Life sciences study design

All studies must disclose on these points even when the disclosure is negative.

Sample size	No sample-size calculations were performed. Samples evaluated were based on availability or FDA standards for clinical validation.
Data exclusions	There were only two samples that were excluded from data analysis. One SARS-CoV-2 positive sample tested in an academic setting that was positive by RT-qPCR but by mCARMEN signal was was too high in every crRNA channel including negative controls and thus had to be excluded because proper call could not be made. One HCoV-HKU1 sample from clinical evaluation at MGH was excluded because it tested positive for HCoV-HKU1 by BioFire, but upon repeated tested by BioFire, mCARMEN, and NGS there was no human internal control detected and there were minimal human reads suggesting the sample was heavily degraded. Since initial submission more samples have been excluded. There were 8 samples excluded from Fig. 2 because they did not pass quality control metrics set by the CDC nCoV-2019 ROU kit for RNase P. There were also samples excluded from Fig. 6 that did not contain enough viral RNA resulting in a Variant Not Identified (VNI) call by our analysis pipeline. All samples tested where known positive by RT-qPCR however leftover material was given for our evaluation resulting in not enough material in several cases thus the samples were excluded.
Replication	Each experiment includes at least 2 technical replicates per data point and up to 20 replicates for limit of detection experiments. Patient sample testing was done over multiple experiments and days. All attempts at data replication were successful.
Randomization	Samples were not randomized into groups, as samples were not grouped.
Blinding	Blinding was performed for the 58 presumed respiratory virus sample testing. Blinding was not possible for some samples due to the nature of the patient cohorts selected (they were known to be disease-positive).

Reporting for specific materials, systems and methods

We require information from authors about some types of materials, experimental systems and methods used in many studies. Here, indicate whether each material, system or method listed is relevant to your study. If you are not sure if a list item applies to your research, read the appropriate section before selecting a response.

Materials & experimental systems

n/a	Involved in the study	
☒	☐	Antibodies
☒	☐	Eukaryotic cell lines
☒	☐	Palaeontology and archaeology
☒	☐	Animals and other organisms
☐	☒	Human research participants
☒	☐	Clinical data
☒	☐	Dual use research of concern

Methods

n/a	Involved in the study	
☒	☐	ChIP-seq
☒	☐	Flow cytometry
☒	☐	MRI-based neuroimaging

Human research participants

Policy information about studies involving human research participants

Population characteristics	Population characteristics are unknown for all specimens in this study. Specimens were collected throughout the state of Massachusetts specifically at Broad Institute's Genomics Platform CLIA Laboratory, Massachusetts General Hospital, and Harvard Longwood Campus, but were provided de-identified for our use in this manuscript.
Recruitment	No recruitment was done for this study. Specimens were provided by the Broad Institute's Genomics Platform CLIA Laboratory, Massachusetts General Hospital, and Harvard Longwood Campus.
Ethics oversight	Use of clinical excess of human specimens from patients with SARS-CoV-2 from the Broad Institute's Genomics Platform CLIA Laboratory was approved by the MIT IRB Protocol #1612793224. Additional SARS-CoV-2 samples were collected from consented individuals under Harvard Longwood Campus IRB #20-1877 and covered by an exempt determination (EX-7295) at the Broad Institute. Human specimens from patients with SARS-CoV-2, HCoV-HKU1, HCoV-NL63, FLUAV, FLUBV, HRSV, and HMPV were obtained under a waiver of consent from the Mass General Brigham IRB Protocol #2019P003305.

Note that full information on the approval of the study protocol must also be provided in the manuscript.
