# Supplementary material for: Multiplexed CRISPR-based microfluidic platform for clinical testing of respiratory viruses and identification of SARS-CoV-2 variants
Source: Nat Med. 2022 Feb 7;28(5):1083–94. doi: 10.1038/s41591-022-01734-1 (PMC9117129; doi:10.1038/s41591-022-01734-1)
Supplement: Supplementary file 4 — mCARMEN standard operating procedure. [file 41591_2022_1734_MOESM4_ESM.pdf]

mCARMEN Respiratory Virus Panel (mCARMEN-RVP) Standard Operating Protocol

Table of Contents

Intended Use
Summary and Explanation
Principles of the Procedure

Materials and Reagents Required
Instrumentation, Equipment and Consumables Required

Warnings and Precautions
Reagent Storage, Handling, and Stability
Specimen Collection, Handling, and Storage

Reagent, Controls, and Equipment Preparation

Detailed Assay Protocol
Nucleic Acid Extraction
RT-PCR master mix preparation and RT-PCR amplification
Preparation of Cas13 Detection reactions and Sample Master Mix plate
Priming and loading the Fluidigm IFC
Running the IFC on the IFC Controller
Running and imaging on the Fluidigm Biomark

Data Analysis Using CARMEN-RVP Software

Interpretation of Results and Reporting

Intended Use

The mCARMEN-RVP assay is a multiplexed CRISPR-Cas13-based test intended for qualitative detection of nucleic acid from nine distinct respiratory viruses in nasopharyngeal swabs from individuals suspected of viral respiratory infection, including COVID-19, by their healthcare provider. Testing is limited to the Clinical Microbiology Laboratory at Massachusetts General Hospital, which is certified under the Clinical Laboratory Improvement Amendments of 1988 (CLIA), 42 U.S.C.§263a to perform high-complexity laboratory tests.

Results are for the detection of SARS-CoV-2, Human betacoronavirus strain HKU1, Human alphacoronavirus strain NL63, Human betacoronavirus strain OC43, Influenza A (FLUAV, human-associated subtypes), Influenza B (FLUBV), Human metapneumovirus (HMPV), human respiratory syncytial virus (HRSV), and human parainfluenza virus type 3 (HPIV-3) RNA. Viral RNA is generally detectable in respiratory specimens during the acute phase of infection. Positive results are indicative of the presence of viral RNA; clinical correlation with patient history and other diagnostic information is necessary to determine patient infection status. Positive results do not rule out bacterial infection or infection with other viruses not included in the mCARMEN-RVP assay. The results of this test should not be used as the sole basis for diagnosis, treatment, or other patient management decisions. The agent detected may not be the definite or only cause of symptoms or disease.

Laboratories within the United States and its territories are required to report all positive results to the appropriate public health authorities. Testing with the mCARMEN-RVP assay is intended for use by trained laboratory personnel who are proficient in performing high complexity molecular diagnostic assays. The assay is only for use under the Food and Drug Administration's Emergency Use Authorization.

Negative results should be considered presumptive and do not preclude current or future infection with viruses targeted by the assay, obtained through community transmission or other exposures. Negative results should not be used as the sole basis for diagnosis, treatment or other patient management decisions. Negative results obtained from individuals who are exhibiting symptoms associated with respiratory viral infection at the time of specimen collection should be interpreted with particular caution. Negative results must be considered in the context of an individual’s recent exposures, history, presence of clinical signs and symptoms consistent with COVID-19.

Summary and Explanation

The mCARMEN-RVP is a CRISPR/Cas13-based assay for detection of RNA from 9 separate respiratory viruses from nasopharyngeal swabs collected from individuals who meet CDC criteria for COVID-19 testing. The target viruses are listed below in *SOP Table 1: Viruses targeted by mCARMEN-RVP.*

SOP Table 1: Viruses targeted by mCARMEN-RVP

Virus Name	Acronyms	Viral Gene Targeted
Severe acute respiratory syndrome coronavirus 2	SARS-CoV-2	Orf1ab
Human coronavirus NL63	HCoV-NL63	Orf1ab
Human coronavirus OC43	HCoV-OC43	Orf1ab
Human coronavirus HKU1	HCoV-HKU1	Orf1ab
Influenza A	FLUAV	PB1
Influenza B	FLUBV	PB1
Human respiratory syncytial virus	HRSV	M
Human metapneumovirus	HMPV	F
Human parainfluenza virus 3	HPIV-3	M

Principles of the Procedure

The mCARMEN-RVP is a molecular *in vitro* diagnostic test that aids in the detection and differentiation of RNA from 9 different viral targets: SARS-CoV-2, HCoV-NL63, HCoV-OC43, HCoV-HKU1, FLUAV, FLUBV, HRSV, HMPV, HPIV-3. mCARMEN-RVP is based on widely used nucleic acid extraction and amplification (RT-PCR) technology, a novel CRISPR Cas13 detection system, and imaging of fluorescent on the Fluidigm Biomark system.

The workflow in short is as follows:

1. RNA is extracted from patient samples with the ThermoFisher KingFisher Flex MagMAX Viral/Pathogen Nucleic Acid Isolation kit
2. Patient sample RNA is combined with custom oligonucleotide primer pools and amplified using the Qiagen OneStep RT-PCR kit
3. Amplified product is combined with a Sample Master Mix that contains a quenched fluorescent reporter (FAM-7-PolyU Reporter) in a 96-well plate format
4. Separately, Cas13, T7-polymerase, and individual crRNAs (10) are combined to produce 10 Assay Master Mixes in a 12-strip tube format
5. The Assay Master Mixes are pipetted into the assay inlets on a Fluidigm 192.24 Integrated Fluidics Circuit (IFC)
6. The amplified products plus Sample Master Mix are pipetted into the Sample inlets on a Fluidigm 192.24 IFC
7. The Fluidigm 192.24 IFC is placed on a Fluidigm IFC RX Controller and run the “Load & Mix” protocol
8. The Fluidigm 192.24 IFC is transferred to and imaged on a Fluidigm Biomark
9. The image is analyzed with the mCARMEN-RVP Software. mCARMEN-RVP Software automatically validates the various controls, and then makes one of 3 calls: detected, not detected or “invalid” for every combination of sample and viral target

Materials and Reagents Required
Materials and reagents are required at each of the 5 physical areas of the mCARMEN-RVP workflow:
1. Pre-amplification / isolation
2. Pre-amplification / clean area
3. Pre-amplification / target area
4. Post-amplification / detection
5. Post-amplification / Fluidigm hardware

Materials and reagents are also broken down into 3 broad categories related to their use in the workflow:
1. Extraction
2. Amplification
3. Detection

Materials & reagents are listed below in *SOP Table 2: Materials and Reagents*

SOP Table 2: Materials and Reagents

Reagent Category	Component / kit	Manufacturer	Catalog #	Storage Conditions	Areas Needed
Extraction	MagMAX^{TM} Viral/Pathogen Nucleic Acid Isolation Kit	Thermo Fisher	A42352		1
Amplification	QIAGEN OneStep RT-PCR Kit	QIAGEN	210215		2
Amplification	Primers (forward and reverse, per target, per *Appendix 1: mCARMEN-RVP Primers, crRNA, and Reporter sequences*)	Eton	?	−20℃	2
Detection	CRISPR RNA (crRNA, per target, per *Appendix 1: mCARMEN-RVP Primers, crRNA, and Reporter sequences*)	IDT	?	−80℃	4
Detection	Cas13	Genscript		−80℃	4
Detection	RNase inhibitor (murine)	NEB		−20℃	4
Detection	T7 RNA polymerase	Lucigen		−20℃	4
Detection	rNTP mix @ 25 mM each (mixed at 100 mM total)	NEB		−20℃	4
Detection	Sterile, nuclease free water (non-DEPC treated)			RT	1, 2, 4

Detection	Loading Reagent (20x)	Fluidigm			
Detection	16uM FAM-7-PolyU Reporter	IDT		−20℃	4
Detection	ROX dye (50x)				4
Detection	1M Tris-HCl (pH 7.5)				
Detection	0.1M DTT				4
	100% Ethanol			RT	1
	70% Ethanol			RT	All
Detection	MgCl2				4
	Bleach			RT	All
	RNase AWAY Spray/Wipes			RT	1, 2
	RNase AWAY Spray/Wipes	Thermo Scientific 7002	VWR 17810-491	RT	3, 4, 5
Detection	Control Line Fluid Kit—192.24	Fluidigm			
Detection	192.24 GE DYNAMIC ARRAY kit (reagents for 10 IFCs)	Fluidigm		−20℃	4

Instrumentation, Equipment and Consumables Required

Instrumentation, Equipment and Consumable are required at each of the 5 physical areas of the mCARMEN-RVP workflow:

1. Pre-amplification / isolation
2. Pre-amplification / clean area
3. Pre-amplification / target area
4. Post-amplification / detection
5. Post-amplification / Fluidigm hardware

Instrumentation, Equipment and Consumables are listed below in *SOP Table 3: Instrumentation required for mCARMEN-RVP workflow, SOP Table 4: Equipment required for mCARMEN-RVP workflow*, and *SOP Table 5: Consumables required for mCARMEN-RVP workflow .*

SOP Table 3: Instrumentation required for mCARMEN-RVP workflow

Instrument	Manufacturer		Area Needed
Fluidigm Biomark	Fluidigm		5
Fluidigm IFC Controller (RX)	Fluidigm		5
KingFisher Flex Purification system	ThermoFisher	5400630	2
Mastercycler Pro Thermal Cycler	Eppendorf	EP6331000025-1EA	3
GENIE® SI-0236 Vortex-Genie 2 Mixer	Cole-Parmer	EW-04724-00	1
Tube Capper/Decapper; Thermo Scientific; 8-Channel Handheld	ThermoFisher	4105MAT	
Microplate centrifuge, PCR Plate Spinner	ThermoFisher	14-100-143	3, 5
Small refrigerator (4℃, temporary storage)			5
Micro-centrifuge			1

SOP Table 4: Equipment required for mCARMEN-RVP workflow

Equipment	Manufacturer	Catalog Number	Area Needed
Start Kit PL-LTS 2,20,200,1000µL	Rainin	30386597	2, 4
Pipet-X Pipet Controller PX-100	Rainin	17011733	1

Mylar™ Plate Sealer for Microtiter™ Plates, Mylar	ThermoFisher	5701TS1	1
Tube Capper/Decapper; Thermo Scientific; 8-Channel Handheld	Thermo Fisher	4105MAT	1, 2, 3, 4
Vortex	Global industrial	T9FB893093	3, 5
Pipet-Lite LTS Pipette L-1000XLS+	Rainin	17014382	1
Pipet-Lite Multi Pipette L8-200XLS+ Item #: 17013805 Pipet-LiteTM XLS+ manual 8-channel pipette, 20-200 µL	Rainin	17013810	2
Pipet-Lite Pipette Multi L12-1200XLS+	Rainin	17014497	1
Pipet-Lite™ XLS+ manual 12-channel pipette, 20-200 µL,	Rainin	17013810	1
Pipet-Lite™ XLS+ manual 8-channel pipette, 0.5-10 µL	Rainin	17013802	5
Pipet-Lite™ XLS+ manual 8-channel pipette, 2-20 µL	Rainin	17013803	1, 3. 5

SOP Table 5: Consumables required for mCARMEN-RVP workflow

Consumable	Manufacturer	Catalog Number	Area Needed
Reagent reservoirs (50 ml)			1
96-well plates			2, 3, 4
PCR strip tubes			
50 ml serological pipettes			1
KingFisher Deepwell plates	ThermoFisher	95040450	1
Elution plates	ThermoFisher	97002540	1
Tip combs	ThermoFisher	A43074	1
Reagent Reservoirs, VistaLab,	VWR	10770-296	1, 2, 3, 4

One-Compartment Tray; Mini Reservoir			
192.24 GE IFCs (10)	Fluidigm		
Plate seals (foil)			All
Kim wipes (large)			All
Centrifuge tubes (15ml)			1
PP centrifuge tubes, self-standing, 50 mL	Corning	CLS430921-500EA	1
Matrix 0.5mL Screw Top Tubes (for RNA storage)		B3744	1
Tube, 5ml DNA/RNA LoBind(200/PK)			1, 2, 4
Pipette tips (P10)			1, 4, 5
Pipette tips (P20)			1, 2, 3, 5
Pipette tips (P200)			1, 2, 4
Pipette tips (P1000)			1, 2

Additional Instrumentation, Equipment and Consumables Not Explicitly Listed

- **Freezer storage:** ≤ −70℃, −20℃
- **Appropriate PPE:** Gloves, safety glasses, face shields, sleeves, lab coats/gowns, etc.
- **Various Laboratory Storage:** Tube racks, ice trays, freezer storage containers, etc.

Warnings and Precautions

1. Follow standard precautions. All patient specimens and positive controls should be considered potentially infectious and handled accordingly.

2. Do not eat, drink, smoke, apply cosmetics or handle contact lenses in areas where reagents and human specimens are handled.

3. Refer to Biosafety in Microbiological and Biomedical Laboratories (BMBL) 5th Edition BMBL (http://www.cdc.gov/biosafety/publications/bmbl5/index.htm) for standard biological safety guidelines for all procedures.

4. Specimen processing should be performed in accordance with national biological safety regulations.

5. If infection with SARS-CoV-2 is suspected based on current clinical and epidemiological screening criteria recommended by public health authorities, specimens should be collected using appropriate infection control precautions.

6. Perform all manipulations of potentially infectious virus specimens within a Class II (or higher) biological safety cabinet.

7. Use personal protective equipment such as (but not limited to) gloves, sleeves and lab coats when handling kit reagents while performing this assay and handling materials including samples, reagents, pipettes, and other equipment and reagents.

8. Amplification technologies such as RT-PCR, and CRISPR-Cas13 detection assays are sensitive to accidental introduction of products from previous amplification reactions and introduction of RNase P from the performer or the environment. Incorrect results could occur if either the clinical specimen or the reagents used in the amplification or detection step become contaminated by accidental introduction of amplification product (amplicon) and RNase P.

9. Workflow in the laboratory should proceed in a unidirectional manner.

10. Maintain separate areas for assay setup and handling of nucleic acids.

11. Always check the expiration date of reagents prior to use. Do not use expired reagent(s). Do not substitute or mix reagents from different kit lots or from other manufacturers.

12. Change pipette tips between all manual liquid transfers.

13. During preparation of samples, compliance with good laboratory techniques is essential to minimize the risk of cross-contamination between samples, and the inadvertent introduction of

nucleases into samples during and after the extraction procedure. Proper aseptic technique should always be used when working with nucleic acids.

14. Maintain separate, dedicated equipment (e.g., pipettes, microcentrifuges) and supplies (e.g., microcentrifuge tubes, pipette tips) for assay setup and handling of extracted nucleic acids.

15. Wear a clean lab coat and powder-free disposable gloves (not previously worn) when setting up assays.

16. Change gloves between sample handling and whenever contamination is suspected.

17. Keep reagent and reaction tubes capped or covered as much as possible.

18. Primers, crRNA (including aliquots), and enzyme master mix must be thawed and maintained on cold block or ice at all times during preparation and use.

19. Work surfaces, pipettes, and centrifuges should be cleaned and decontaminated with cleaning products such as 10% bleach or 5% lysol, and “RNase AWAY® ” to minimize risk of nucleic acid or nuclease contamination. Residual bleach should be removed using 70% ethanol.

20. Reagents and patient sample RNA should be maintained on a cold block or on ice during preparation and use to ensure stability.

21. Dispose of unused kit reagents and human specimens according to local, state, and federal regulations.

22. Extraction Lysis Buffers contain guanidinium thiocyanate or guanidine-containing materials which can create highly reactive and/or toxic compounds if combined with sodium hypochlorite (bleach).

Reagent Storage, Handling, and Stability

- Store all primers and probes at the storage temperatures listed in the Reagents and Materials section.
- Always check the expiration date prior to use. Do not use expired reagents.
- Many of the reagents, including Kingfisher reagents, FAM-7-PolyU Reporter, and ROX dye, are sensitive to light exposure. Please be aware and when possible follow manufacturer's instructions for handling and storage.
- Primers, probes (including aliquots), and enzyme master mix must be thawed and kept on a cold block at all times during preparation and use.
- Do not refreeze primers, probes, and aliquoted master mixes.
- Controls and aliquots of controls must be thawed and kept on ice at all times during preparation and use.

Specimen Collection, Handling, and Storage

Specimen collection

- The assay will be performed on nasopharyngeal (NP) samples collected using a nylon flocked swab placed in 3 mL universal transport medium (UTM) tubes
- Patient identification/labeling: Patient specimens must be labeled with at least two patient identifiers (e.g. name, date of birth, medical record number, or social security number.) The information on the sample and requisition must be complete and match. (Refer to the MGH-Microbiology Specimen Identification Policy)

Specimen transport

- Samples should be transported at 2-8°C. Specimens placed in the transport medium following collection can be stored for up to 72 hours at 2-8°C. If a delay in testing or shipping is expected, store specimens at ≤ –70°C or below

Specimen storage

- Upon processing the specimen, make an aliquot of the specimen to be saved in the ≤ –70 °C freezer:

A) Label one 2 mL microcentrifuge tube with the appropriate specimen labels

B) Pipet 1.5 mL of the specimen from the UTM container after vortexing into the labeled microcentrifuge tube

C) Aliquots are saved in a ≤ –70 °C freezer

Storing Purified Specimen Nucleic Acid

- Store purified nucleic acids in a ≤ –70 °C freezer

Reagent, Controls, and Equipment Preparation

Preparation of the mCARMEN-RVP reagents and equipment is broken down into 7 steps:

1. Preparation of primer pools
2. Preparation of crRNA and Cas13 working stocks
3. Preparation of Sample Master Mix
4. Preparation of Assay Master Mix
5. Preparation of controls
6. Preparation of equipment
7. Preparation of Sample Assignment Sheet

Note: In many cases, various combined reagents are listed below in excess of their exact needs, to account for pipetting and aliquoting errors. This includes the Sample Master Mix, the Assay Master Mix predicate, the Binding Bead Mix and RT-PCR Master Mix, and the individual aliquots of each primer pool**.**

1. Preparation of primer pools (Target-free, pre-amplification space, MGH Area I) :

● Upon receipt, pool the primers into two separate pools (one forward, one reverse), according to *SOP Table 6: Primer pools preparation* below, using the following steps:

A. Normalize all primers to 100 µM with nuclease-free water

B. Pool to a working solution of 5 µM of each virus-specific primer and 1.67 µM RNase P primer

C. Split the pools into 4 700 µL aliquots into pre-labeled tubes

D. Store the primer pools and individual 100 µM primer stocks at -20°C

● Note: for one target (HRSV) there are 2 reverse primers. This is to account for the viral sequence diversity of HRSV.

SOP Table 6: Primer pools preparation

Forward Primer Pool	
**Primer**	**Amount (µL)**
SARS-CoV-2	140
FLUAV	140
FLUBV	140
HMPV	140
HRSV	140
-	-
HPIV-3	140
HCoV-OC43	140
HCoV-HKU1	140
HCoV-NL63	140
RNase P	46.6
water	1493.4
**Total**	**2800**

Reverse Primer Pool	
**Primer**	**Amount (µL)**
SARS-CoV-2	140
FLUAV	140
FLUBV	140
HMPV	140
HRSV-R1	140
HRSV-R2	140
HPIV-3	140
HCoV-OC43	140
HCoV-HKU1	140
HCoV-NL63	140
RNase P	46.6
water	1353.4
**Total**	**2800**

2. **Preparation of crRNA and Cas13 working stocks (Target-free, pre-amplification space, MGH Area I)**

crRNA

- Upon receipt, individually resuspend the 10 lyophilized crRNAs in nuclease-free water to 100 µM
- From the resuspensions, make 1 µM working stocks of each crRNA using nuclease-free water
- Store all crRNA stocks at ≤ -70°C

Cas13

- Upon receipt of Cas13, adjust working concentration with nuclease-free water to be 0.55 mg/mL
- Store all Cas13 aliquots at ≤ -70°C

3. Preparation of Sample Master Mix (Target-free, pre-amplification space)

Note: Mix all reagents listed in *SOP Table 7: 10x Cleavage Buffer,* in a 15 mL tube. Mix all reagents listed in *SOP Table 8: Sample Master Mix for 20 reactions,* in a 50 mL tube (use the cold block for cortical tubes)

A. Make 10x Cleavage Buffer per *SOP Table 7: 10x Cleavage Buffer* below

B. Aliquot 1 mL of 10x Cleavage Buffer into 10 pre-labeled nuclease-free, sterile tubes (additional aliquots of 10x Cleavage Buffer can be stored at ≤ -70°C)
C. Make Sample Master Mix per *SOP Table 8: Sample Master Mix for 20 reactions,* below
D. Aliquot 1480 µL of Sample Master Mix into 20 pre-labeled nuclease-free, sterile tubes
E. Wrap each tube with parafilm then aluminum foil to protect from light. Store at ≤ -70°C

SOP Table 7: 10x Cleavage Buffer

	**Reagent**	**Input (mL)**
1	1M Tris-HCl (pH 7.5)	4
2	0.1M DTT	1
3	Nuclease-free H2O	5
	Total	10

SOP Table 8: Sample Master Mix for 20 reactions*

*(each reaction contains 96 samples)

	**Component**	**1 sample (µL)**	**1920 samples (µL)**
**1**	Nuclease-free water	8.6485	20756.4
**2**	10x Cleavage Buffer	1.4	3360
**3**	NEB rNTPs	0.56	1344
**4**	RNase Inhibitors	0.7	1680
**5**	Loading Reagent (20x)	0.7	1680
**6**	FAM-7-PolyU Reporter (16 uM)	0.4375	1050
**7**	ROX dye (50x)	0.028	67.2
	**Total**	**14**	**29937.6**

4. Preparation of Assay Master Mixes (Target-free, pre-amplification space, MGH Area I)

A. Mix Assay Master Mix predicate per *SOP Table 9: Assay Master Mix predicate preparation for 20 reactions*, below in a 5 mL nuclease-free tube on a cold block
B. Aliquot 5.98 µL to Assay Master Mix predicate into each well of 20 12-well strip tubes

○ A single 12-well strip tube is needed per IFC

C. For each 12-well strip tube, make final Assay Master Mixes (using a total of 10 crRNAs) by adding 4.42 µL of 1 µM crRNA to wells containing Assay Master Mix predicate (1 crRNA per well, according to *Figure 1: Assay Master Mix set up in 12-well strip tubes,* below)

D. 2 wells will not receive crRNA, so should receive 4.42 ul of nuclease-free water. These are used as No crRNA controls (no-crRNA)

E. Store Assay Master Mixes and crRNAs at ≤ -70°C

F. **ON DAY OF USE**: add 10.4 µL of 2x Assay Reagent (from Fluidigm 192.24 GE DYNAMIC ARRAY kit) to each Assay Master Mix before usage (see Detection section, below)

SOP Table 9: Assay Master Mix predicate preparation for 20 reactions

	**Component**	**1 IFC experiment (µL)**	**20 IFC experiments (µL)**
**1**	**Nuclease-free water**	21.20	511.98
**2**	**Cas13a working stock**	14	338.1
**3**	**Lucigen T7 pol**	38.40	927.36
	**TOTAL**	**73.6**	**1777.44**

Figure 1: Assay Master Mix set up in 12-well strip tubes

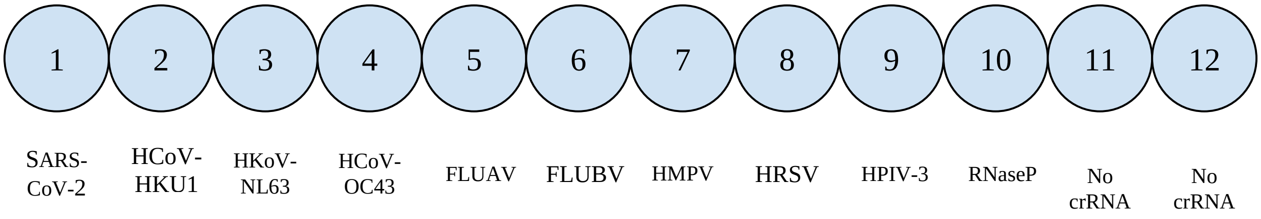

5. Preparation of controls

The mCARMEN-RVP protocol needs the following controls: extraction negative control (EC), no template control (NTC), combined positive controls (CPC), negative detection control (NDC), and no crRNA control (no-crRNA). Prepare each as follows below (a 6th control, RNase P, is included with the viral target primer and crRNA sets):

Extraction Control

1. Extraction negative Control (EC)

● Pool thirty nasopharyngeal swab specimens that have been tested negative for all the viruses on the panel by mCARMEN.

● Aliquot into 200 µL volumes and store at ≤ -70°C.

● Extract EC with extraction method per this Standard Operating Protocol alongside each batch of clinical specimens.

RT-PCR Controls

2. No Template Control (NTC):

● Sterile, nuclease-free water

● Aliquot in small volumes

● The NTC is used to check for contamination during specimen extraction, RT-PCR and detection plate set-up

3. Combined Positive Control (CPC):

● Pool *in vitro* transcribed synthetic RNA of all the targets on the panel to 1,000 copies/µL (see *Appendix 3: In vitro transcription of mCARMEN-RVP gene fragments for LoD testing, contrived sample evaluation, and CPC)*

● Aliquot in 10 µL and store at ≤ -70°C.

● Amplify CPC with amplification method per RT-PCR Standard Operating Protocol alongside each batch of extracted clinical specimens

Detection Controls

4. Negative Detection Control (NDC)

● The NDC uses only the Sample Master Mix prior to the addition of $MgCl_2$ (see below)

● This is a negative control for the Fluidigm detection step and should yield a negative result for all targets on the panel

5. No crRNA control (no-crRNA):

● Already made in Assay Master Mix step above

● This is a negative control for the Fluidigm detection step and should yield a negative result for all targets on the panel

6. Preparation of equipment

Clean and decontaminate all work surfaces, pipettes, centrifuges, and other equipment prior to use. Decontamination agents should be used including 5% bleach, 70% ethanol, and RNase AWAY® to minimize the risk of nucleic acid contamination.

7. Preparation of Sample Assignment Sheet

Prior to running a mCARMEN-RVP experiment, determine the sample set you will be working from, and fill in this template with the sample IDs.

Note: no cells should be empty. If there are not samples in given wells, they should be given the label “empty” or “null”

Detailed Assay Protocol

With the previous preparations on hand, the protocol itself can be broken down into six distinct steps:

1. Nucleic Acid Extraction
2. RT-PCR master mix preparation and RT-PCR amplification
3. Preparation of Cas13 Detection reactions and Sample Master Mix plate
4. Priming and loading the Fluidigm IFC
5. Running the IFC on the IFC Controller
6. Running and imaging the IFC on the Fluidigm Biomark

1. Nucleic Acid Extraction

In this step you will extract viral RNA from patient samples using the MagMAX Viral/Pathogen Nucleic Acid Isolation Kit on the KingFisher Flex Magnetic Particle Processor with 96 Deep-Well Head. This extraction method directly follows the EUA-Approved Applied Biosciences TaqPath COVID-19 Combo Kit instructions for the automated extraction of 200 uL sample input volume, with the single exception of leaving out the MS2 phage control.

Reagents (except for nuclease-free water) are found in the MagMAX Viral/Pathogen Nucleic Acid Isolation Kit. Follow the “Extract RNA - Automated method (200-µL sample input volume)

A. Prepare Wash 1, Wash 2, and Elution plates according to *SOP Table 10: Isolation plate set up*, below
B. Prepare Binding Bead Mix according to the *SOP Table 11: Binding Bead Mix preparation,* below, with reagents from MagMAX™ Viral/Pathogen Nucleic Acid Isolation Kit
C. Prepare Sample Plate as follows:
1. Pipette 5 µL of Proteinase K to each well in the KingFisher™ Deepwell 96 Plate (Label as "Sample Plate")
2. Add 200 µL of sample to each sample well, according to the Sample Assignment Sheet (above)
3. Add 200 µL of nuclease-free water (not DEPC-Treated) to the Negative Control well
4. Add 200 µL of negative patient sample to Extraction Control well
5. Invert the Binding Bead Mix (prepared above in Step B) 5 times gently to mix, then add 275 µL to each sample well, the Extraction Control well, and Negative Control well in the Sample Plate

Notes

- Remix Binding Bead Mix by inversion frequently during pipetting to ensure even distribution of beads to all samples or wells.

- Binding Bead Mix is viscous, so pipet slowly to ensure that the correct amount is added.
- DO NOT reuse pipette tips to add Binding Bead Mix to the samples, as the high viscosity will cause variations in the volumes added.

D. Load the Sample Plate onto the KingFisher instrument according to the manufacturer's recommended procedures.
E. Run the following protocol on the KingFisher instrument available from the ThermoFisher website: MVP_2Wash_200
F. After the extraction has finished, properly dispose of the Wash and Samples Plates, but keep the elution plate on ice or store at ≤ -70°C until proceeding to Step 2 RT-PCR amplification.

SOP Table 10: Isolation plate set up

Plate ID	Plate position	Plate type	Reagent	Volume per well (μL)
Wash 1	2	KingFisher Deepwell 96 Plate	Wash Buffer	500
Wash 2	3	KingFisher Deepwell 96 Plate	80% Ethanol	1,000
Elution	4	KingFisher Elution Plate	Elution buffer	50
Tip Comb	5	Place a KingFisher 96 tip comb in a KingFisher Elution Plate (this plate exists only to hold the tip comb in place)		

SOP Table 11: Binding Bead Mix preparation

Component	Volume per well (μL)	Binding Bead Mix 96 samples
Binding Solution	265	31800
Total Nucleic Acid Magnetic Beads	10	1200
**Total volume per well**	**275**	**33000**

2. RT-PCR master mix preparation and RT-PCR amplification

In this step you will prepare the RT-PCR master mix and amplify the target/sample RNA with the Qiagen OneStep RT-PCR kit.

Things to do before you start

- Clean the bench using 10% bleach, RNase AWAYspray, and 70% ethanol
- Clean an ice bucket with bleach, RNase AWAY and 70% ethanol and fill with ice
- Thaw Qiagen 5x OneStep RT-PCR Buffer, dNTPs, and forward and reverse primer pools on ice
- Prepare one 96-well plate; Label the plate with the following information:
  - RTP-Experiment Number
  - Sample Plate
  - Date of experiment

RT-PCR master mix preparations

This step requires the following reagents/kit components:

- Forward and Reverse Primer Pools (*SOP Table 6: Primer pools preparation,* above)
- QIAGEN OneStep RT-PCR Kit
  - 5x OneStep RT-PCR Buffer
  - dNTP mix
  - Enzyme mix
- Sample RNA (from Kingfisher Extraction, Detailed Assay Protocol Step 1 above)

1. In a target-free area, mix the RT-PCR Master Mix according to *SOP Table 12: RT-PCR Master Mix preparation*, below, in a sterile nuclease-free 5 mL tube.
2. Aliquot 40 µL of the RT-PCR Master Mix into the pre-labeled 96-well plate
3. Add 10 µL of water to the amplification negative control well.
4. Seal the plate before transferring to a designated area for working with patient sample RNA.
5. Add 10 µL of the RNA sample elution from the KingFisher Elution Plate to the 96-well plate containing the RT-PCR Master Mix.
6. Add CPC (combined positive control) to the positive control well
7. Seal the plate
8. Vortex the plate
9. Spin down the plate in a centrifuge
10. Load the plate onto the thermocycler
11. Run the cycling conditions listed in *SOP Table 13: Amplification conditions,* below
12. After the reaction has finished, proceed to Step 3 or store reactions at -20°C

SOP Table 12: RT-PCR Master Mix preparation

Reagent	1 sample (µL)	96 samples (µL)
5x OneStep RT-PCR Buffer	12.5	1380
Forward Primer Pool (5 µM)	3	331.2
Reverse Primer Pool (5 µM)	3	331.2
dNTP mix (10 mM each)	2	220.8
Qiagen enzyme Mix	2	220.8
Sample (KingFisher Elution Plate)	10	-
Nuclease-free water	17.5	1932
**Total**	**50**	**4416**

SOP Table 13: Amplification conditions

Step	Temp	Time
Reverse Transcription	50	30 min
	95	15 min
Amplification x40 cycles	94	30 s
	58	30 s
	72	30 s
End	4	hold

3. Preparation of Cas13 Detection reactions and Sample Master Mix plate

In this step you will prepare the Sample Master Mix plate and the Assay Detection reactions.

Things to do before you start

- Clean the bench using 10% bleach, RNase AWAY spray, and 70% ethanol
- Clean an ice bucket with bleach, RNase AWAY and 70% ethanol and fill with ice.
- Thaw one or two pre-made aliquot(s) of Sample Master Mix (1480 µL per 96 samples, *SOP Table 8: Sample Master Mix for 20 reactions*, above) and one 12-well strip tube of Assay Master Mixes (10.4 µL per well, *SOP Table 9: Assay Master Mix predicate preparation for 20 reactions*, above) on ice
- Prepare one or two 96 well plates for the Sample Master Mix plate(s) by labeling the plates with the following information:
  - Sample Plate (1 or 2)
  - Date of experiment

This step requires the following reagents/kit components:

- Sample Master Mix (see above, *SOP Table 8: Sample Master Mix for 20 reactions*)
- Assay Master Mixes (see above, SOP *SOP Table 9: Assay Master Mix predicate preparation for 20 reactions*)
- Amplified RT-PCR product (see above, Detailed Assay Protocol Step 2)
- $MgCl_2$ (1M stock concentration)
- Fluidigm 2x Assay Loading Reagent

Prepare Cas13 Detection Reactions

- Thaw the Assay Master Mixes on ice
- Make the Cas13 Detection Reactions by adding 10.4 µL of 2x Assay Loading Reagent to each Assay Master Mix well, vortex and spin down prior to proceeding to Step 4

Prepare Sample Master Mix Plate

1. Open an aliquot of Sample Master Mix (prepared above according to *SOP Table 8: Sample Master Mix for 20 reactions*. A single aliquot is enough for 96 samples.)
2. Add 12.6 µL of this Sample Master Mix in the appropriate well for the Negative Detection Control (NDC) - see Sample Assignment Sheet
3. Add 15.12 µL 1M $MgCl_2$ to the remaining Sample Master Mix and vortex
4. Transfer the pre-made Sample Master Mix plus $MgCl_2$ mixture to a 5 mL reservoir
5. Pipet 12.6 µL of this mixture into the pre-labelled Sample Master Mix Plate using a multi-channel pipette
6. Seal the Sample Master Mix Plate with aluminium foil seal to protect from light
7. Transfer the Sample Master Mix Plate to an area for handling high-copy, amplified material
8. Remove aluminium foil seal from Sample Master Mix Plate
9. Add 1.4 µL of amplified target sample product (from Detailed Assay Protocol Step 2 above) per well, following the Sample Assignment Sheet
10. Seal the Sample Master Mix Plate with the added amplified target using an aluminium foil plate seal
11. Vortex the sealed Sample Master Mix Plate
12. Spin the Sample Master Mix Plate down in centrifuge at top speed 15 seconds
13. Keep the Sample Master Mix Plate on ice before proceeding to Step 4

4. Priming and Loading the Fluidigm IFC

In this step you will load the samples from the Sample Master Mix plate, the Cas13 Detection Reactions, and various other fluidics reagents into the Fluidigm 192.24 Integrated Fluidics Circuit (IFC).

Things to do before you start

- Clean the bench and an ice bucket using 10% bleach, RNase away spray, and 70% ethanol
- Thaw Fluidigm GE IFC fluidic reagents: Actuation and Pressure Fluid on ice
- Refer to the following figure when priming and loading the IFC

Figure 2 - Fluidigm Integrated Fluidics Circuit (IFC)

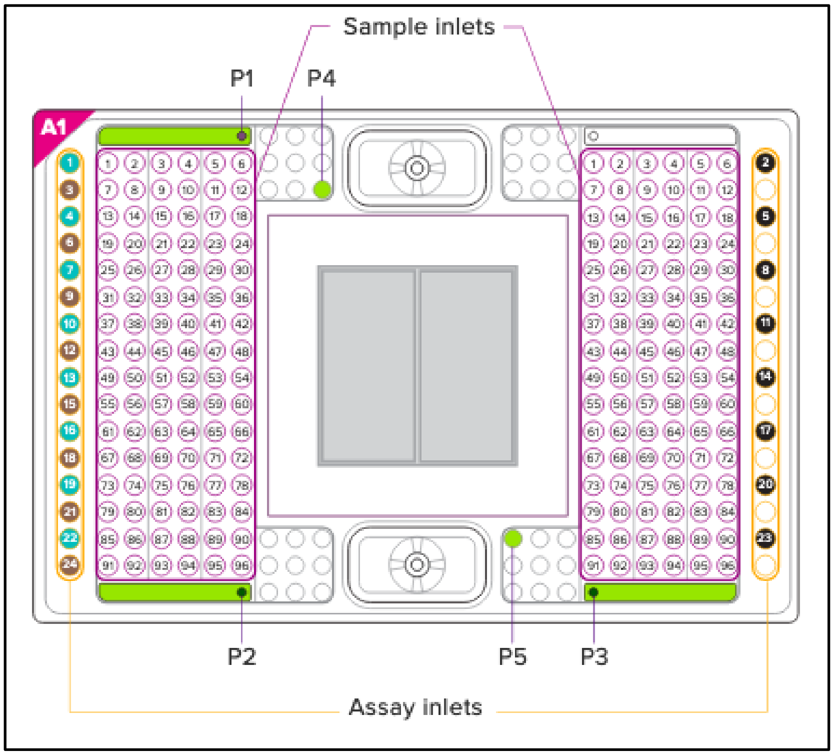

IFC priming and loading

- The barcode is always on the left
- Assay Detection reactions go into the leftmost and rightmost columns (Assay inlets)
- Sample Master Mix Plate 1 goes on the left side Sample inlets
- Sample Master Mix Plate 2 goes to the right side Sample inlets

1. Open the IFC 192.24

2. Loading the control line fluid (single control line fluid syringe in Fluidigm Kit)
a. Tap to remove bubbles
b. Pull back at bit on the plunger (now there should be no bubble left)
c. Remove black cap
d. Don't eject air until fluid is at the top

e. Inject into one of the hole on the top center piece with a spring and push down the black gasket, so that the fluid can enter into the chamber

f. Push fluid into chamber

■ Only 1 control line syringe is needed per IFC

3. Addition of Fluidigm Biomark Gene Expression (GE) IFC fluidic reagents

a. Pipette 150 µL of Actuation Fluid into the P1 well on the IFC

b. Pipette 150 µL of Pressure Fluid into the P2 and P3 wells on the IFC

c. Pipette 20 µL of Pressure Fluid into the P4 and P5 wells on the IFC

4. Addition of Cas13 Detection Reactions

■ Handle these on the left side of the bench

■ When pipetting into the IFC, hold the multichannel straight up and down and make sure the tips go all the way to the bottom of the wells; You want the liquid to dispense evenly in the bottom of the IFC wells (not stuck on the edges) with no bubbles

■ Only pipette up to the first stop on the pipettor. Don't go to the second stop to avoid bubbles

a. Add 4 µL of the Cas13 Detection Reactions into the appropriate assay inlets (1 per inlet)

■ Load the 12 Assay Detection Reactions 2x to fill all 24 Assay inlets

b. Place Assay Detection Reactions back on ice

5. Addition of samples from Sample Master Mix Plate(s)

■ Handle these on the right side of the bench

■ Sample Master Mix Plate 1 goes on the left side of the IFC and Sample Master Mix Plate 2 goes on the right side of the IFC Set up the bench so that the waste bucket is not near the IFC

b. Clean the multichannel and plate sealer

c. Open the Sample Master Mix Plate. Immediately discard the plate sea and gloves

d. Put on fresh gloves.

e. Load 4 µL each into the appropriate sample wells

5. Running the IFC on the IFC Controller

In this step, you will place the previously prepared IFC (Step 4, aboe) onto the Fluidigm Controller (HX) hardware and mix the reactions.

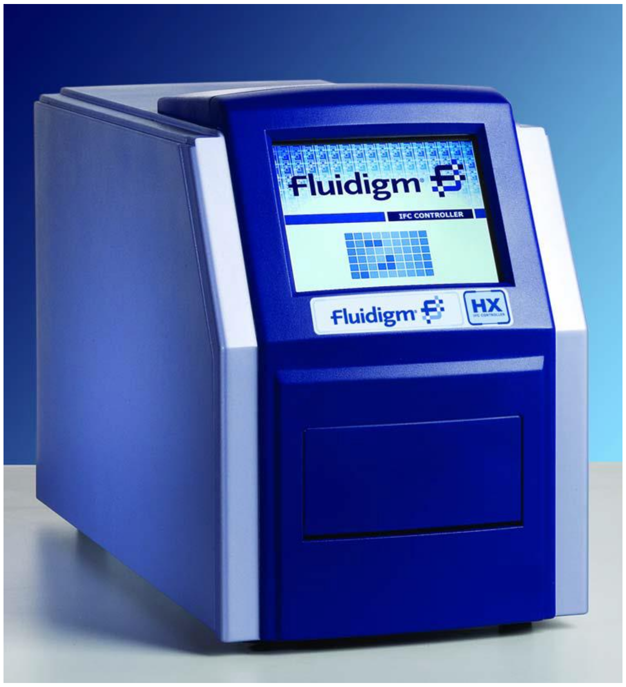

1. Remove the sticker from the IFC
2. Place the IFC inside the IFC controller with the barcode side in front
3. Click “Load and Mix 192.24 IFC”
4. Allow the run to proceed. After ~30 minutes the run should complete.
5. Take out the IFC and proceed immediately to Step 6

6. Running and imaging the IFC on the Fluidigm Biomark

In this step, you will place the IFC into the Fluidigm Biomark, image the reactions and export the experimental data. This step requires 2 separate pieces of software from Fluidigm: the Biomark Data Collection software, and the Fluidigm Real-Time PCR software.

1. Imaging the IFC on the Biomark:

a. Open the Biomark Data Collection software

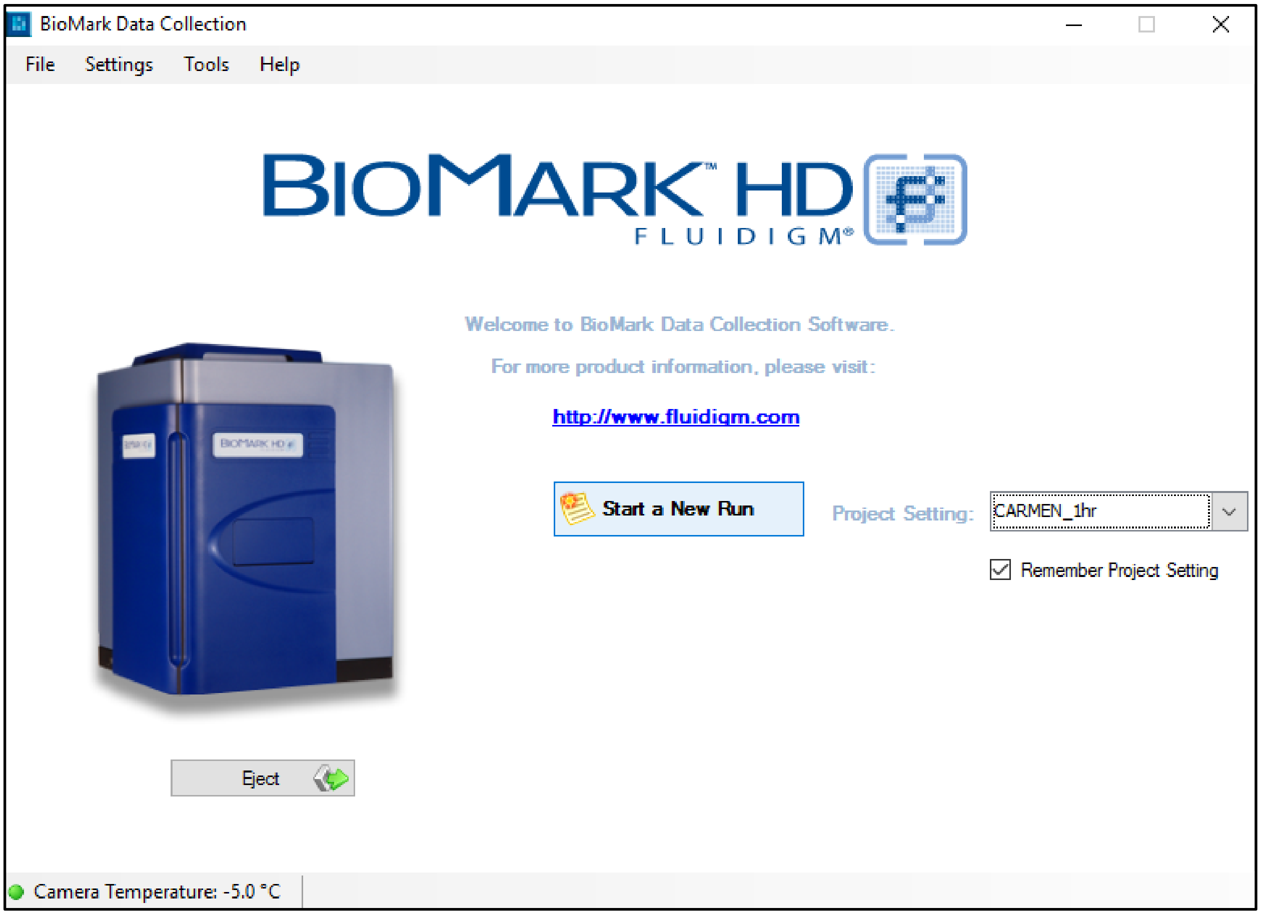

b. Place the IFC into the instrument with the "barcode"-site in front

c. Choose the following project setting: mCARMEN 192 SNPtype-FAM

i. If not pre-installed, download setting file here

d. Click "Load", wait for the pump noise

e. On the "Chip Run" screen, Name the Run and select the data folder location

f. On the "Application: Reference and Probes" screen, set/confirm the following settings:

i. Application: Gene expression

ii. Passive Reference: ROX

iii. Assay: Single Probe

iv. Probes: SNPtype-FAM

g. On the "Thermal Protocol" screen, set/confirm the following settings:

i. Protocol File: From Project Setting

ii. Auto exposure checked

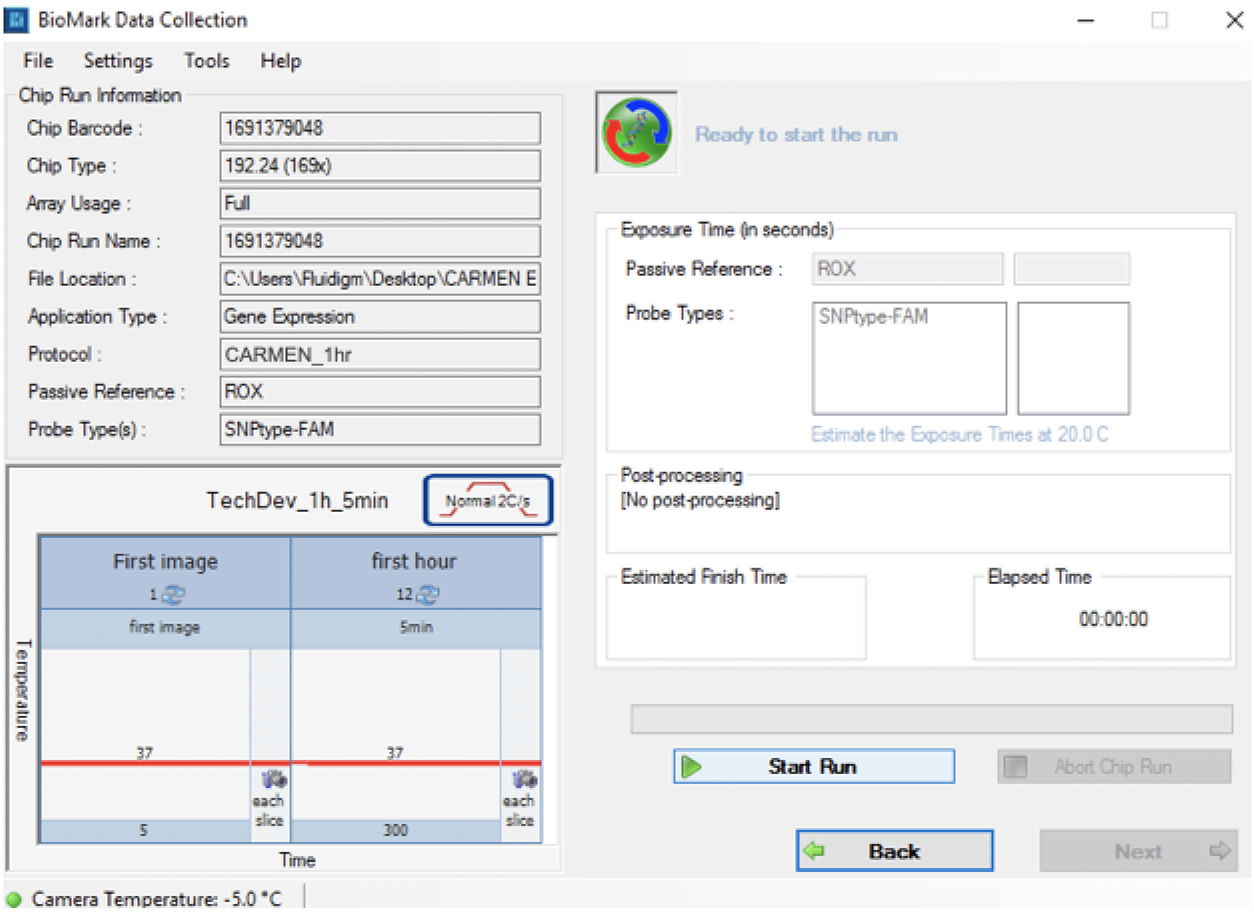

h. Click “Start Run”

i. After the run has finished, discard the IFC into a biohazard waste container

2. Exporting data from the Biomark run:

a. Open the Fluidigm Real-Time PCR software

b. Export the raw data file as follows:

i. File > Open

ii. Select the file that contains your experiment

iii. Once the file has loaded, select File > Export

iv. In the drop down menu select Save as type > Table Results with Raw Data (*.csv) then save

3. Proceed to the Data Analysis Using mCARMEN-RVP Software Section with the Fluidigm Biomark raw data file (.csv)

Data Analysis Using mCARMEN-RVP Software

To analyze the data coming off of the Fluidigm Biomark for the mCARMEN-RVP, you will need to download and install the mCARMEN-RVP Software, available here. Please follow installation instructions per the software documentation.

For each Fluidigm IFC that will be analyzed, you will need the following 2 data files:

- **Fluidigm Biomark raw data file (.csv):** See above
- **Sample Assignment Sheet (.xlsx) -** See above

Launch the mCARMEN-RVP Software:

1. If installation is needed, download the mCARMEN-RVP Software from github and follow installation instructions in the software documentation

2. Open terminal (Mac) or command line (PC)

3. To launch the software type:

a. PC: $ python ./src/carmen_rvp_analysis.py

b. Mac: $ pythonw ./src/carmen_rvp_analysis.py

4. After a few moments the software should open in a separate window. See below for reference.

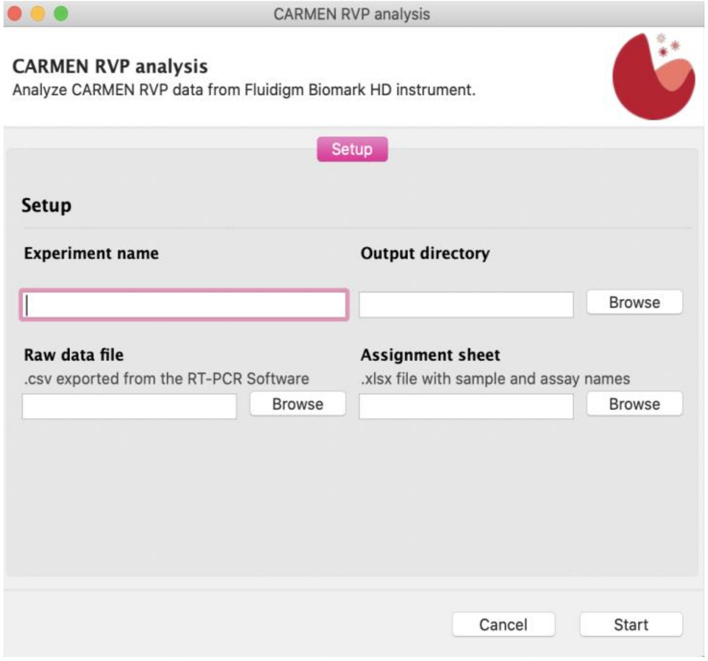

5. With the software open:

a. Assign an Experiment Name

b. Choose the output directory where the analysis files will be saved

c. Upload the Fluidigm Biomark raw data file (.csv)

d. Upload the Sample Assignment Sheet (.xlsx)

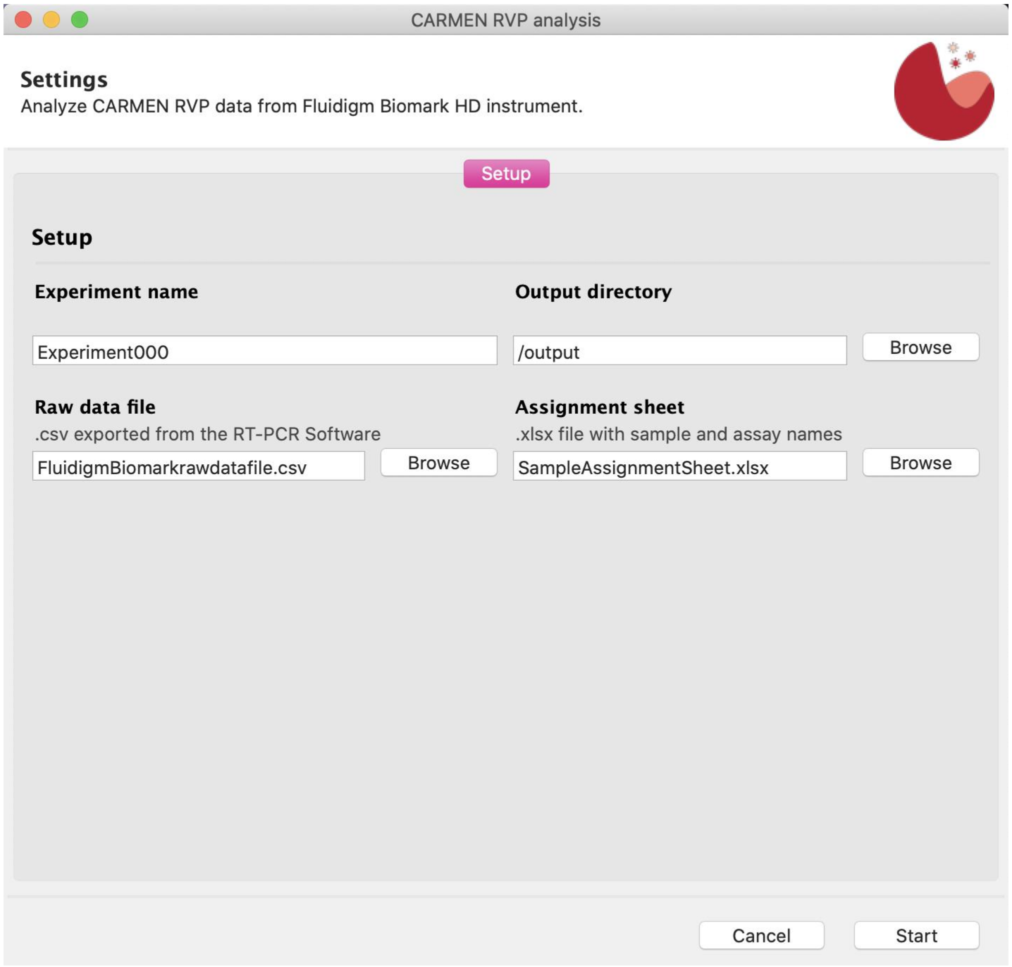

6. Press the ‘Start’ Button and allow the software to run:

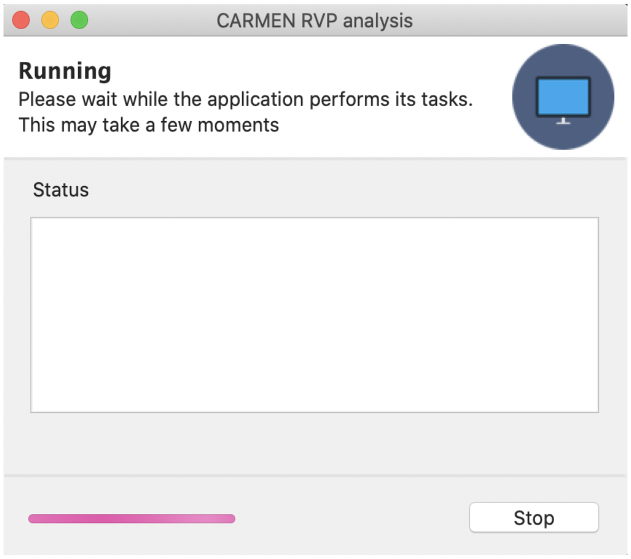

7. Once finished, navigate to the output folder to view analysis files

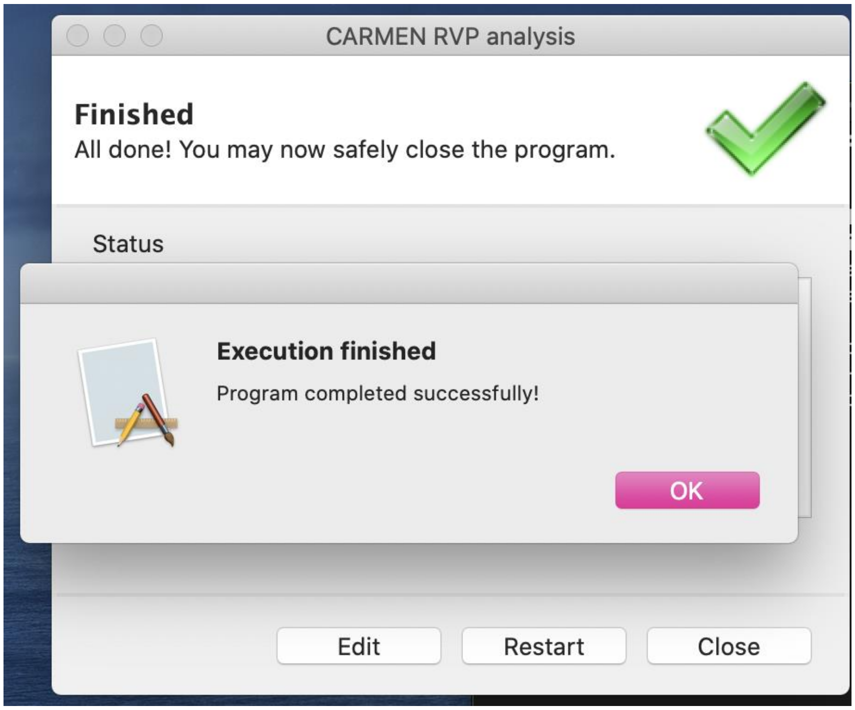

Upon completion, the analysis software will produce the following output files:

1. [EXPERIMENT NAME]_heatmap_t12.png
2. [EXPERIMENT NAME]_HitQuantification.csv
3. [EXPERIMENT NAME]_hits.csv
4. [EXPERIMENT NAME]_intial_raw_hitcalling_heatmap_t12.png
1. [EXPERIMENT NAME]_logfile.log
2. [EXPERIMENT NAME]_signal.csv

Of these files, the key file that captures the results of the run are:

1. [EXPERIMENT NAME]_HitQuantification.csv - This is a comma-separated list of Sample IDs accompanied by a positive, negative, or invalid determination, as defined in the chart below

Interpretation of Results and Reporting

Interpretation of Control Results

mCARMEN-RVP uses 4 process controls and 2 internal controls (see above, section 3) to monitor several different aspects of the assay, including extraction, RT-PCR, and detection. The mCARMEN-RVP Software analyzes each of the controls (as specified below) in order to determine if the controls were performed properly, using the signal threshold algorithm (see "Interpretation of Patient Sample Results" below for a description). The mCARMEN-RVP Software establishes the validity of the entire mCARMEN-RVP run by checking the process controls as outlined below. If a run is valid, the mCARMEN-RVP Software then assesses the internal controls, and makes a "detected," "not detected," or "invalid" result for each sample/viral target combination (see "Interpretation of Patient Sample Results," below).

See *SOP Table 14* below for a summary of the controls used with mCARMEN-RVP and their expected performance, and *Appendix 2: Detailed interpretation of control results* for a more detailed discussion of the controls used with mCARMEN-RVP.

SOP Table 14. Expected performance of controls.

Control Type	Used to Monitor	Expected RNase P Results	Expected no-crRNA Results	Expected Viral Target Results
**Extraction negative Control (EC)**	Failure in lysis and extraction procedure, potential contamination during extraction	Positive	Negative	All negative*
**No Template negative Control (NTC)**	RT-PCR reagent and/or environmental contamination	Negative	Negative	All negative*
**Combined Positive Control (CPC)**	Substantial reagent failure including primer, crRNA, and CRISPR-Cas13 integrity	Positive	Negative	All positive*
**Negative Detection Control (NDC)**	Sample master mix and/or environmental contamination	Negative	Negative	All negative*
**Internal Sample Controls**	RNase P and no-crRNA internal controls	Positive**†	Negative**	n/a

(*) If results for any of the 4 process controls do not exhibit expected performance for any of the viral targets, *results are invalidated only for that viral target*.

(**) denotes internal controls. If these do not exhibit expected performance, *only the individual sample results are invalidated*.

(†) If RNase P is negative for a given sample, but another viral target for the same sample produces a signal, the result is valid.. (Due to competitive amplification, a high viral titer may cause a low or absent RNase P signal.)

If results for the 4 process controls do not exhibit expected performance for either RNase P or no-crRNA controls, the *entire mCARMEN-RVP run is invalidated* and must be redone from the biosample/extraction step.

To ensure the dynamic range of the signals produced by mCARMEN-RVP is adequate for hit calling, a final process control is conducted by comparing the signals from CPC and NDC. See *Appendix 2: Detailed interpretation of control results* for discussion of this step.

If any of the above controls do not exhibit the expected performance as described and the mCARMEN-RVP Software reports an invalid result, the assay may have been improperly set up and/or executed improperly, or reagent or equipment malfunction could have occurred. Please see *Appendix 2: Detailed interpretation of control results,* for details of control outcomes.

Interpretation of Patient Sample Results
Detection of a viral target in a sample depends on the viral target specific sample signal compared to the NTC. The determination of results from the mCARMEN-RVP is conducted automatically by the mCARMEN-RVP Software.

A viral target is **detected** in a sample if the sample signal is at least 1.8 times higher (baseline threshold) than the respective NTC from that viral target. Otherwise, it is **not detected**. Multiple viruses may be detected in a single sample.

Ratio = Sample Signal / No Template Control (NTC)
- If the ratio is >= 1.8 then the viral target for that sample is called "detected"
- If the ratio is < 1.8 theviral target for that sample is called "not detected"

Viral Gene Targets

If all process controls exhibit the expected results as defined above, the results for the samples can be interpreted as follows:

a. If one (or more) of the viral targets for a given sample generates signal above the 1.8x threshold, the sample is considered detected for those viruses
- Multiple viruses may be detected in a single sample
- The RNase P reaction may or may not be positive as described above, but the viral target results are still valid if at least one of them is above the threshold

b. If all viral targets for a given sample *do not* generate signal above the 1.8x threshold BUT the RNase P for that sample *does* exceed the 1.8x threshold, the sample is considered not detected for all viral targets

c. If the signals of the viral targets for a given sample *do not* meet or exceed the 1.8x threshold AND the RNase P for the same sample *does not* meet or exceed the 1.8x threshold, the results *for that sample* are invalid

In the case of invalid results, repeat mCARMEN-RVP for the invalid sample from the biosample/extraction step. If the sample remains invalid upon retest, collection of a new specimen and subsequent testing should be considered.

Sample Result	Definition of Result
Positive / +	Positive result. The viral RNA is present in the sample, above the predetermined threshold for positivity.
Negative / -	Negative result. The signal falls below the predetermined threshold for positivity.
Invalid / !	Invalid assay result. This is usually due to an issue with one or more control (see below)
Invalid / -2	A control only result. A -2 points out the *causative* controls that invalidated one or more assays in the run.

Appendix 1: mCARMEN-RVP Primers, crRNA, and Reporter sequences

Primers are sourced from Eton, crRNA and Reporter a sourced from IDT

Table A1: mCARMEN-RVP Primers, crRNA, and Reporter sequences

	Virus	Primer	Oligonucleotide Sequence (5'-3')	Target Gene
1	SARS-CoV-2	Forward primer	GAAATTAATACGACTCACTATAGGGCAATTAGAGATGGAACTTACACC	Orf1ab
		Reverse primer	CTTTTTAGCTTCTTCCACAATGTC	
		crRNA	GAUUUAGACUACCCCAAAAACGAAGGGGACUAAAACCUAAAACUAUUCACUUCAAUAGUCUGAA	
2	FLUAV	Forward primer	GAAATTAATACGACTCACTATAGGGGCGTGTTGATGAGAACGG	PB1
		Reverse primer	GCCACAAACTTACAATCTTTTACAC	
		crRNA	GAUUUAGACUACCCCAAAAACGAAGGGGACUAAAACUGGUGUAUUUCUUUUGUCCAAGAUUCAG	
3	FLUBV	Forward primer	GAAATTAATACGACTCACTATAGGGGGTGGTATGAGCTACTTTTGTGTA	PB1
		Reverse primer	ACTACCTGTGCACATATTCTTGTATA	
		crRNA	GAUUUAGACUACCCCAAAAACGAAGGGGACUAAAACUCCAAUGUUUUUGAUGCCUAGUGCUGCU	
4	HRSV	Forward primer	GAAATTAATACGACTCACTATAGGGCTTCACGAAGGCTCCACATA	M
		Reverse primer-1	CCCATATTGTTAGTGATGCAGG	
		Reverse primer-2	GCACCCATATTGTTAGTGATGC	
		crRNA	GAUUUAGACUACCCCAAAAACGAAGGGGACUAAAACGUCUUUUUCUAGGACAUUGUAUUGAACA	
5	HMPV	Forward primer	GAAATTAATACGACTCACTATAGGGACCCAAATGAGAAAGACTGTG	F
		Reverse primer	GCAACATTAATTCCTGCTGCT	
		crRNA	GAUUUAGACUACCCCAAAAACGAAGGGGACUAAAACGUCGCAAAAGACAUGGUCUCCUCUUGUU	

6	HPIV-3	Forward primer	GAAATTAATACGACTCACTATAGGGACGAATCAAAGATAAATACGGGAG	M
		Reverse primer	ATTGGTAATGATCCAGAGCCA	
		crRNA	GAUUUAGACUACCCCAAAAACGAAGGGGACUAAAACUUGUAACUCGGGUCACUGUCAAGAUCAU	
7	HCoV-HKU1	Forward primer	GAAATTAATACGACTCACTATAGGGTTCTGGTAGTGGTCAGGCTA	Orf1ab
		Reverse primer	CTTATAGGGTCATTTGTACCTATAGG	
		crRNA	GAUUUAGACUACCCCAAAAACGAAGGGGACUAAAACCGCAUAACAAAUCGUGAAUCACGAACUA	
8	HCoV-NL63	Forward primer	GAAATTAATACGACTCACTATAGGGGTGACTTTGATATTGTAGTGGCTT	Orf1ab
		Reverse primer	ATACCACAAATAGTAGCTATAGTCTGC	
		crRNA	GAUUUAGACUACCCCAAAAACGAAGGGGACUAAAACUGUGCCACAACAAGAAGAUAAGUAACCA	
9	HCoV-OC43	Forward primer	GAAATTAATACGACTCACTATAGGGTTCTGAGAGTTGGAGTGTTGG	RdRp
		Reverse primer	ACCAGGAACAACACAAAGTTTC	
		crRNA	GAUUUAGACUACCCCAAAAACGAAGGGGACUAAAACUCAGAUCUGUCCUCUUAACAACAAAGAA	
10	RNase P control	RNase P forward primer	GAAATTAATACGACTCACTATAGGGTTGATGAGCTGGAGCCA	RNase P
		RNase P reverse primer	ATGTGGATGGCTGAGTTGTT	
		RNase P crRNA	GAUUUAGACUACCCCAAAAACGAAGGGGACUAAAACUCCGAGUCAGUGGCUCCCGUGUGUCGGU	

Appendix 2: Detailed interpretation of control results

Process Controls - unexpected behavior can invalidate either an entire run, or results from specific viral target(s)

1. Extraction negative Control (EC)

The EC is used as an RNA extraction procedural control to demonstrate successful recovery of nucleic acid, extraction reagent integrity, and as a control for cross-contamination during the extraction process.

- The EC consists of a single, confirmed negative patient sample
- The EC should be carried through from extraction through detection
- If the EC is detected for the RNase P target AND not detected in the no-crRNA control AND the EC is not detected for all viral targets, in a given run, this confirms the success of the extraction step the run is valid with respect to the extraction step
- If the EC is not detected in the RNase P target OR detected in the no-crRNA control, in a given run, *this will invalidate the entire run*
  - This indicates a potential problem with the extraction process
  - Extraction, RT-PCR and detection must be repeated for all samples that were extracted alongside the failed EC
- If the EC is detected in any of the viral targets in a given run, this may be an indication of possible cross-contamination during extraction, RT-PCR or detection reaction set-up
  - In this case, *only the results from samples for the EC detected viral target are invalid*, and need to be repeated

2. No Template Control (NTC)

The NTC is used as a control for the integrity of RT-PCR reagents and cross-contamination during the amplification process. To observe a detected result in the NTC reaction, a signal from a viral target must be above three standard deviations from the mean of all the NTC signals from all viral targets and RNase P (with the exception of the NTC signal from the no-crRNA control (see below.))

- The NTC consists of molecular-grade nuclease-free water in RT-PCR reaction instead of RNA
- If the NTC is not detected in the RNase P target AND not detected in the no-crRNA AND not detected in any viral targets, this indicates that the RT-PCR amplification and detection reagents are not contaminated, and the run is valid with respect to the RT-PCR
- If the NTC is detected in either the RNase P target OR the no-crRNA target, in a given run, *this will invalidate the entire run*
- If the NTC is detected for any of the viral targets, *only the results from samples for the NTC detected viral target are invalid*
  - This might suggest possible cross-contamination during the RT-PCR or detection reaction set-up

○ Extraction, RT-PCR and Detection need to be repeated for these viral targets

3. Combined Positive Control (CPC)

The CPC consists of all ten targets on the mCARMEN-RVP (*In vitro* transcribed gene fragments of all 9 viruses and RNase P), combined as directed in *Appendix 3: In vitro transcription of mCARMEN-RVP gene fragments for LoD testing, contrived sample evaluation, and CPC,* to serve has a control for the integrity of the RT-PCR reagents and primer sets, and to ensure amplification and detection were properly performed. To observe a detected result in the CPC reaction, a signal from a viral target must be above three standard deviations from the mean of all the NTC signals from all viral targets and RNase P (with the exception of the NTC signal from the no-crRNA control (see below.))

- The CPC is utilized alongside each batch of clinical samples after extraction, and should produce a detected result for all viral & RNase P targets in a given run
- If the CPC is detected for each of the viral and RNase P targets, this indicates that the RT-PCR and detection steps were properly performed and the run is valid with respect to the RT-PCR and detection steps
- If the CPC is not detected for any viral or RNaseP target, this indicates that there is a problem with the primer mixes or reagents used in the RT-PCR amplification, or that the detection step failed

○ In this case, only the results from samples for the not detected CPC viral target are invalid and need to be repeated

○ An invalid result is returned when a signal from one viral target outside three standard deviations from the mean CPC signal from all viral targets

○ The no-crRNA detection control (see below) should always result in a not detected outcome for the CPC

4. Negative Detection Control (NDC) - *Sample Master Mix control*

The NDC is used to test the validity and integrity of the Sample Master Mix reagents in the absence of $MgCl_2$.

- The NDC consists of using molecular-grade, nuclease-free water instead of amplified product, in the Sample Master Mix without the addition of Magnesium Chloride ($MgCl_2$)
- If the NDC produces a not detected result for all viral and RNase P targets, this suggested that the Sample Master Mix was properly set-up, and therefore the run is valid with respect to the Sample Master Mix
- If the NDC produces a detected result for any target according to the mCARMEN-RVP Software, that indicates cross-contamination may have occurred during the detection set-up

○ In this case, only the results from samples for the NDC detected viral target are invalid, and need to be repeated

Internal Controls - evaluated only after all process controls have exhibited expected behavior

1. **No crRNA Control (no-crRNA)** - *Assay master mixes control*

The no-crRNA control is used to test the validity of the Assay Master Mix predicates, and as a control for crRNA cross-contamination during the Detection set-up.

- The no-crRNA control consists of the Assay Master Mix predicate with molecular-grade, nuclease-free water instead of crRNA and should produce a not detected result for all samples and controls
- If the no-crRNA control is detected for any sample, results from that sample alone are invalid and that sample should be rerun from the biosample/extraction step
- If the no-crRNA control is detected for any control, cross-contamination during the detection set-up likely occurred and the run is invalid
- If the no-crRNA control produces a not detected result for all controls and samples then the run is valid

2. **RNase P control (RNase P)**

The RNase P control is used as an internal amplification control and a verification of adequate sample collection.

- The RNase P control consists of a primer pair and crRNA set that targets the Human RNase P gene (see *Appendix 1: mCARMEN-RVP Primers, crRNA, and Reporter sequences).* The RNase P control is run alongside all the other viral targets and the no-crRNA control.
- If the RNase P control is detected for a given sample, even if no other viral targets are detected, then results from that sample are valid
- If the RNase P control is not detected for a given sample, AND no other viral targets are detected, then the results from that sample are invalid if the RNase P control is not detected for a given sample, BUT at least one other viral target is detected, these sample results are not invalidated. (Due to competitive amplification, a high viral titer may cause a low or absent RNase P signal.)

Validating CPC and NTC respectively to each other

A final process control is done by comparing the CPC signal to the NTC signals for a given run. The NTC and CPC are the ground truth for the hit calling. Since the validation of CPC and NTC depends on their group mean and standard deviation respectively, they have to be distinctively different from each other. For the mCARMEN-RVP run to be valid, the ratio of separation band to dynamic range has to be greater than 0.2. The dynamic range is defined as the difference of the NTC mean and CPC mean. The separation band is the range between the CPC mean subtracted by three standard deviations of the CPCs and the NTC mean plus three standard deviations of the NTCs.

Appendix 3: *In vitro* transcription of mCARMEN-RVP gene fragments for LoD testing, contrived sample evaluation, and CPC

DNA targets were ordered as dsDNA fragments, gblocks, from Integrated DNA Technologies (IDT) or Genewiz and *in vitro* transcribed using the HiScribe T7 High Yield RNA Synthesis Kit (New England Biolabs). Transcriptions were performed according to the manufacturer's recommendations with a reaction volume of 20 µL that was incubated overnight at 37 °C. The transcribed RNA products were purified using RNAClean XP beads (Beckman Coulter) and quantified using NanoDrop One (Thermo Scientific). For experimental validation, the RNA was serially diluted from 10^{11} down to 10^{-3} cp/µL and used as input into mCARMEN-RVP. CPC was made by pooling all *in vitro* transcribed RNA targets listed in Appendix Table A3 (below) to 1,000 copies/µL.

Table A3 : Sequences of synthetic targets for 9 viral targets and RNase P human control

Target on mCARMEN-RVP	Sequence
SARS-CoV-2	gaaatTAATACGACTCACTATAgggGAGTATGGTACTGAAGATGATTACCAAGGTAAACCTT<br>TGGAATTTGGTGCCACTTCTGCTGCTCTTCAACCTGAAGAAGAGCAAGAAGAAGATTGG<br>TTAGATGATGATAGTCAACAAACTGTTGGTCAACAAGACGGCAGTGAGGACAATCAGAC<br>AACTACTATTCAAACAATTGTTGAGGTTCAACCTCAATTAGAGATGGAACTTACACCAGT<br>TGTTCAGACTATTGAAGTGAATAGTTTTAGTGGTTATTTAAAACTTACTGACAATGTATAC<br>ATTAAAAATGCAGACATTGTGGAAGAAGCTAAAAAGGTAAAACCAACAGTGGTTGTTAA<br>TGCAGCCAATGTTTACCTTAAACATGGAGGAGGTGTTGCAGGAGCCTTAAATAAGGCTA<br>CTAACAATGCCATGCAAGTTGAATCTGATGATTACATAGCTACTAATGGACCACTTAAAG<br>TGGGTGGTAGTTGTGTTTTAAGCGGACACAATCTTGCTAAACACTG
FLUAV	gaaatTAATACGACTCACTATAgggGTAAGAGAATGAAGCTCCGGACACAAATACCTGCAG<br>AAATGCTAGCAAGCATTGACCTGAAGTATTTCAATGAATCAACAAGGAAGAAAATTGAG<br>AAAATAAGGCCTCTTCTAATAGATGGCACAGCATCATTGAGCCCTGGAATGATGATGGG<br>CATGTTCAACATGCTAAGTACAGTTTTAGGAGTCTCGATACTGAATCTTGGACAAAAGAA<br>ATACACCAAGACAACATACTGGTGGGATGGGCTCCAATCCTCAGACGATTTTGCCCTCA<br>TAGTGAATGCACCAAATCATGAGGGAATACAAGCAGGAGTGGATAGATTCTATAGGACC<br>TGCAAGTTAGTGGGAATCAACATGAGCAAAAAGAAGTCCTATATAAATAAAACAGGGAC<br>ATTTGAATTCACTAGCTTTTTTTATCGATATGGATTTGTGGCTAATTTTAGCATGGAGCT
FLUBV	gaaatTAATACGACTCACTATAgggAAGGCTCAAATACCTTGTCCTGATCTGTTCAGCATAC<br>CATTAGAAAGATATAATGAAGAAACAAGGGCGAAATTAAAAAGGCTGAAGCCATTCTTC<br>AATGAAGAAGGAACAGCATCTTTGTCGCCTGGGATGATGATGGGAATGTTTAATATGCT<br>ATCTACCGTGTTGGGAGTAGCAGCACTAGGCATCAAAAACATTGGAAACAAGGAATACT<br>TATGGGATGGACTGCAATCTTCTGATGATTTTGCTTTGTTTGTTAATGCAAAAGATGAAG<br>AAACATGTATGGAAGGGATAAATGATTTTTACCGAACATGTAAATTATTGGGAATAAACA<br>TGAGCAAAAAGAAAAGTTACTGTAACGAAACTGGAATGTTTGAATTTACAAGCATGTTCT<br>ATAGAGATGGATTTGTATCTAACTTTGCAATGGAAATTCCTTCATTTGGAGTTGCTG

HRSV	gaaatTAATACGACTCACTATAGGGGGGGCAAATATGGAAACATACGTGAACAAACTTCA<br>CGAAGGCTCCACATACACAGCTGCTGTTCAATACAATGTCCTAGAAAAAGACGATGATC<br>CTGCATCACTTACAATATGGGTGCCCATGTTCCAATCATCCATGCCAGCAGATTTACTTA<br>TAAAAGAACTAGCTAATGTCAACATACTAGTGAAACAAATATCCACA
HMPV	gaaatTAATACGACTCACTATAGGGGAGAAGACCAAGGGTGGTATTGTCAGAATGCAGG<br>GTCAACTGTTTACTACCCAAATGAGAAAGACTGTGAAACAAGAGGAGACCATGTCTTTT<br>GCGACACAGCAGCAGGAATTAATGTTGCTGAGCAATCAAAGGAGTGCAACATCAACAT<br>ATCCACTACAAATTACCCATGCAAAGTCAGCACAGGAAGACATCCTATCA
HPIV-3	gaaatTAATACGACTCACTATAGGGCAAAACACGGATCCCGGTATTTAGATGTCTTTCTAC<br>TCGGCTTCTTCGAGATGGAACGAATCAAAGATAAATACGGGAGTGTGAATGATCTTGAC<br>AGTGACCCGAGTTACAAAGTTTGTGGCTCTGGATCATTACCAATCGGACTGGCCAAATA<br>CACTGGGAATGACCAGGAATTATTACAGGCTGCAACCAAACTAGATA
HCoV-HKU1	gaaatTAATACGACTCACTATAgggTTATGCTGAGGTTCATGCTGAGCCTAAAGGTAAATAT<br>TCACAAAAAGCTTATGCTTTACTTAGACAATATCGTGGTATTAAACCCGTACTTTTTGTA<br>GACCAGTATGGTTGTGACTATTCTGGTAAATTAGCAGATTGTCTTCAAGCTTATGGTCAT<br>TATTCTTTGCAAGATATGAGACAAAAGCAGTCTGTATGGCTTGCCAATTGTGACTTTGAT<br>ATTGTAGTGGCTTGGCATGTAGTTCGTGATTCACGATTTGTTATGCGCCTGCAGACTAT<br>AGCTACTATTTGTGGTATTAAATATGTTGCACAACCTACAGAAGATGTAGTAGATGGAGA<br>TGTAGTTATACGTGAACCTGTACATTTATTATCTGCTGATGCAATAGTTTTAAAGCTTCCT<br>AGTTTGATGAAAGTTATGACTCATATGGATGATTTTTCTATTAAATCTATATATAATGTTG<br>ATTTGTGTGATTGTGGTTTTGTTATGCAGTATGGTTATGTAG
HCoV-NL63	gaaatTAATACGACTCACTATAgggCTTGGTACTACAGGTCATACTTTGAAGTCTGGTTGCA<br>AACTTATTAATGCTAAGCCGCCTAAATATTCTTCTAAGGTTGTTTTGAGTGGTGAATGGA<br>ATGCTGTGTATAGGGCGTTTGGTTCACCATTTATTACAAATGGTATGTCATTGCTAGATA<br>TAATTGTTAAACCAGTTTTCTTTAATGCTTTTGTTAAATGCAATTGTGGTTCTGAGAGTTG<br>GAGTGTTGGTGCATGGGATGGTTACTTATCTTCTTGTTGTGGCACACCTGCTAAGAAAC<br>TTTGTGTTGTTCCTGGTAATGTCGTTCCTGGTGATGTGATCATCACCTCAACTAGTGCT<br>GGTTGTGGTGTTAAATACTATGCTGGCTTAGTTGTTAAACATATTACTAACATTACTGGT<br>GTGTCTTTATGGCGTGTTACAGCTGTTCATTCTGATGGAATGTTTGTGGCATCATCTTCT<br>TATGATGCACTCTTGCATAGAAATTCATTAGACCCTTTTTGCTT
HCoV-OC43	gaaatTAATACGACTCACTATAgggAGCACTGACACTACTGTTCAATCAAAAGATACTAATT<br>TTTTAAACGGGTTCGGGGTGCGAGTGTAGATGCCCGTCTCGTACCCTGCGCCAGTGGT<br>TTATCTACTGATGTACAATTAAGGGCATTTGATATTTACAATGCTAGTGTTGCTGGCATT<br>GGTTTACATTTAAAAGTTAATTGTTGCCGTTTTCAGCGTGTTGATGAGAACGGTGATAAA<br>TTAGATCAGTTCTTTGTTGTTAAGAGGACAGATCTGACTATATATAATAGAGAGATGAAA<br>TGCTATGAGCGTGTAAAAGATTGTAAGTTTGTGGCTGAACACGATTTCTTTACATTTGAT<br>GTAGAAGGTAGTCGTGTGCCACACATTGTACGCAAGGATTTAACAAAGTATACTATGTT<br>GGATCTTTGCTATGCATTGCGACATTTTGATCGCAATGATTGCATGCTGCTTTGTGACAT<br>TCTCTCTATATATGCTGGTTGTGAACAATCCTACTTTACTAAGAAG
RNase P	gaaatTAATACGACTCACTATAgggATGGGACTTCAGCATGGCGGTGTTTGCAGATTTGGA<br>CCTGCGAGCGGGTTCTGACCTGAAGGCTCTGCGCGGACTTGTGGAGACAGCCGCTCA

	CCGTGAGTTGCCCCGGCTTCGCGCCTGGCCAACCTCATGCCACCCAGACCATCGGGC<br>CACACTCCGGAGTAACTATTTCCTGATGGGTCTCGGTCAGGTCTCCCAGAGTCTCTGG<br>GATGTCCCTGGAGGCTGATGCCCGCCGAGGTGTTGGTCTGAT
